# The epidemiological benefit of pyrethroid–pyrrole insecticide treated nets against malaria: an individual-based malaria transmission dynamics modelling study

**DOI:** 10.1016/S2214-109X(24)00329-2

**Published:** 2024-11-20

**Authors:** Thomas S Churcher, Isaac J Stopard, Arran Hamlet, Dominic P Dee, Antoine Sanou, Mark Rowland, Moussa W Guelbeogo, Basiliana Emidi, Jacklin F Mosha, Joseph D Challenger, Adrian Denz, Andrew Glover, Giovanni D Charles, Emma L Russell, Rich Fitzjohn, Pete Winskill, Christen Fornadel, Tom Mclean, Peder Digre, Joseph Wagman, Frank Mosha, Jackie Cook, Martin C Akogbéto, Luc S Djogbenou, Hilary Ranson, Philip McCall, Alphaxard Manjurano, Sagnon N’Falé, Natacha Protopopoff, Manfred Accrombessi, Corine Ngufor, Geraldine Foster, Ellie Sherrard-Smith

**Affiliations:** a School of Public Health, Imperial College London, London, UK; b Centre National de Recherche et de Formation sur le Paludisme, Ouagadougou, Burkina Faso; c Department of Disease Control, London School of Hygiene & Tropical Medicine, London, UK; d Department of Parasitology, National Institute for Medical Research, Mwanza Medical Research Centre, Mwanza, Tanzania; e Innovative Vector Control Consortium, Liverpool, UK; f PATH, Washington, DC, USA; g Centre de Recherches Entomologiques de Cotonou, Cotonou, Benin; h Institut Régional de Santé Publique, University of Abomey-Caliva, Abomey-Calavi, Benin; i Vector Biology, Liverpool School of Tropical Medicine, Liverpool, UK

## Abstract

**Background:**

Insecticide treated nets (ITNs) are the most important malaria prevention tool in Africa but the rise of pyrethroid resistance in mosquitoes is likely impeding control. WHO has recommended a novel pyrethroid–pyrrole ITN following evidence of epidemiological benefit in two cluster-randomised, controlled trials (CRTs). It remains unclear how effective more costly pyrethroid–pyrrole ITNs are compared with other tools, or whether they should be deployed when budgets are limited. We aimed to compare the epidemiological impact and cost-effectiveness of the mass distribution of pyrethroid–pyrrole ITNs relative to other ITNs over 3 years in different African settings.

**Methods:**

In this individual-based malaria transmission dynamics modelling study we characterise the entomological impact of ITNs using data from a systematic review of experimental hut trials from across Africa. This African entomological data was used to inform an individual-based malaria transmission dynamics model, which was validated against CRT results from Benin and Tanzania. The full impact of new ITNs was quantified for trial sites and simulation was used to project impact in different settings which were included within an accessible interface (the Malaria Intervention Tool) to support National Malaria Programmes to explore how vector control tools and budgets could be allocated across regions to avert the most cases.

**Findings:**

The model projects that distributing pyrethroid–pyrrole ITNs averted 65% (95% credible interval 48–74) of cases over 3 years in Tanzania, and 75% (28–93) in Benin. The model indicates that trials might have underestimated the benefits of switching ITNs by 12–16% over 3 years because participants stopped using trial-allocated nets. In moderate endemicity non-trial settings, pyrethroid–pyrrole ITNs are projected to reduce malaria prevalence by 25–60% and switching from pyrethroid-only ITNs is probably highly cost-effective in most locations given current prices, averting an additional 10–30% of cases.

**Interpretation:**

The benefit of pyrethroid–pyrrole ITNs varies by setting but is generally the most cost-effective indoor vector control intervention in Africa. National Malaria Programmes can strategise deployment to maximise impact. Entomological data could broadly predict epidemiological impact, although there are some inconsistencies, illustrating the challenge in capturing the dynamics across diverse settings.

**Funding:**

Unitaid, Bill & Melinda Gates Foundation, the UK Medical Research Council, Wellcome Trust, and the UK Foreign, Commonwealth & Development Office.

## Introduction

Widespread deployment of insecticide treated nets (ITNs) has prevented more malaria cases than any other intervention, with population weighted malaria prevalence in children halving from 33% (95% credible interval [CrI] 31–35) in 2000 to 16% (14–19) in 2015.^1^ Since 2019, these advances have stalled and there remains an intolerable disease burden, particularly in Africa. Multiple reasons contribute to this plateauing of cases,^2^ although the increase in mosquitoes resistant to insecticides is likely a key contributing factor. Over 200 million ITNs are distributed annually, but until 2019 all ITNs contained a single class of insecticide: the pyrethroids. Entomological data have shown how mortality of wild mosquitoes induced by pyrethroids has diminished substantially^3^ and pyrethroid resistant mosquitoes are now widespread across Africa.^4^ While the epidemiological impact of pyrethroid resistance on the benefit of pyrethroid long-lasting insecticidal nets (herein referred to as pyrethroid-only ITNs) is unclear,^5^, ^6^ new ITNs containing either a pyrethroid synergist piperonyl butoxide or an additional alternative insecticide showed statistically significant public health benefits in three different cluster-randomised, controlled trials (CRTs).^7^, ^8^, ^9^

Novel classes of ITNs need a WHO recommendation before they can be purchased by large international donors such as The Global Fund to Fight AIDS, Tuberculosis and Malaria. Pyrethroid–piperonyl butoxide ITNs received a conditional recommendation in 2017 after CRT evidence for greater effectiveness over pyrethroid-only ITNs and over 131 million were sent to Africa in 2022.^10^ The ability of piperonyl butoxide to synergise pyrethroids diminishes at high levels of pyrethroid resistance, so novel ITNs are urgently needed. Over the past decade a new ITN has been developed by combining pyrethroid insecticide with chlorfenapyr, a pro-insecticide of the pyrrole class, which targets mitochondrial respiratory pathways.^11^, ^12^, ^13^, ^14^, ^15^ Pyrethroid–pyrrole ITNs have shown epidemiological benefit over other pyrethroid-only ITNs in CRTs in Tanzania^9^ and Benin^8^ and received a full WHO recommendation in 2023.^16^ The total epidemiological benefit of pyrethroid–pyrrole ITNs is unclear because all trial arms distributed ITNs without removing currently owned nets (it is unethical to remove current standard of care); and the advantage of switching to new ITNs might exceed that observed in the CRTs, because trial nets tended to be replaced with alternatively sourced pyrethroid-only ITNs throughout the follow-up period. For example, in Tanzania, ITN use remained high, although in some instances fewer than 50% of people had trial ITNs 2 years into the study, diminishing the difference between arms.^17^ Decisions on the ITN type to deploy should be made on the basis that all people switch to the new ITN.

**Research in context**

**Evidence before this study**

Experimental hut trials are complex biological assays which measure the impact of insecticide treated nets (ITNs) on mosquitoes in real-life settings. We searched MEDLINE using the terms “experimental hut trial*”, “pyrrole”, “model” and “net” or “long lasting insecticidal net”, for review articles published from database inception up until Oct 1, 2021, with no language restrictions, but no studies were identified. ITNs have prevented more cases of and deaths from malaria than any other control intervention. ITNs primarily work by killing mosquitoes, but all WHO recommended ITNs until 2023 have contained a single class of insecticide, the pyrethroids, and pyrethroid resistant mosquitoes are now widespread throughout Africa and beyond, impeding control. New pyrethroid–pyrrole ITNs, containing a second insecticide, have been developed over the past decade, and switching to these nets has been shown to significantly reduce malaria burden in cluster-randomised, controlled trials (CRTs) in Benin and Tanzania. WHO has recommended the deployment of pyrethroid–pyrrole ITNs since 2023 based on their performance in empirical trials. Both entomological and epidemiological performance in standardised bioassays and CRTs indicate superiority to traditional pyrethroid-only ITNs, but the magnitude of the entomological benefit varies substantially. It remains unclear where they should be used instead of other ITNs as better products tend to be more expensive and budgets are severely limited. The added benefit of new nets over the current standard of care (pyrethroid-only ITNs) and those recommended in areas of pyrethroid resistant mosquitoes will vary according to the socioenvironmental and epidemiological context.

**Added value of this study**

Experimental hut trial data were collated from across Africa and used within a transmission dynamics mathematical model of malaria. The framework reliably predicted CRT results in Benin and Tanzania and showed how the empirical evidence from these CRTs potentially underestimated the full benefit of switching to pyrethroid–pyrrole ITNs due to incomplete retention of the trial ITNs. Simulation results representative of a range of entomological and epidemiological situations in Africa are included within a free online tool that enables decision makers to explore the most impactful and cost-effective choice of indoor vector control interventions against malaria, both within a region, but also across multiple areas with differing environments and prices.

**Implications of all the available evidence**

There is no longer a one-size-fits-all approach to malaria vector control and national programmes might consider tailoring decisions based on mosquito susceptibility, disease epidemiology, and product price. Over 200 million ITNs are distributed each year, and currently pyrethroid–pyrrole ITNs are likely to be the most cost-effective ITN in most situations in Africa. Our online tool provides a validated framework to support local decision makers to make the most appropriate indoor vector control intervention mix for their regions given local environments, prices, and goals.

There is no one-size-fits-all approach for malaria control. The best interventions to deploy will depend upon National Malaria Programme goals and associated costs, because budgets are always limited and more effective interventions are generally more costly, which could limit deployment. The impact of different ITNs will vary according to the entomological and epidemiological setting in which they are introduced. A WHO recommendation requires evidence of epidemiological benefit and does not consider relative effectiveness or provide guidance on use. Mathematical models can extrapolate CRT results to areas with differing mosquito vectors, disease endemicity, and history of control interventions. The mathematical models can provide a framework to explore what is the most cost-effective set of interventions in a region given local price information. Accessible interfaces can be developed that present model outputs representative of generic sites with defined characteristics, or specific locations.^18^ The first interface to contain widely used vector control options is the Malaria Intervention Tool (MINT), which assesses the potential effectiveness of the mass implementation of pyrethroid-only ITNs, pyrethroid–piperonyl butoxide ITNs, or indoor residual spraying (IRS) of insecticide,^19^ and whose use has been supported by WHO.^20^

We aimed to compare epidemiological impact and cost-effectiveness of the mass distribution of pyrethroid–pyrrole ITNs relative to pyrethroid-only ITNs (the current standard of care) over 3 years in different African settings. We explored whether mosquito data can be used to predict the epidemiological impact of the mass distribution of pyrethroid–pyrrole ITNs on *Plasmodium falciparum* malaria by comparing model results to CRT empirical data from Benin and Tanzania.^8^, ^9^ Finally, we aimed to extrapolate results to diverse settings representative of sub-Saharan Africa and investigate how budgets can be optimised over different settings to support decision making.

## Methods

### Study design and background

In this individual-based malaria transmission dynamics modelling study we compared the epidemiological impact and cost-effectiveness of the mass distribution of pyrethroid–pyrrole ITNs relative to other ITNs over 3 years in different African settings using a statistical framework that quantifies the entomological impact of different ITNs.

No ethical approval was required for this secondary modelling analysis. Two previously published CRTs from Tanzania^9^ and Benin^8^ that were used in model validation received their own ethical clearance (appendix 1 p 13).

### Data sources

Experimental hut trials are complex real-world entomological assays conducted in specially designed structures containing volunteers who sleep under different ITNs, to determine the efficacy and operational acceptability of ITNs.^21^ Wild, free-flying mosquitoes naturally enter huts and differences in the numbers of caught, dying, and blood-feeding mosquitoes between the study groups are used to estimate the entomological efficacy of an ITN. These house-level bioassays could be used to infer the epidemiological benefit of the mass distribution of the different types of ITNs, because transmission dynamic mathematical models parameterised with these data are broadly able to recreate the epidemiological results of ITN CRTs.^22^

In a previous systematic review^23^ Nash and colleagues characterised the statistical relationship between mosquito mortality induced by a WHO discriminating dose bioassay tube test—a measure of resistance in wild mosquitoes—and with the probable outcomes of a mosquito seeking a blood meal in hut trials up until April 30, 2019 (appendix 1 pp 3–12). We updated this review with new unpublished and published results investigating the entomological impact of pyrethroid–pyrrole ITNs to Nov 1, 2023. We searched the same databases as Nash and colleagues using the same inclusion and exclusion criteria, and unpublished data were requested from authors of identified publications (appendix 1 p 4; data summarised in appendix 2).

Briefly, using the statistical framework developed by Nash and colleagues, and the data obtained from our updated systematic review we quantified the relationship between (1) mortality in the tube test and induced by pyrethroid ITNs in experimental hut trials (appendix 1 p 5), (2) induced mortality by pyrethroid-only ITNs and next-generation ITNs (appendix 1 p 26), and (3) induced mortality and other hut trial outcomes (successful feeding; appendix 1 p 5). Data were restricted to unwashed WHO recommended ITN products. Two brands of pyrethroid–pyrrole ITNs were included (Interceptor G2, BASF, Ludwigshafen, Germany; and PermaNet Dual, Vestergaard, Lausanne, Switzerland), and in these comparisons, mortality was assessed over 72 h. Different levels of uncertainty are explored, using either a binomial model (mean percentage killed) or a beta-binomial model (probability an individual mosquito dies), which provides greater uncertainty (appendix 1 pp 6–9). Datapoints outside the measurement error of a single binomial trend are classified as being outliers using methods developed by Challenger and colleagues.^24^ The model-simulated ITN efficacy was parameterised,^22^, ^23^ and is detailed in appendix 1 (pp 4–12). Given that pyrethroid–pyrrole ITN durability over their 3-year life expectancy is unknown, we assumed that the ability of pyrethroid–pyrrole ITNs to kill mosquitoes wanes at the same rate as a pyrethroid-only ITN exhibiting the same level of hut trial mortality.

### Model outline and parameters

The mechanistic transmission dynamics model of *P falciparum* malaria has been described previously^22^, ^25^ with all code freely available online. Briefly, the human component of the model was individual-based and stochastic, and incorporated immunity, age structure, and heterogeneity in the mosquito bites received. People are born susceptible to malaria infection, which is dependent on mosquito biting rate, infectivity, and an individual's level of pre-erythrocytic immunity, all of which are influenced by the history of malaria control.

Model projections were validated against CRT results from Tanzania^9^ and Benin^8^ by parameterising the model according to site entomology, history of vector control, and adjusting mosquito abundance so that baseline parasite prevalence matched the observed estimate for each trial arm given the age cohorts and diagnostics used. The model then projects forward for each trial arm with observed ITN use and type changing over time (ie, trial nets being replaced with aged pyrethroid-only ITNs as observed empirically). Projections were assessed by their ability to capture the observed change in trial measured prevalence and difference between arms was calculated as previously defined.^22^ Simulations were then rerun keeping all other parameters constant, but either (1) having no new mass campaign (counterfactual scenario where existing nets are lost over time) or (2) assuming people continue to use trial ITNs (ie, people who use an ITN continue to use the trial ITN of the appropriate age instead of swapping it for another net type; appendix 1 p 15).

MINT simulations were updated with new parameters for pyrethroid–piperonyl butoxide and pyrethroid–pyrrole ITNs with assumptions about combinations of interventions remaining consistent (appendix 1 pp 19–22). The interface was adjusted to allow up to 15 different geographical regions to be simultaneously parameterised with local settings and price information. The impact of each intervention on clinical cases was estimated using the simulation model for each intervention package across all regions and the strategy that maximised the total number of mean cases averted within the defined budget (considering the population size of each region) was identified using the ompr package in R.^26^

### Role of the funding source

The funders of the study had no role in study design, data collection, data analysis, data interpretation, or writing of the report.

## Results

The updated review of new unpublished and published results investigating the entomological impact of pyrethroid–pyrrole ITNs to Nov 1, 2023 provided 26 comparisons between pyrethroid–pyrrole and pyrethroid-only nets from 26 studies, and 41 comparisons between pyrethroid–piperonyl butoxide and pyrethroid-only ITNs from 41 studies, with 96 studies in the full systematic review (appendix 2).

**Figure 1:**
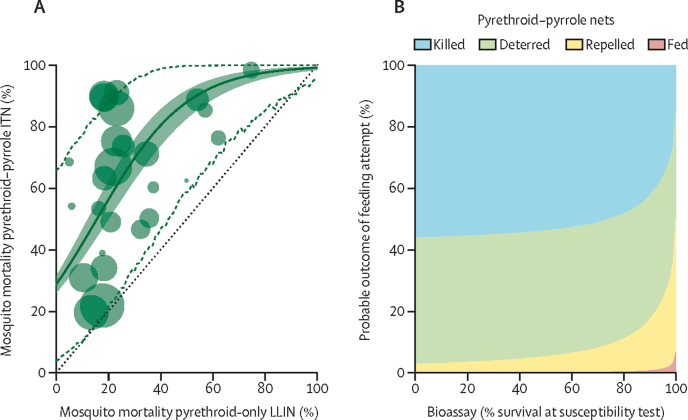
Summary of the entomological efficacy of pyrethroid–pyrrole ITNs (A) Experimental hut trials are used to assess the increased mosquito mortality caused by pyrethroid–pyrrole ITNs compared to pyrethroid-only LLINs. Points show results of a systematic review of trials comparing two nets over 72 h, and the point size indicates the number of mosquitoes caught (ie, the larger the point size the higher the number of mosquitoes). The solid green line indicates the best fit model and the green shaded area indicates 95% credible intervals for the uncertainty in this best fit line using the binomial function. The green dotted lines denote the lower and upper estimate of efficacy of the individual pairs of datapoints (approximately 90% of all comparisons fall within this region, using the beta-binomial association) and indicate the extreme uncertainty in the difference between the two ITNs. The black line indicates the region where both ITNs induce the same mortality. (B) A summary of the predicted outcome of a single mosquito feeding attempt and how these change with the level of pyrethroid resistance (as defined by the percentage of mosquitoes surviving 24 h following exposure in a discriminating dose bioassay). Mosquitoes are predicted to be killed (blue region), to be deterred from entering the hut (green), exit without feeding (yellow), or to successfully blood-feed (red). The mean estimate is shown, and the variability of eight different iterations from the posterior draws of the fitting process is in appendix 1 (p 27). Equivalent figures for pyrethroid-only and pyrethroid–piperonyl butoxide ITNs are provided in appendix 1 (pp 28–29). ITN=insecticide treated net. LLIN=long-lasting insecticidal net.

Data from our systematic review suggested that unwashed pyrethroid–pyrrole ITNs kill substantially more mosquitoes entering hut trials than pyrethroid-only ITNs. The added benefit of unwashed pyrethroid–pyrrole ITNs varies between settings, with mortality ranging from a 2% to a 98% increase in mosquitoes dying over 72 h (figure 1A). The statistical model indicates a clear association between pyrethroid-only and pyrethroid–pyrrole ITN induced mortality. The mean best fit line indicates that when pyrethroid-only nets kill 50% of mosquitoes entering the hut the pyrethroid–pyrrole net is predicted to kill 87% (95% CrI using a binomial model 83–92). This percentage is projected to diminish in areas with very high pyrethroid resistance; our model predicted that 59% (53–66) of mosquitoes are killed by the pyrethroid–pyrrole net in huts in sites where pyrethroid-only nets kill only 20% of mosquitoes. Mean results indicate pyrethroid–pyrrole nets always outperform pyrethroid–piperonyl butoxide ITNs (appendix 1 p 26), although there is considerable uncertainty.

The increase in mosquito mortality caused by pyrethroid–pyrrole ITNs is highly variable, with 14 (56%) of the 25 studies that compared pyrethroid–pyrrole ITNs with pyrethroid-only ITNs being identified as outliers of a single binomial trend (ie, point estimates fall outside the 95% CrI range predicted for the measurement error of the assay; appendix 1 p 30). This variability is substantially greater than for pyrethroid–piperonyl butoxide ITNs, where only five (28%) of 18 studies were predicted to be outliers (appendix 1 p 30). Other measurements including the level of deterrence and blood-feeding inhibition induced by pyrethroid–pyrrole ITNs are consistent with results for pyrethroid-only ITNs, which induce similar levels of deterrence and blood-feed inhibition for the same level of hut trial survival (appendix 1 p 5). These results enable the entomological impact of pyrethroid–pyrrole ITNs to be summarised according to the level of pyrethroid resistance (figure 1B).

**Figure 2:**
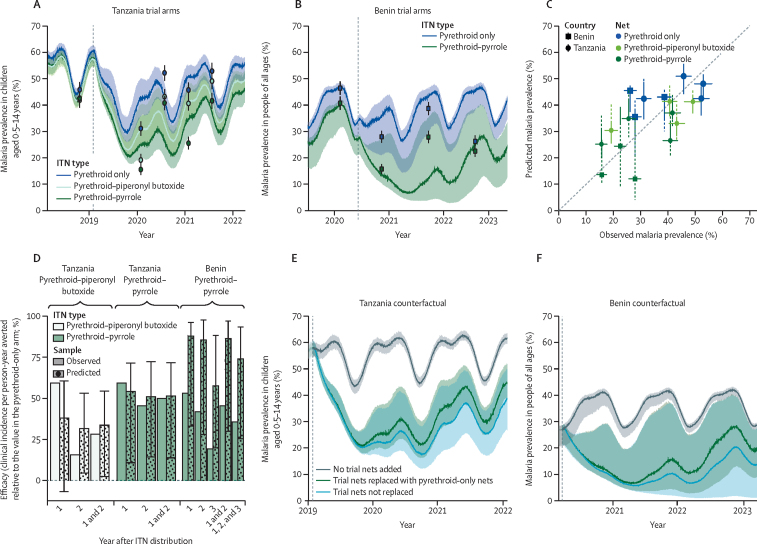
The ability of the model to predict epidemiological impact of novel ITNs (A, B) Changes in malaria prevalence following ITN distribution in the Tanzania and Benin cluster-randomised, controlled trials. Points indicate observed disease prevalence (with associated vertical lines indicating 95% CrIs) and solid lines show model projections (different age groups in each study) for pyrethroid-only, pyrethroid–piperonyl butoxide, and pyrethroi–pyrrole ITNs. The vertical dashed line indicates when the ITNs were introduced. (C) Comparison of model predictions and observed data for the trial data collection time points indicate that the model broadly captures changes in disease prevalence. Dashed vertical lines indicate beta-binomial uncertainty and solid vertical lines are binomial model uncertainty. The solid horizontal lines indicate 95% CrIs for observed data and the dashed diagonal line shows the equivalence line. (D) Observations and model predictions on the ability of ITNs to reduce clinical incidence. (E, F) Counterfactual model scenarios showing malaria prevalence if no new ITNs were distributed, the observed situation where trial nets were replaced by pyrethroid-only ITNs and the hypothetical situation where pyrethroid–pyrrole ITNs were replaced with other pyrethroid–pyrrole ITNs. The shaded areas around lines in A, B, E, and F, and the vertical lines in C and D indicate uncertainty in projections caused by uncertainty in ITN efficacy parameters as estimated using the beta-binomial model (dashed line in figure 1A). Figures with the lower binomial uncertainty are in appendix 1 (p 31). CrI=credible interval. ITN=insecticide treated net.

The transmission dynamics models parameterised with the meta-analysis of experimental hut trial data was broadly able to recreate the results of different arms of the CRTs in Tanzania (figure 2A) and Benin (figure 2B). Models could predict initial changes in malaria prevalence with high accuracy, but later timepoints were predicted less well, overestimating prevalence at 24 months in rapid diagnostic test prevalence among children aged 0·5–14 years in Tanzania but underestimating all-age prevalence in Benin at 18 months (figure 2C). The model's ability to predict the relative reduction in incidence was good for Tanzania but was less reliable for Benin, where it predicted a 74% (95% CrI 26–94) reduction in clinical incidence between the pyrethroid-only and pyrethroid–pyrrole groups over 3 years; however, only a 36% (17–50) reduction was observed. The model predicted the results of the other trial arms reliably (figure 2A–C), showing how pyrethroid-only ITNs provide only initial protection, with pyrethroid–piperonyl butoxide ITNs having intermediate efficacy. The full variability in the entomological efficacy of the different ITNs (figure 1A) was predicted to result in substantial uncertainty in epidemiological predictions (figure 2A–C; appendix 1 p 35).

The first 2 years of the Tanzania trial recorded a 50% (95% CrI 37–86) reduction in malaria cases by switching from pyrethroid-only to pyrethroid–pyrrole ITNs (table 1). The model predicted a 52% (14–72) reduction (mean reduction of prevalence in children aged 0·5–14 years of 28% [18–39]), although the percentage of cases averted would have been 62% (38–71) if people had continued to use pyrethroid–pyrrole ITNs (an estimated 33% [24–43] reduction in prevalence). The model predicts that over 36 months the cases averted by switching ITN would have been 16% higher in Tanzania (40% to 56%) and 12% higher in Benin (74% to 86%) if people had continued to use trial-allocated nets. This finding suggests that the CRT could be underestimating benefit of the novel ITN.

**Table 1:** Observed and predicted results of the cluster-randomised, controlled trials in Benin and Tanzania by time since ITN distribution

		**Tanzania**				**Benin**					
		Survey; observed (efficacy*)	Model derived; observed mixture of nets		Model derived; all nets the same		Survey; observed (efficacy*)	Model derived; observed mixture of nets		Model derived; all nets the same	
			Predicted (efficacy*)	Overall efficacy†	Predicted (efficacy*)	Overall efficacy†		Predicted (efficacy*)	Overall efficacy†	Predicted (efficacy*)	Overall efficacy†
**Malaria prevalence per intention-to-treat analysis protocol cohort**											
Pyrethroid-only ITN											
	6 months	..	..	..	..	..	0·28	0·36	24%	..	..
	12 months	0·31	0·42	32%	..	..	..	..	..	..	..
	18 months	0·52	0·43	25%	..	..	0·39	0·43	8%	..	..
	24 months	0·46	0·51	20%	..	..	..	..	..	..	..
	30 months	0·53	0·48	17%	..	..	0·26	0·45	4%	..	..
	0–18 months‡	..	..	..	..	..	..	0·35	18%	..	..
	0–24 months‡	..	0·42	27%	..	..	..	..	..	..	..
	0–36 months‡	..	0·45	23%	..	..	..	0·38	11%	..	..
Pyrethroid–piperonyl butoxide ITN											
	12 months	0·19 (38%)	0·30 (27%)	49%	0·28 (34%)	53%	..	..	..	..	..
	18 months	0·43 (17%)	0·33 (23%)	40%	0·29 (32%)	49%	..	..	..	..	..
	24 months	0·41 (11%)	0·41 (18%)	33%	0·35 (30%)	42%	..	..	..	..	..
	30 months	0·49 (7%)	0·41 (13%)	26%	0·36 (24%)	35%	..	..	..	..	..
	0–24 months‡	..	0·34 (20%)	38%	0·32 (25%)	42%	..	..	..	..	..
	0–36 months‡	..	0·37 (18%)	34%	0·33 (26%)	40%	..	..	..	..	..
Pyrethroid–pyrrole ITN											
	6 months	..	..	..	..	..	0·16 (44%)	0·14 (60%)	66%	0·13 (61%)	68%
	12 months	0·16 (50%)	0·25 (39%)	57%	0·22 (45%)	63%	..	..	..	..	..
	18 months	0·41 (22%)	0·27 (36%)	50%	0·22 (44%)	58%	0·28 (28%)	0·12 (72%)	69%	0·08 (81%)	79%
	24 months	0·26 (44%)	0·35 (29%)	42%	0·29 (43%)	52%	..	..	..	..	..
	30 months	0·42 (21%)	0·37 (22%)	33%	0·31 (34%)	43%	0·22 (14%)	0·24 (44%)	39%	0·16 (64%)	59%
	0–18 months‡	..	..	..	..	..	..	0·13 (61%)	63%	0·12 (64%)	66%
	0–24 months‡	..	0·30 (28%)	45%	0·27 (33%)	50%	..	..	..	..	..
	0–36 months‡	..	0·32 (27%)	42%	0·29 (34%)	48%	..	0·15 (58%)	56%	0·12 (68%)	66%
**Clinical incidence per intention-to-treat analysis protocol cohort**											
Pyrethroid-only ITN											
	0–12 months	0·32	0·70	69%	..	..	0·77	0·92	58%	..	..
	12–24 months	0·57	1·31	43%	..	..	1·19	1·91	16%	..	..
	24–36 months	..	1·95	12%	..	..	1·19	2·26	−3%	..	..
	0–24 months‡	0·46	0·99	56%	..	..	1·03	1·42	35%	..	..
	0–36 months‡	..	1·33	41%	..	..	1·09	1·70	24%	..	..
Pyrethroid–piperonyl butoxide ITN											
	0–12 months	0·13 (59%)	0·42 (38%)	81%	0·39 (45%)	83%	..	..	..	..	..
	12–24 months	0·48 (16%)	0·88 (32%)	63%	0·66 (50%)	72%	..	..	..	..	..
	24–36 months	..	1·65 (14%)	27%	1·23 (35%)	44%	..	..	..	..	..
	0–24 months‡	0·33 (28%)	0·66 (34%)	71%	0·54 (48%)	77%	..	..	..	..	..
	0–36 months ‡	..	0·98 (25%)	57%	0·77 (42%)	67%	..	..	..	..	..
Pyrethroid–pyrrole ITN											
	0–12 months	0·13 (59%)	0·31 (54%)	86%	0·28 (58%)	87%	0·36 (53%)	0·09 (88%)	95%	0·08 (89%)	96%
	12–24 months	0·31 (46%)	0·61 (51%)	73%	0·42 (65%)	82%	0·69 (42%)	0·27 (86%)	85%	0·13 (92%)	93%
	24–36 months	..	1·38 (29%)	36%	0·96 (49%)	56%	0·96 (19%)	0·93 (58%)	49%	0·48 (78%)	73%
	0–24 months‡	0·23 (50%)	0·46 (52%)	79%	0·35 (62%)	84%	0·56 (46%)	0·19 (87%)	90%	0·11 (92%)	94%
	0–36 months‡	..	0·77 (40%)	65%	0·55 (56%)	75%	0·70 (36%)	0·45 (74%)	75%	0·23 (86%)	87%

ITN=insecticide treated net.

* Percentage efficacy compared with the pyrethroid-only arm at that timepoint.

† Percentage efficacy compared with counterfactual scenario where there was no mass distribution of ITNs and existing nets were lost over time. In all efficacy columns empirical data are compared with empirical data, and model projections are compared with model projections.

‡ Mean estimates over time period: for empirical data this was a mean of the point estimates, whereas for models this was the mean across every simulated day of the time period. All model values show best guess estimate and were calculated with the central estimate of pyrethroid resistance. All model generated values shown use the beta-binomial model. Estimates of the uncertainty in model predictions are provided in appendix 1 (p 36).

Counterfactual simulations indicate that halting ITN distribution would cause a gradual rise in malaria prevalence, illustrating that old pyrethroid-only ITNs were still providing some epidemiological benefit (figure 2E, F). Overall, in Tanzania the model predicts that mass use of pyrethroid–pyrrole ITNs compared with no net distributions averted 65% (95% CrI 48–74) of malaria cases over 3 years compared with 41% (32–53) for pyrethroid-only ITNs and 57% (45–67) for pyrethroid–piperonyl butoxide ITNs. Similar epidemiological improvements were predicted in the Benin trial, where maintaining pyrethroid–pyrrole ITN coverage was predicted to increase the percentage of cases averted compared with the scenario whereby pyrethroid–pyrrole ITNs are replaced with pyrethroid-only ITNs from 75% (28–93) to 87% (31–99) overall (table 1).

**Figure 3:**
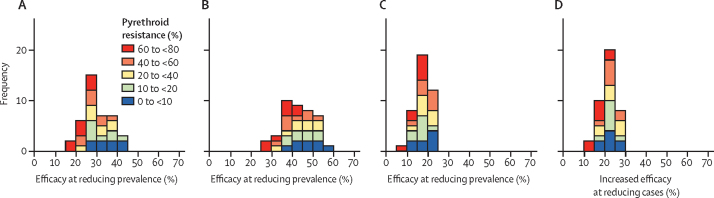
Projected epidemiological benefit of pyrethroid–pyrrole over pyrethroid-only ITNs in different settings in Africa Model estimates of the efficacy of pyrethroid-only (A) and pyrethroid–pyrrole (B) ITNs to reduce mean malaria prevalence over 3 years following a mass distribution campaign (by microscopy, all ages). The figure shows the frequency distribution of efficacy estimates from 80 model simulations varying mosquito bionomics, pyrethroid resistance level and net type (in a site with 40% malaria prevalence, 40% historical pyrethroid-only ITN use, a mosquito population with less than 90% resistance [making the assumptions on resistance outlined in figure 1 and appendix 1 pp 4–12], and no history of indoor residual spraying). Efficacy was calculated as the reduction in malaria prevalence resulting from a new ITN campaign which initially achieves 80% usage compared to a scenario when no campaign takes place. Bars are coloured according to the level of pyrethroid resistance in the local mosquito population. The projected percentage relative increase in efficacy from switching from pyrethroid-only to pyrethroid–pyrrole ITNs on mean prevalence (C) and clinical cases averted (D) is shown. ITN=insecticide treated net.

Results from 2 540 160 simulations varying mosquito bionomics, microscopy-positive malaria prevalence in children younger than 5 years, history of vector control (ITN use and level of resistance), and IRS are provided and can be viewed online in MINT. These simulations enable exploration of possibly more realistic intervention usage than those observed under trial conditions and allow the user to explore possible future scenarios when impact and prices might differ. MINT outputs are compared to trial simulations in matched age cohorts (appendix 1 p 32) indicating predicted epidemiological impacts are reasonable in this use case (appendix 1 p 24). Overall, in a moderate transmission setting, across a range of mosquito bionomics, pyrethroid-only ITNs are predicted to reduce disease prevalence by 15–45% compared with halting ITN campaigns (figure 3A). Pyrethroid–pyrrole nets are projected to have a substantially bigger impact, reducing prevalence by 25–60% (figure 3B). Switching from pyrethroid-only to pyrethroid–pyrrole ITNs is projected to reduce malaria prevalence by 5–25% (figure 3C), or cases by 10–30% (figure 3D). The projected advantage of pyrethroid–pyrrole over pyrethroid–piperonyl butoxide is shown in appendix 1 (p 33) with a broader sensitivity analysis shown in appendix 1 (p 34).

**Figure 4:**
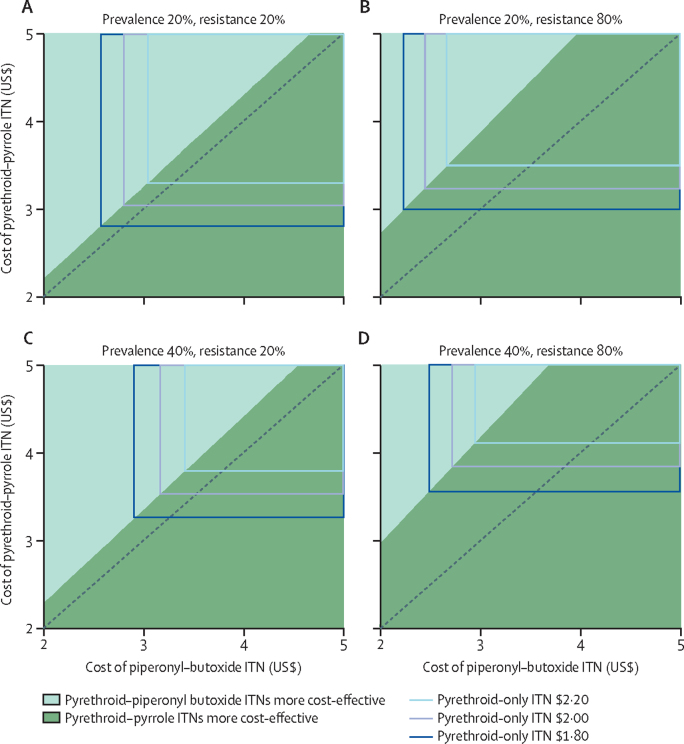
ITN cost-effectiveness decision making The most cost-effective net is identified as having the minimum cost per case averted. Three types of ITNs are contrasted for a different ecological setting to identify the most cost-effective net at a given price for each net. Decision-making plots are shown for different levels of baseline prevalence (top panels 20% and bottom panels 40%) and pyrethroid resistance (left panels 20% and right panels 80%). Coloured shading indicates the costs of pyrethroid–piperonyl butoxide (turquoise) and pyrethroid–pyrrole ITNs (green) at which this ITN is the more cost-effective. The area within superimposed overlapping blue boxes indicates the costs of pyrethroid–piperonyl butoxide and pyrethroid–pyrrole ITNs at which pyrethroid-only ITNs might be the most cost-effective net, at three indicative costs (dark blue $1·80, medium blue $2·00, and light blue $2·20). Within the blue boxes, more than one net will be most cost-effective at a given cost combination due to the two-dimensional structure of the plots. Cost-effectiveness calculations include net price, cost of net distribution ($2·75 per person) and a procurement buffer (7%) and assume 1·8 people per net. Costs are illustrative and do not necessarily represent current market price ranges. All plots assume mosquitoes have a human blood index of 87%, 97% of mosquitoes feeding when people are indoors, a historic ITN usage of 40% without IRS, and an expected ITN coverage of 80% with no IRS. Incremental cost-effectiveness ratios are in appendix 1 (p 35). IRS=indoor residual spraying. ITN=insecticide treated net.

Model projections indicated that pyrethroid–pyrrole nets were the more cost-effective ITNs, with a lower cost per case averted unless they were considerably higher in price. For example, in an area with 40% malaria prevalence and 80% pyrethroid resistance, if pyrethroid–piperonyl butoxide ITNs were US$2·90 then pyrethroid–pyrrole nets would need to be less than $4·09 to be the most cost-effective option (with pyrethroid-only ITNs needing to be $2·00 or less, each). This projection indicates that there are no simple thresholds above which one ITN type becomes more cost-effective, with local entomology, epidemiology, and existing disease control determining the most cost-effective option (figure 4).

MINT facilitates exploration of how interventions could be distributed across multiple settings to maximise mean cases averted. A theoretical illustration using realistic product prices for 2023^27^ is shown in table 2. In this illustration, annual long-lasting IRS averts the most cases but is too expensive to use in all regions, so is prioritised in areas of high pyrethroid resistance and high malaria prevalence. Reducing the overall budget across all regions shifts the most impactful choice of ITN and IRS, with all three ITN types recommended according to the overall budget available.

**Table 2:** Optimising malaria interventions across multiple regions

	**Maximum cost *vs* budget**	**Region A (population size 6200; malaria prevalence 26–35%; previous ITN coverage 80%; pyrethroid resistance 40%)**	**Region B (population size 3100; malaria prevalence 46–55%; previous ITN coverage 60%; pyrethroid resistance 60%)**	**Region C (population size 5200; malaria prevalence 36–45%; previous ITN coverage 40%; pyrethroid resistance 60%)**	**Total cases averted**	**Total cost, US$**
Strategy 1	100%	Pyrethroid–pyrrole ITN only	IRS only	Pyrethroid–pyrrole ITN only	13 683	$90 561
Strategy 2	95%	Pyrethroid–pyrrole ITN only	IRS only	Pyrethroid–piperonyl butoxide ITN only	13 239	$90 097
Strategy 3	90%	Pyrethroid-only ITN	IRS only	Pyrethroid-only ITN	11 514	$85 004
Strategy 4	85%	Pyrethroid–pyrrole ITN only	Pyrethroid–pyrrole ITN only	Pyrethroid–pyrrole ITN only	10 299	$47 407
Strategy 5	80%	Pyrethroid–pyrrole ITN only	Pyrethroid–pyrrole ITN only	Pyrethroid–pyrrole ITN only	10 299	$47 407

The strategy function of the Malaria Intervention Tool explored the most impactful combination of malaria vector control interventions depending on the characteristics of all regions and the total budget available (strategy 1), in addition to several lower cost options (strategies 2–5). In this theoretical example, the total vector control budget for three regions with diverse characteristics was US$95 000. With strategy 1, 13 683 cases of malaria could potentially be averted from the total population of 14 500 people, by using pyrethroid–pyrrole ITNs in regions A and C and IRS in region B; and 10 299 cases could potentially be averted for approximately $40 000 less by distributing pyrethroid–pyrrole ITNs everywhere. Strategy 2 indicates that pyrethroid–piperonyl butoxide ITNs in region C might be the most cost-effective option within budget, whereas strategy 3 suggests more cases would be averted using pyrethroid-only ITNs in some regions. All regions were assumed to have equivalent access to treatment, seasonal malaria transmission, high indoor biting (97% of *Anopheles* bites occur indoors), high human blood index (87% of bites are taken on people), no recent IRS coverage, and an expected potential coverage of 80% for both ITNs (following distribution) and IRS (achieved every year). Total costs assumed 1·8 people per net; a cost per net of $1·93 for pyrethroid-only ITNs, $2·60 for pyrethroid–piperonyl butoxide ITNs, and $2·75 for pyrethroid–pyrrole ITNs; an ITN distribution cost of $2·75 per person that is assumed to be the same for all types of net deployed; a 7% ITN procurement buffer (procurement of 107% of the required nets to allow for population data inaccuracies); and an annual cost of IRS of $5·73 per person. Care should be taken when interpreting these results as other factors not included in the modelling framework will need to be considered in the decision-making process. These simulations do not consider equity or insecticide resistance management, nor price inflations over time, which should also be considered in the decision-making process. IRS=indoor residual spraying. ITN=insecticide treated net.

## Discussion

The widespread use of pyrethroid–pyrrole ITNs is projected to avert substantially more malaria cases in Africa than pyrethroid-only or pyrethroid–piperonyl butoxide nets. Our study shows how the epidemiological benefit will vary substantially between settings. Currently the mass distribution of pyrethroid–pyrrole ITNs is likely to be the most cost-effective population-wide method of preventing malaria in Africa unless prices are considerably higher than alternative net types.

The ability of pyrethroid–pyrrole ITNs to kill mosquitoes appears to vary substantially between sites, although the meta-analysis of entomological data collected in experimental hut trials provides a reasonable correlate of the epidemiological protection provided by the mass distribution of pyrethroid–pyrrole ITNs. Models parameterised with this meta-analysis broadly predicted the changes in disease prevalence and incidence observed in Tanzania and Benin from the widespread use of different types of ITN. This finding is consistent with previous work showing the ability of hut trials to predict the benefit of pyrethroid-only ITNs, pyrethroid–piperonyl butoxide ITNs, and IRS, and further supports the use of these biological assays in product development and decision making.

Our study highlights how mathematical models can determine the full epidemiological impact of different ITNs. Future trials cannot remove community standard of care, so models are needed to safely explore counterfactual scenarios in which existing interventions are stopped or replaced with new interventions under consideration. Similarly, trial conditions might underestimate or overestimate intervention impact compared with real-world implementation. The two CRTs (Benin and Tanzania) examined in our study probably underestimated the potential epidemiological benefit of switching ITNs because many participants reverted to using pyrethroid-only nets during the trial.^8^, ^9^ Our model simulations suggest that if trial ITNs had been used throughout then overall the cases averted over pyrethroid-only ITNs would have been 16% higher in Tanzania and 12% higher in Benin, the exact value changing according to the time period and metric. These estimates of additional benefit are likely to be conservative as we assume that substituted pyrethroid–pyrrole nets are in the same condition as the ITNs they replace, whereas unused nets might be expected to perform better, further exaggerating the possible underestimation observed in the CRTs. The simulations also highlight the dangers of extrapolating trial results to the epidemiological benefit of rolling out the intervention at scale. For example, empirical point estimates indicate that swapping to pyrethroid–pyrrole ITNs reduced mean parasite prevalence by 46–59% in Tanzania and 19–53% in Benin, depending on the timing of data collection (table 1). This result is dependent on the age group and timings of the cross-sectional cohorts, both of which varied between studies. The simulation work suggests that in a moderate transmission area the benefit of switching would be between a 5% to 25% reduction in prevalence over the 3-year life expectancy of the net, depending on mosquito characteristics. Direct comparisons of products between different trials should be carefully considered unless site and trial characteristics are closely matched.^22^

There is room for improvement in the entomological data used for model parameterisation, and the modelling process itself. Experimental hut trial results can be highly variable, and it is unclear whether this is caused by the assay or differences between mosquito populations. The positive association between mortality induced by pyrethroid-only and pyrethroid–pyrrole ITNs was measurable but variable, and the predictive ability of the best fit model was reasonable. The high variability observed in pyrethroid–pyrrole ITNs could be caused by a reduced susceptibility to chlorfenapyr. A study published in 2021, based in 16 different African countries, showed widespread chlorfenapyr susceptibility using bottle bioassays,^28^ although studies in Cameroon, the Democratic Republic of the Congo, and Ghana indicated lower than expected mortality.^29^ The ability of experimental hut trials to capture the full impact of pro-insecticides, where mosquitoes must metabolise the insecticide into its active form, also remains unclear. Mosquito movement changes the rate of metabolism, so novel assays such as the video cone test,^30^ room-scale tracking, or ambient chamber test might improve reliability, as could genetic methods.^29^ The observed variability in hut trial results could have epidemiological impact as pyrethroid–pyrrole ITNs are deployed across Africa, so there is an urgent need to verify possible causes of heterogeneity.^31^

This study uses a meta-analysis of hut trial data to predict the results of the two trials and it would be interesting to see whether accuracy improves using hut trial data collected adjacent to the CRT site. The model appears to underestimate the impact of pyrethroid–pyrrole ITNs on malaria prevalence in the second year of the trial in Tanzania, but overestimated impact for the same year in Benin (resulting in a substantial overestimate of the impact on clinical cases). The cause of this contradicting discrepancy is unclear and highlights the large uncertainties in epidemiological projections. The high variability in the observed entomological efficacy causes considerable differences in projections of disease prevalence, and lack of knowledge of entomological difference within the different trial will further compound uncertainties. Estimates of absolute number of cases averted should be treated cautiously, although the relative difference between ITNs will probably be more reliable given their similar modes of action. The durability of pyrethroid–pyrrole ITNs remains unknown; data on naturally aged field nets are not currently available. Here we used data from new ITNs and assume insecticidal activity decays at the same rate to that observed in pyrethroid-only ITNs that induce a matched level of mosquito mortality. This assumption broadly agrees with CRT observations at later timepoints but the analysis needs repeating once data from field-aged ITNs are available.

Decisions about optimal vector control tools must consider current costs as prices are continually changing and budgets are limited. Tools such as MINT aim to help decision makers choose the best combination of interventions, although these projections should not be overly interpreted because results are very dependent on the inputted information and model assumptions. Users are encouraged to interpret results qualitatively and carefully consider the geographical scale over which decisions are made given the available data (appendix 1 pp 18–22). When inputted information is uncertain decision makers could explore a range of plausible scenarios and investigate whether this changes the most efficient intervention mix. Further work is needed to verify that the simplified settings in MINT are reasonable approximations for the diversity seen in Africa, and to include uncertainties in inputted information and model predictions in the decision-making process. Modelled results are no substitute for good local expertise and data, and final decisions must consider other factors including health equity, environmental impacts, public opinion, and insecticide resistance management (ie, minimising selection for chlorfenapyr resistance). Nevertheless, this framework provides an evidence base for the justification of further investment in vector control and the adoption of novel technologies in the fight against this deadly disease.

## Contributors

## Data sharing

The models used in this study and a summary of published data are available at https://github.com/EllieSherrardSmith/Mosq-Net-Efficacy. Simulations behind the MINT interface are downloadable from https://doi.org/10.5281/zenodo.8344312. Parameter estimates used in the transmission dynamics mathematical model for pyrethroid-only ITNs are in appendix 3, for pyrethroid–piperonyl butoxide ITNs are in appendix 4, and for pyrethroid–pyrrole ITNs are in appendix 5. The MINT user guide is in appendix 6.

## Declaration of interests

We declare no competing interests.
