# Supplementary material for: The epidemiological benefit of pyrethroid–pyrrole insecticide treated nets against malaria: an individual-based malaria transmission dynamics modelling study
Source: Lancet Glob Health. 2024 Nov 20;12(12):e1973–83. doi: 10.1016/S2214-109X(24)00329-2 (PMC11584316; doi:10.1016/S2214-109X(24)00329-2)
Supplement: Supplementary appendix 1 [file mmc1.pdf]

THE LANCET
Global Health

Supplementary appendix 1

This appendix formed part of the original submission and has been peer reviewed. We post it as supplied by the authors.

Supplement to: Churcher TS, Stopard IJ, Hamlet A, et al. The epidemiological benefit of pyrethroid–pyrrole insecticide treated nets against malaria: an individual-based malaria transmission dynamics modelling study. *Lancet Glob Health* 2024; **12:** e1973–83.

Appendix 1

for

The epidemiological benefit of pyrethroid–pyrrole insecticide treated nets against malaria: an individual-based malaria transmission dynamics modelling study

Author list

Thomas S. Churcher[1*], Isaac J. Stopard[1*], Arran Hamlet[1*], Dominic P. Dee[1], Antoine Sanou[2], Mark Rowland[3], Moussa W. Guelbeogo[2], Basiliana Emidi[4], Jacklin F. Mosha[4], Joseph D. Challenger[1], Adrian Denz[1], Andrew Glover[1], Giovanni D. Charles[1], Emma L. Russell[1], Rich Fitzjohn[1], Pete Winskill[1], Christen Fornadel[5], Tom Mclean[5], Peder Digre[6], Joe Wagman[6], Frank Mosha[4], Jackie Cook[3], Martin C Akogbéto[7], Luc S. Djogbenou[8,9], Hilary Ranson[9], Philip McCall[9], Alphaxard Manjurano[4], Sagnon N'Falé[2], Natacha Protopopoff[3,4], Manfred Accrombessi[3], Corine Ngufor[3,7], Geraldine Foster[9], Ellie Sherrard-Smith[1]

* Joint first authorship

Table of Contents

1 Supplementary methods 4

1.1 Characterising entomological impact 4

Table S1. The search terms used in systematic review previously presented by Nash et al. [1] 4

1.2 Association between susceptibility test and experimental hut bioassay mortality 4

Figure S1. Entomological data analysis. 5

1.3 Association between experimental hut mortality of different ITNs 5

1.3.2 Probable outcomes from an *Anopheline* mosquito feeding attempt 9

Table S2 Parameter estimates for the analysis of pyrethroid-pyrrole net efficacy 11

1.4 Entomological impact of ITNs 11

Table S3 Probabilities of mosquito feeding outcomes 12

1.5 Recreation of cluster randomised control trials 13

1.5.1 Data availability 13

1.5.2 Ethical approval 13

1.5.3 Insecticide-treated net (ITN) coverage 13

Figure S2 The decline in long-lasting insecticidal net use during trials 15

1.5.4 Model simulations 16

1.5.5 *Anopheles* mosquito bionomics 19

1.5.6 Seasonality in transmission 19

1.5.7 Model calibration 19

1.5.8 Reproducible code 19

1.5.9 MINT simulations 19

1.5.10 Comparison of MINT outputs and bespoke simulations 22

1.5.11 Cost effectiveness 25

1.5.12 Strategising across regions 25

2 Supplementary Figures 26

Figure S4. Uncertainty estimates for pyrethroid-pyrrole ITNs. 27

Figure S5. Uncertainty estimates for pyrethroid-only ITNs. 28

Figure S6. Uncertainty estimates for pyrethroid-PBO ITNs. 29

Figure S7. The association between mortality induced by different types of ITNs 30

Figure S8. The ability of the model to predict epidemiological impact 31

Figure S9. Comparison of MINT outputs and bespoke simulations 32

Figure S10. Projected epidemiological benefit of ITNs in different settings in Africa. 33

Figure S11. Sensitivity analysis of MINT simulations. 34

Figure S12. Incremental cost-effectiveness ratios for comparisons between different ITNs. 35

3 Supplementary Tables 36

Table S6. Estimates of different levels of uncertainty in model parasite prevalence estimates....36
4 List of Appendices....37
Appendix 2. Data used in the systematic review. 37
Appendix 3. Parameter estimates for pyrethroid-only ITNs....37
Appendix 4. Parameter estimates for pyrethroid-PBO ITNs....37
Appendix 5. Parameter estimates for pyrethroid-pyrrole ITNs....37
Appendix 6. MINT Version 2 user guide. 37
5 Full references (main paper and supplements)....38

1 Supplementary methods

1.1 Characterising entomological impact

It is unclear which class of insecticide treated net (ITN) should be deployed in areas where mosquitoes exhibit resistance to pyrethroid insecticide. Nash et al. [1] developed a statistical framework to assess the performance of ITNs against wild free-flying malaria vectors whose level of pyrethroid resistance is assessed using either a WHO tube or CDC cone discriminating dose bioassay. To do this, the researchers developed statistical methods to associate the probability of mosquito survival in the bioassay test with the probable outcome of a wild-type mosquito as she seeks a blood meal during as assessed in an experimental hut trial. Each experimental hut trial essentially represents a single blood-feeding attempt which can be used to parameterise transmission dynamics mathematical models. The previous review [registered on PROSPERO (CRD42019117858)] followed the PRISMA guidelines to document all experimental hut trials up to 30th April 2019. In the presented work, this review is updated to 01 November 2023 to include published and unpublished work on pyrethroid-pyrrole and pyrethroid-PBO ITNs. The same databases were searched using the same inclusion/exclusion criteria and unpublished data were requested from authors of identified publications. A full list of studies and data identified is collated in Appendix 2. Table S1 documents the search terms re-used here from the original review:

Table S1. The search terms used in the systematic review previously presented by Nash et al. [1]

Search	Search term
9	7 and 8
8	malaria.mp. [mp=title, abstract, original title, name of substance word, subject heading word, floating sub-heading word, keyword heading word, protocol supplementary concept word, rare disease supplementary concept word, unique identifier, synonyms]
7	5 and 6
6	(hut or huts).mp. [mp=title, abstract, original title, name of substance word, subject heading word, floating sub-heading word, keyword heading word, protocol supplementary concept word, rare disease supplementary concept word, unique identifier, synonyms]
5	1 or 2 or 3
3	(insecticide treated net* or ITN* or long lasting insecticidal net* or LLIN*).mp. [mp=title, abstract, original title, name of substance word, subject heading word, floating sub-heading word, keyword heading word, protocol supplementary concept word, rare disease supplementary concept word, unique identifier, synonyms]
2	Insecticide-Treated Bednets/
1	(indoor adj2 residual adj2 spray*) or IRS).mp. [mp=title, abstract, original title, name of substance word, subject heading word, floating sub-heading word, keyword heading word, protocol supplementary concept word, rare disease supplementary concept word, unique identifier, synonyms]

1.2 Association between susceptibility test mortality and experimental hut bioassay mortality

Following Nash et al.[1], it is assumed that the proportion of *Anopheline* mosquitoes that survive a WHO pyrethroid discriminating dose bioassay or a CDC susceptibility test bioassay is an approximate estimate for the level of pyrethroid resistance in a wild mosquito population. Unlike previous analyses data were limited to WHO recommended products[2], for pyrethroid-only nets (Olyset® Net, Interceptor®, PermaNet® 2.0, MiraNet®,

DuraNet®, MAGNet®, DawaPlus® 2.0, Yorkool®, PermaNet® 2.0, Royal Sentry®). The relationship between bioassay survival (denoted *R*) and the probability of a mosquito surviving 24 hours following exposure to a pyrethroid mosquito net (including pyrethroid-dipped nets (CTNs), and pyrethroid-only ITNs) in an EHT (l_1) has been previously described by a log-logistic function in[1] as,

$$(1 - l_1) = 1/(1 + R/\alpha_1^2)^{-\alpha_2^2}), \quad \text{(Equation S1)}$$

where α_1^2 = 0.86 (95CrI: 0.33 - 2.33) and α_2^2 = 0.27 (95CrI: 0.15 - 0.40) (Table S2). This relationship is shown in Figure S1A).

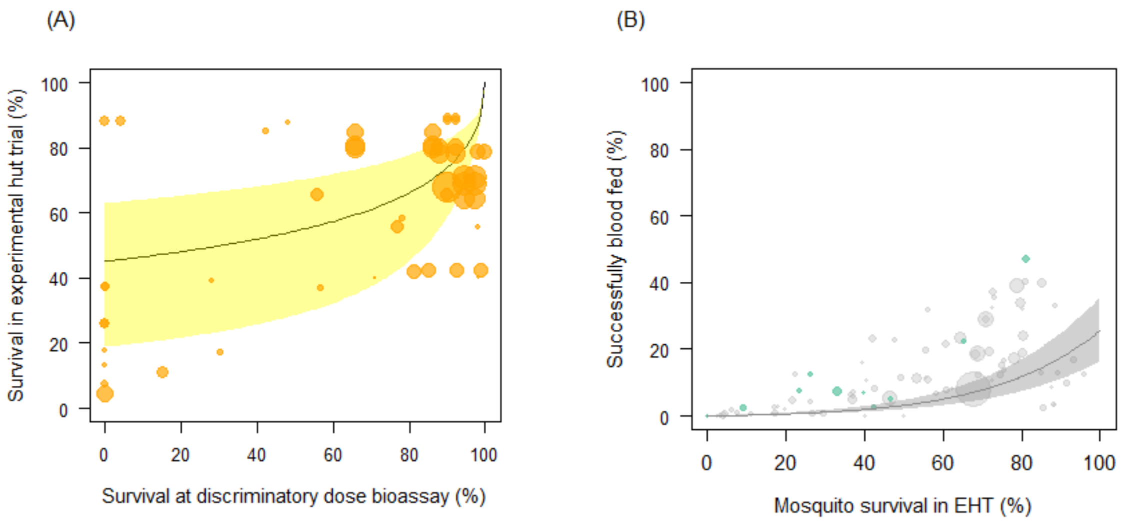

Figure S1. Entomological data analysis. (A) Systematic review of mosquito survival on exposure to a discriminatory dose of pyrethroid in the bioassay test is associated with survival of wild free-flying mosquitoes as observed in experimental hut trials. Hut type was restricted to East or West-African design. (B) The mosquito survival in experimental huts is associated with successful blood feeding, green points indicate pyrethroid-pyrrole ITN data whilst grey points indicate pyrethroid-only ITN data from the Nash et al. systematic review[1]. In A-B, the size of points indicates the total number of mosquitoes in the experimental hut data sample for the respective net type. Shaded areas indicate uncertainty around the best fit line.

1.3 Association between experimental hut mortality of different ITNs

Experimental hut trials (EHTs) where pyrethroid-PBO nets (Olyset® Plus, PermaNet® 3.0, Veeralin®, DawaPlus® 3.0, DawaPlus® 4.0), or pyrethroid-pyrrole nets (Interceptor G2® or PermaNet® Dual) were compared in the same trial to pyrethroid-only ITNs were identified from the systematic review of Nash et al. 2021[1]. These data were augmented with unpublished studies conducted by co-authors resulting in 41 comparisons of unwashed ITNs for pyrethroid-PBO ITNs, and 30 comparisons for pyrethroid-pyrrole ITNs in

the experimental hut assay. The predictive relationship between mosquito mortality in huts with pyrethroid-only ITNs, on that of comparator ITNs (pyrethroid-PBO or pyrethroid-pyrrole) was then assessed. The analyses for pyrethroid-PBO and pyrethroid-pyrrole ITNs were conducted separately. Posterior distributions were generated using Stan's No-U-Turn Monte Carlo sampling methods[3].

For each fit generated under the models described, four chains were initialised and the convergence of 2,000 iterations assessed after discarding the first 1,000 of each as burn in. The posterior distribution of parameters were derived from the 4,000 iterations and Bayesian credible intervals were estimated, posterior checks were performed using ShinyStan (version 1.0.0, [4]) and visually confirmed to fit the data.

1.3.1.1 Sampling uncertainty

It is important to highlight there will be inherent sampling uncertainty in the proportion of mortality observed in the N paired EHT studies included. For each study site $i \in \{1, 2, \ldots, N\}$, n_p^i mosquitoes were recorded to have entered pyrethroid-only ($p = 1$) and comparator ITN ($p = 2$) huts. Of these mosquitoes, d_p^i died in study i with net type p. The probabilities of individual mosquito mortality conditional on hut entry, l_p^i, were initially assumed to be constant for all mosquitoes, but unknown, within each study i for each net type p. If independence between studies is also assumed, then posterior estimates of these probabilities can be inferred directly for each site using a standard Bayesian approach. To emphasise when independence between sites is assumed, the notation $\hat{l}_p^i$ is used.

If, for each study i and net type p, non-informative Jeffreys's priors on the probabilities of site-specific mortalities are assumed:

$$\pi(\hat{l}_p^i) \sim \text{Beta}(0.5, 0.5) \qquad \text{(Equation S2)}$$

Then by assuming binomial sampling with fixed site-specific probabilities, this yields independent likelihoods for every i^{th} study for each net type p:

$$\mathcal{L}\left(\hat{l}_p^i \middle| n_p^i, d_p^i\right) \sim \text{Binomial}\left(d_p^i \middle| n_p^i, \hat{l}_p^i\right) \qquad \text{(Equation S3)}$$

and so, by conjugacy, this yields a posteriori:

$$\pi\left(\hat{l}_p^i \middle| n_p^i, d_p^i\right) \sim \text{Beta}\left(0.5 + d_p^i, 0.5 + n_p^i - d_p^i\right) \qquad \text{(Equation S4)}$$

Since $\mathbb{E}\left[\pi\left(\hat{l}_p^i \middle| n_p^i, d_p^i\right)\right] = d_p^i / n_p^i$, the posterior mean estimates of the probabilities of mortality are simply equal to the proportion of dead mosquitoes sampled, while the central 95% credible intervals can be inferred a posteriori from equation S4 (Figure S3).

1.3.1.2 Logistic regression model

The previous estimates for site-specific probabilities of comparator ITN-induced mortality assume independence both between sites and between comparator and pyrethroid-only ITN induced mortality [5]. The uncertainties in these estimates arise from the number of mosquitoes entering both the pyrethroid-only and the comparator ITN huts. However, here we follow Nash et al.[1] and make the simplifying assumption to use the observed proportion of pyrethroid-only ITN induced mortality, l_1, as a predictor for the probability of comparator ITN induced mortality, l_2. This means that uncertainties in the shape of the association arises from the number of mosquitoes entering comparator ITN huts in each site alone. Our rationale for this is due to the data on the number of mosquitoes dying in pyrethroid-only huts being imbalanced; very few EHTs assessing pyrethroid-pyrrole ITNs had a high observed proportion of pyrethroid-only ITN-induced mortality, and those that did systematically recorded fewer mosquitoes entering those huts. By treating the observed proportion of mortality in pyrethroid-only huts as a predictor, this will ensure EHTs with higher pyrethroid-only induced mortality are not under-represented and that pyrethroid-pyrrole do not perform worse than pyrethroid-only ITNs.

Through a Bayesian logistic regression framework, the probability of mortality from comparator nets, l_2^i, is assumed to be dependent on pyrethroid-only net mortality via the link function:

$$l_2^i = \text{logit}^{-1}(\beta_i) = \frac{1}{1+\exp(-\beta_i)} \qquad \text{(Equation S5)}$$

Which has log-odds, β_i, of:

$$\beta_i = \beta_1 + \beta_2 l_1^i \qquad \text{(Equation S6)}$$

Where the proportion of pyrethroid-only morality is calculated directly as $l_1^i = d_1^i / n_1^i$. Assuming binomial sampling with fixed probabilities of mortality from comparator nets for a given pyrethroid-only mortality, the marginal likelihood for site i is:

$$\mathcal{L}\left(l_2^i \middle| \boldsymbol{z}\right) \sim \text{Binomial}\left(d_2^i \middle| n_2^i, l_2^i\right) \qquad \text{(Equation S7)}$$

given the observed data $\boldsymbol{z} \ni z_i = [n_1^i, d_1^i, n_2^i, d_2^i]$. Therefore, under this model, the joint likelihood of this model in full is:

$$\mathcal{L}(\boldsymbol{\theta}|\boldsymbol{z}) = \prod_{i=1}^{N} \mathcal{L}\left(l_2^i \middle| \boldsymbol{z}\right) \qquad \text{(Equation S8)}$$

given the unknown parameters $\boldsymbol{\theta} = [\beta_1, \beta_2]$, for which weakly informative priors were assumed, $\pi(\beta_1), \pi(\beta_1) \sim \text{N}(0, 10^2)$. For each pyrethroid-only mortality value, l_1^j, in a regularly spaced grid, a total of 4,000 samples, $\boldsymbol{l_2^j}$, were generated from the posterior predictive distribution $\pi(l_2|\boldsymbol{z})$:

$$\boldsymbol{l_2^j} = \text{logit}^{-1}\left(\boldsymbol{\beta_1} + \boldsymbol{\beta_2} l_1^j\right) \qquad \text{(Equation S9)}$$

where $\boldsymbol{\beta_1} \in \beta_1$ and $\boldsymbol{\beta_2} \in \beta_2$ are vectors of 4,000 samples from the joint posterior distribution $\pi(\boldsymbol{\theta}|\boldsymbol{z})$. Mean predictive posterior estimates, $\mathbb{E}[\pi(l_2|\boldsymbol{z})]$, and central 95% credible intervals of the probability of comparator ITN induced mortality were calculated over the full range of potential probabilities of pyrethroid-only induced mortality (Figure S3).

There is little indication of an underlying trend from inspecting the residuals, $\left(d_2^i/n_2^i\right) - \mathbb{E}\left[\pi\left(l_2^i \middle| \boldsymbol{z}\right)\right]$; therefore, the assumption of a linear relationship between the explanatory variable (the proportion of pyrethroid-only mortality) and the log-odds appears to be satisfied. However, the credible intervals under this model appear to under-represent the variance in the underlying data. The uncertainty in the predicted conditional probability of comparator ITN mortality may therefore be underestimated.

1.3.1.3 Beta-binomial regression model

Under the previous logistic regression model, the probability of comparator ITN induced mortality was assumed to be constant, but unknown, for a given proportion of pyrethroid-only ITN induced mortality. However, there are likely numerous other covariates that may influence the probability of mortality from comparator nets. Many of these will not be controlled for given the EHTs were conducted across a wide range of locations. Moreover, even within a study, there will likely be individual differences between mosquitoes that affect the relationship between their susceptibility to pyrethroid-only and comparator ITNs.

The data are therefore likely overdispersed in relation to the logistic regression model. To address this, and characterise the full uncertainty in the additional benefit of novel ITNs, a beta-binomial regression model was implemented. Under this model, the probability of comparator ITN induced mortality, l_2^i, conditional on the proportion of pyrethroid-only ITN induced mortality, l_1^i, is no longer assumed to be constant for all individual mosquitoes. Instead, the individual probability of mortality from comparator ITNs is treated as a random variable, such that for site i:

$$l_2^i \sim \text{Beta}\left(\phi \bar{l}_2^i, \phi\left(1 - \bar{l}_2^i\right)\right) \qquad \text{(Equation S10)}$$

Here, ϕ is an overdispersion parameter that aims to capture additional variance arising from other covariates not considered in the model, while the mean probability of mortality from comparator ITNs, $\bar{l}_2^i = \mathbb{E}\left[l_2^i\right]$, is described by the same link function as under the logistic regression model where $l_2^i \equiv \bar{l}_2^i$ was implicitly assumed previously:

$$\bar{l}_2^i = \text{logit}^{-1}\left(\beta_1 + \beta_2 l_1^i\right) \qquad \text{(Equation S11)}$$

Here, β_1 and β_2 are again treated as unknown parameters. The unknown parameters we seek to estimate under the beta-binomial model are therefore $\boldsymbol{\theta} = [\phi, \beta_1, \beta_2]$.

Assuming binomial sampling of a beta-distributed probability of comparator ITN induced mortality, the probability of observing the data conditional on l_2^i gives rise to the marginal likelihood for site i:

$$\mathcal{L}\left(l_2^i \middle| \boldsymbol{z}\right) \sim \int_0^1 \text{Binomial}\left(d_2^i \middle| n_2^i, p\right) \text{Beta}\left(p \middle| \phi \bar{l}_2^i, \phi\left(1 - \bar{l}_2^i\right)\right)\, dp \qquad \text{(Equation S12)}$$

which is equivalent to a beta-binomial distribution:

$$\mathcal{L}\left(l_2^i \middle| \boldsymbol{z}\right) \sim \text{Beta-binomial}\left(d_2^i \middle| n_2^i, \phi \bar{l}_2^i, \phi\left(1 - \bar{l}_2^i\right)\right) \qquad \text{(Equation S13)}$$

The joint likelihood of the beta-binomial model in full is therefore:

$$\mathcal{L}(\boldsymbol{\theta}|\boldsymbol{z}) = \prod_{i=1}^{N} \mathcal{L}(l_2^i|\boldsymbol{z}) \quad \text{(Equation S14)}$$

where the priors on β_1, β_2, remain unchanged from the logistic regression model, a slightly informative log-normal prior on ϕ is assumed to capture our prior belief that the data are likely overdispersed:

$$\pi(\phi) \sim \text{Lognormal}(1, 1.5) \quad \text{(Equation S15)}$$

After fitting the model in Stan, we then generated η =100,000 samples, $\boldsymbol{d_2^j}$, from the posterior predictive distribution $\pi(d_2|\boldsymbol{z})$ through repeated sampling for each pyrethroid-only mortality value, l_1^j, in a regularly-spaced grid:

$$\boldsymbol{d_2^j} \sim \text{Beta-binomial}\left(\eta, \boldsymbol{\phi}\boldsymbol{\bar{l}_2^j}, \boldsymbol{\phi}(1 - \boldsymbol{\bar{l}_2^j})\right) \quad \text{(Equation S16)}$$

where $\boldsymbol{\bar{l}_2^j} = \text{logit}^{-1}(\boldsymbol{\beta_1} + \boldsymbol{\beta_2} l_1^j)$ such that $\boldsymbol{\beta_1} \in \beta_1$, $\boldsymbol{\beta_2} \in \beta_2$ and $\boldsymbol{\phi} \in \phi$ are samples from the joint posterior distribution $\pi(\boldsymbol{\theta}|\boldsymbol{z})$. Posterior predictive samples of the probability of comparator ITN mortality, $\boldsymbol{l_2^j}$, were in turn sampled from the posterior predictive distribution $\pi(l_2|\boldsymbol{z})$ over the same parameter space by calculating:

$$\boldsymbol{l_2^j} = \frac{\boldsymbol{d_2^j}}{\eta} \quad \text{(Equation S17)}$$

This enabled our mean estimates of the probability of mortality for comparator ITNs given the observed proportions of pyrethroid-only ITN mortality when accounting for overdispersion to be derived, in addition to central 95% credible intervals (Figure S3).

1.3.2 Probable outcomes from an *Anopheline* mosquito feeding attempt

When a person is using a net indoors, we consider that any given *Anopheles* mosquito feeding attempt can result in either a mosquito being deterred before entering the hut or those that enter the hut either dying, successfully blood-feeding or exiting unfed. These outcomes are measured using experimental huts in the field as described previously [6,7]. In this work, we use the data from[1], to estimate the probability of *Anophelines* repeating (a combination of deterrence and exiting, r_{pN0}), dying (d_{pN0}) or feeding successfully (s_{pN0}).

The probability that mosquitoes are not deterred by the presence of a net (1 - m_1) is estimated by the ratio of *Anopheles* mosquitoes entering the net hut relative to the control hut without an insecticide treated bed net. It is assumed that this is equivalent for a person with an untreated net or without a net as experimental hut trials commonly use either scenario in their control arms and the data collated for systematic review always supplied untreated nets for control hut volunteers[1]. Using a comprehensive systematic review of experimental huts conducted on mosquito nets[1], the probability of mosquitos entering a hut with an insecticide treated net, regardless of net type, relative to a hut with an untreated net, is defined as:

$$1 - m_1 = 1 - (\delta_1 \exp(\delta_2\{1 - \exp[(1 - l_1)\delta_3]\}/\delta_3)), \quad \text{(Equation S18)}$$

where parameters δ_1, δ_2, and δ_3 determine the shape of the association (Table S2). The probability of mosquitos entering and being caught in control huts is assumed to remain constant, independent of the level of survival in the hut.

The proportion of mosquitoes entering but not blood-feeding (j_p) and entering and successfully feeding (k_p) are associated such that $1 = j_p + k_p + l_p$ (subscript p indicating type of net, be it *1*=pyrethroid-only or *2*=pyrethroid-PBO). The probability of mosquitoes successfully feeding and surviving after entering a hut with an insecticide treated mosquito net (either pyrethroid-only or pyrethroid-PBO) is determined from the systematic review by[1]:

$$k_p = 1 - \exp\left(\theta_1\{1 - \exp\left[\theta_2\left(1 - l_p\right)\right]\}/\theta_2\right). \quad \text{(Equation S19)}$$

Again, parameters θ_1 and θ_2 determine the shape of the association and are shown in Table S2 (Figure S1B).

The estimates for j_p, k_p, and l_p are used to determine the proportion of mosquitoes repeating r_{p0}, successfully blood-feeding s_{p0}, or dying d_{p0} during each feeding attempt in a hut with a net relative to one without a treated net as follows,

$$r_{p0} = \left(1 - \frac{k'_p}{k_0}\right)\left(\frac{j'_p}{j'_p + l'_p}\right), \quad \text{(Equation S20)}$$

$$d_{p0} = \left(1 - \frac{k'_p}{k_0}\right)\left(\frac{l'_p}{j'_p + l'_p}\right), \quad \text{(Equation S21)}$$

$$s_{p0} = \frac{k'_p}{k_0}, \quad \text{(Equation S22)}$$

where $j_p' = m_p j_p + (1 - m_p)$, $k_p' = m_p k_p$, and $l_p' = m_p l_p$ [8]. Historical values are used to estimate k_0 (the mosquitoes that enter a house and successfully feed in the absence of an intervention) [9–11]. Uncertainty is carried through by drawing 50 random posterior predictive estimates from within the 90% credible intervals for each respective parameter as estimated by the statistical fits to generate uncertainty parameter draws for the key transmission model parameters r_{p0}, and d_{p0}. The functional form describing the relationships between successfully blood-feeding s_{p0} mosquitoes and mosquito mortality d_{pN0}, is consistent for all net classes (Figure S1B).

Determination of these statistical associations then enables the mechanisms determining ITN efficacy into the transmission dynamics mathematical model[12] given any level of pyrethroid resistance. Parameter estimates are provided for pyrethroid-only ITNs (Appendix 2), pyrethroid-PBO ITNs (Appendix 3) and pyrethroid-pyrrole ITNs (Appendix 4).

Table S2 Parameter estimates for the analysis of pyrethroid-pyrrole net efficacy showing the mean and 90% credible intervals for parameters.

	Pyrethroid-only nets	Pyrethroid-PBO nets	Pyrethroid-pyrrole nets
Association of susceptibility bioassay mortality and pyrethroid-only net mortality in experimental huts bioassays (Equation S1)			
α_1^2	0.27 (0.15 – 0.40)	-	-
α_2^2	0.86 (0.33 – 2.33)	-	-
Additional benefit of a next-generation net (Equations S10 – S17: beta-binomial fit)			
β_1	-	-1.71 (-1.78 – -1.64)	-0.87 (-0.98 – -0.76)
β_2	-	5.80 (5.55 – 6.05)	5.62 (5.15 – 6.11)
ϕ	-	6.87 (4.48 – 9.81)	5.15 (3.20 – 7.41)
Association between deterrence and mortality in an experimental hut bioassay (all data are fitted together) (Equation S18)			
δ_1	0.41 (0.32 – 0.53)		
δ_2	0.13 (0.00 – 0.66)		
δ_3	1.49 (0.07 – 4.43)		
Association between successful feeding and mortality in an experimental hut bioassay (all data are fitted together) (Equation S19)			
θ_1	4.17 (3.88 – 4.49)		
θ_2	0.02 (0.01 – 0.03)		
Estimated half-life in years of the insecticidal activity of the ITN (Equation S27)			
μ_p	2.662		
ρ_p	-4.056		

1.4 Entomological impact of ITNs

Following Griffin *et al.* [8] it is assumed that the probability of a mosquito biting host *i* during a single attempt to be y_i; the probability that a mosquito bites a host and survives the feeding attempt to be w_i, and the probability of it being repelled without feeding to be z_i. ITNs affect all three of these probabilities as defined in Table S3. For an unprotected individual it is assumed, $y_i = w_i = 1$ and $z_i = 0$. In Table S3, the probabilities φ_I and, φ_B denote feeding on an individual indoors or in bed respectively, r_N denotes the probability that the mosquito is repelled before entering a house given the presence of nets. The corresponding parameter S_N represents the probability of successfully feeding with nets present. These estimates are determined through the systematic review of experimental hut data and are product-dependent given the additional killing effects of pyrethroid-PBO and pyrethroid-pyrrole nets. The repellence is assumed to be at maximum as nets are distributed, r_{p0} (estimated from the systematic review), and decreases to a non-zero estimate of r_{NM} (0.24) following [8].

For all net types, the mortality inducing effect decreases from some maximum on net deployment d_{p0} (estimated using the systematic review). We assume this diminishing effect decreases at constant rate γ_p (estimated from pyrethroid-only nets, see below). At time *t* after nets were distributed, the respective effects are:

$$r_N = (r_{p0} - r_{NM}) \exp(-t\gamma_p) + r_{NM} \quad \text{(Equation S23)}$$

$$d_N = d_{p0} \exp(-t\gamma_p) \quad \text{(Equation S24)}$$

$$s_N = 1 - r_N - d_N. \quad \text{(Equation S25)}$$

Estimating the waning of the insecticide effect and the corresponding integrity of the netting material (together termed γ_p) represents a key limitation for our process currently. To capture the loss of efficacy of an ITN, we define the half-life in years of the killing activity of a pyrethroid-only ITN against a susceptible mosquito population as H_y^S. Following previous work[5] it is assumed the half-life in years of pyrethroid-ITNs in wild mosquitoes (H_y) is proportional to the loss of killing activity seen in 20 washed pyrethroid-only ITNs between wild mosquitoes (H_w) and that observed when the mosquito population was susceptible (H_w^S):

$$H_y = \frac{H_w}{H_w^S H_y^S}. \quad \text{(Equation S26)}$$

We assume that the activity of the insecticide decays at a constant rate $\gamma_p = \log(2)/H_y$, which is related to the ITN survival probability $(1 - l_p)$ via the intercept (μ_p) and gradient (ρ_p) parameters:

$$\text{logit}(\gamma_p) = \mu_p + \rho_p(1 - l_p) \quad \text{(Equation S27)}$$

$$d_{w20} \sim \text{Binomial}(N_{w20}, \gamma_p)$$

Where d_{w20} is the count of mosquitoes that are killed when nets that have been washed 20 times are tested in the EHT, out of a total N_{w20} mosquitoes. The code for statistical models outlined in section 1.1 are available at https://github.com/EllieSherrardSmith/Mosq-Net-Efficacy.

Table S3 (repeated from [8]. Probabilities of a blood-feeding mosquito successful feeding, biting and being repelled (leaving without feeding or dying) in the presence of insecticide treated nets.

Probability of successful feeding (w_i)	$1 - \Phi_B + \Phi_B s_N$
Probability of biting (y_i)	$1 - \Phi_B + \Phi_B s_N$
Probability of repellency (z_i)	$\Phi_B r_N$

For a full description of the transmission model, see Sherrard-Smith et al.[13]. The transmission model code is freely available (GitHub - mrc-ide/malariasimulation: The malaria model). Explicit assumptions relevant to our analyses in this contribution are outlined in the following section.

1.5 Recreation of cluster randomised control trials

1.5.1 Data availability

Models were parameterised using baseline data from cluster randomised control trial (CRT) from Tanzania and Benin[14–17]. Per protocol estimates of malaria prevalence and for the different arms of the trial are compared to model predictions for the three years following mass ITN distribution. Empirical data are plotted against model projections of the same age range (which varied between study) in Figure 2A and B of the main text. Note that in Tanzania the last survey at 36 month was omitted from the figure as it was conducted between 12th January and 10th February 2022 and in all arms children in grades 1, 3, 5, and 7 attending primary schools received a pyrethroid-PBO in October 2021[15] substantially changing the mix of ITNs in the community so comparisons between arms are no-longer valid.

1.5.2 Ethical approval

No specific ethical approvals were required for this secondary modelling analysis though both previously published studies received their own ethical clearance. In Tanzania study ethical approval was granted by Institutional review boards of the Tanzanian National Institute for Medical Research (reference NIMR/HQ/R.8a/Vol.IX/2743), Kilimanjaro Christian Medical University College (2267), London School of Hygiene & Tropical Medicine (14952), and University of Ottawa (H-05-19-4411). Written informed consent was obtained from an adult guardian for selected children and from any adult living in households selected for mosquito collection. In Benin ethical approval was obtained from the Benin Ministry of Health ethics committee (6/30/MS/DC/SGM/DRFMT/ CNERS/SA), the London School of Hygiene & Tropical Medicine ethics committee (16237), and the WHO Research Ethics Review Committee (ERC.0003153). Written informed consent was obtained from all participants, or from guardians for participants younger than 18 years.

1.5.3 Insecticide-treated net (ITN) coverage

ITN use wanes over time since distribution due to net material quality and integrity, seasonal patterns in humidity or other social patterns of use [18–20]. This waning in use of trial nets was observed throughout the trial in each trial arm, yet the overall net use remained high, with trial nets being replaced with pyrethroid-only ITNs irrespective of trial arm[17]. This switching to the same net type diminishes the difference between arms and causes the epidemiological impact of better nets to be underestimated. In the transmission model, we need to account for both these observations. To do this, for the trial arms we first fit an exponential decay function to the trial net trends to track the reduction of these nets, we then simulated the model such that any lost trial nets are replaced with aged pyrethroid-only mosquito nets to maintain overall cover of the community.

To do so, the proportion of people using ITNs the previous night (henceforth coverage) was sampled on different months post distribution during the randomised control trials (RCTs) in Tanzania[17] and Benin[16]. In both these trials the net type used was also identified. Following the observed trends in coverage, we assumed that bed net use remained constant at the mean sampled value of any type of ITN (excluding at baseline) (λ)

(dashed line; Figure S2). To simulate the decline in trial ITN coverage that was observed in each trial arm, i, we followed the methods described in Griffin et al. 2010 [8]: it was assumed that the time until a person stops sleeping under a net is exponentially distributed, and coverage declines according to the constant rate, μ_i,

$$\mu_i = 1 - e^{-1/r_i}, \qquad \text{(Equation S28)}$$

where the r_i parameter determines the retention time for trial arm i. The coverage (C) at time t days post ITN distribution is,

$$C_i(t) = \zeta_i e^{-\mu_i t}, \qquad \text{(Equation S29)}$$

given the initial coverage ζ_i. The r parameter was fitted to the numbers of people using trial nets at different times post intervention for each trial arm independently, assuming a binomial likelihood:

$$x_{i,t} \sim \text{Binomial}\left(m_{i,t}, C_i(t)\right), \qquad \text{(Equation S30)}$$

where $x_{i,t}$ is the number of people sleeping under a trial net in trial arm i at time t, and $m_{i,t}$ was the corresponding number of people sampled. The model was fitted in a Bayesian framework using the Stan No U-turn Markov chain Monte Carlo sampler (RStan version 2.32.3[3]) with four chains each with 2500 iterations inclusive of 1250 warm up iterations [21]. A weakly informative prior of $r \sim \text{Gamma}(\text{shape} = 10, \text{ rate} = 4)$, was used, meaning the prior mean retention time was 2.5 years, which is similar to the retention time assumed by the WHO (3 years) and the recently estimated overall median values across sub-Saharan Africa (1.64 years) [22]. The r parameter for trial nets was fitted assuming ζ_i was fixed at the value of λ_i independently for each trial arm i. The modelled declines in trial net use for each trial arm is shown in Figure S2.

To implement a constant coverage of any net type at the value of λ and allow the coverage of trial nets to decline within the existing malaria transmission model framework [23], we simulated the model with a very long r parameter (>25 million years) and replaced the trial nets with aged nets each 30 days, such that the proportion of trial nets declined in a stepwise manner (stepped, black line; Figure S2). The coverage of trial nets was determined by the mean value of C_i between 30 day intervals: $\frac{\frac{1}{r_i}e^{-(r_i(t))} - \frac{1}{r_i}e^{-(r_i(t+30))}}{30}$. To implement these coverages into the model all nets were replaced with pyrethroid-only nets at each time the day prior to trial net implementation, meaning the mean trial net coverage was increased to account for the day during which there were pyrethroid-only ITNs. All nets were assumed to have aged according to the equations described in Griffin et al [8], given the pyrethroid-only net aging parameters, meaning nets were replaced with nets that were of the same age (i.e. if 1-year into the trial someone stops using a pyrethroid-pyrrole net then it is assumed that they start using a 1-year-old pyrethroid-only net). This assumption is necessary as the durability of the replacement

nets from the trials were not recorded. However, by assuming trial nets are replaced by aged pyrethroid-only ITNs the overall epidemiological impact will be less than if nets were replaced with better quality newer nets. Like trial conditions, the routine distribution of ITNs though other channels such as schools is excluded.

The malaria transmission model was run to equilibrium prior to the most recent mass distribution of ITNs, assuming the use of pyrethroid-only ITNs was at baseline coverage (prior to the mass distribution) and that all ITNs were 3 years old (i.e., ITN mass distribution campaigns prior to the beginning of the trials were simulated in 2015 in Tanzania and 2017 in Benin). For the mass distribution campaigns, existing ITNs were instantly replaced with new nets of that trial arm at the beginning of the year.

Counterfactual simulations were run where (simulation 1) trial nets were replaced with trial nets, meaning the coverage of trial nets did not decline (and all trial nets were of the same age). We also ran counterfactual simulations in which the (simulation 2) coverage of trial nets declined (i.e., the model was simulated using fitted r parameter) and nets were not replaced, and (simulation 3) the coverage of existing nets declined and there was no mass distribution of trial nets at the beginning of the RCT. For counterfactual simulations 2 and 3 to maintain a high coverage of nets at baseline aged nets were added to top up the coverage to the λ_i value every 30 days.

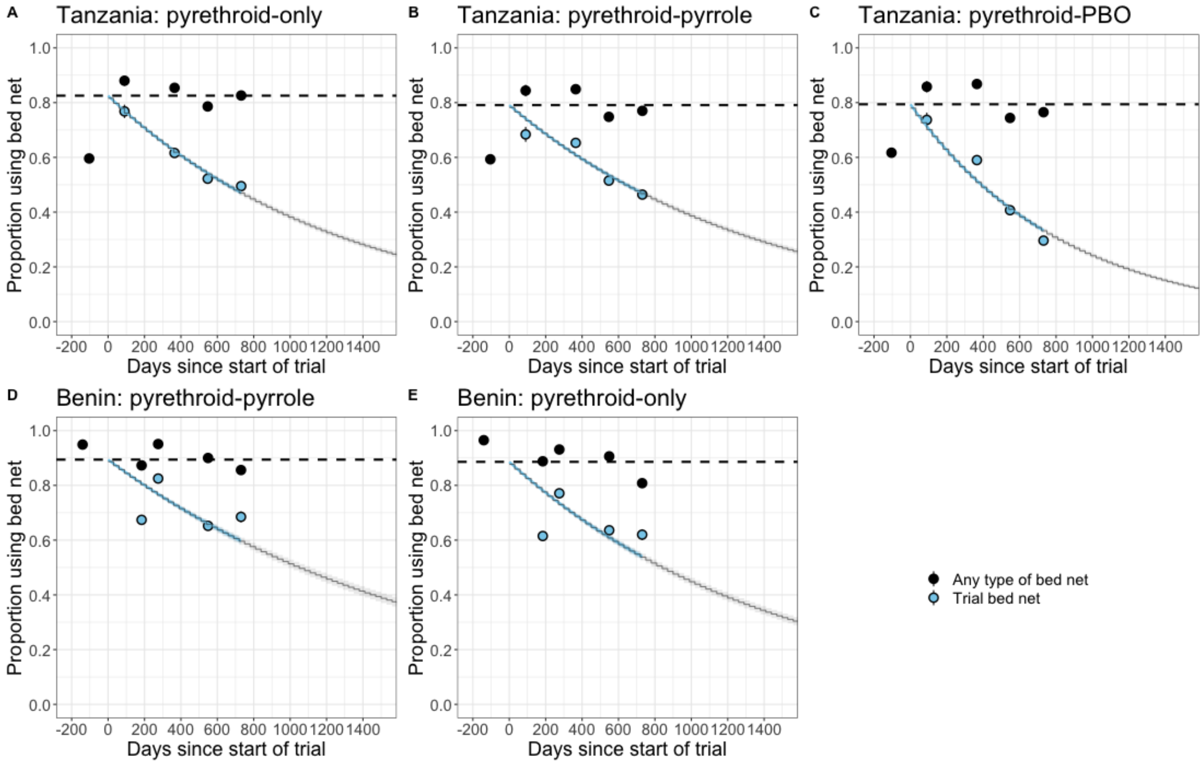

Figure S2 The decline in long-lasting insecticidal net (ITN) use during cluster-randomised control trials (CRTs) of pyrethroid-pyrrole ITNs in Tanzania and Benin. A – E shows the net use in each trial arm. For each trial arm, the proportion of people using any type of bed net is shown in black, and the proportion of people using the trial net is shown in blue. Points show the raw data for each trial arm reported in the respective CRTs[14–17]. The dashed black line shows the weighted mean proportion of people using any type of bed net (including at baseline; weighted by the number of people sampled at each time). The blue line shows the fitted decline in the proportion of people using the trial net (95% credible intervals are shaded).

1.5.4 Model simulations

The mosquito model is deterministic and tracks larval and adult mosquito population and infection dynamics, given differences in vector control. The adult mosquito model assumes that (i) mosquito life expectancy is exponentially distributed and (ii) the extrinsic incubation period (EIP) is fixed at 10 days. The transmission model was run with three different species of malaria-transmitting mosquito: *Anopheles gambiae sensu-stricto*-like, *Anopheles coluzzi*-like and *Anopheles funestus*-like. For the Tanzania trial mosquitoes were identified as *A. funestus* or not, and all other mosquitoes were assumed to be *A. gambiae s. s.*, the proportions of mosquitoes of each species at baseline is published and we assumed these values remained constant for the duration of the trial[17]. For the Benin trial the numbers of mosquitoes caught for each species were available over the duration of the trial[16], so we assumed the overall sampled values were constant for the duration of the simulation. In Benin, a small proportion of the sampled mosquitoes were identified as *Anopheles gambiae sensu lato*, and these were assumed to be either *A. coluzzi* or *A. gambiae s. s.* with equal probability. *A. gambiae s. s.* and *A. coluzzi* were assumed to have the same parameter values. The relative proportion of the different mosquito species and their bionomic parameter values are given in Table S4.

Table S4 Relative proportion of different mosquitoes in the Tanzanian and Benin cluster-randomised control trials (CRTs) and assumed mosquito bionomics. The relative abundance of the mosquito species and the level of pyrethroid resistance (indicated by the percentage mortality in a pyrethroid discriminating dose bioassay) were estimated for each arm, with other values being estimated at the trial-level or being universal across all trials. The different trial arms are labelled according to the ITN distributed, be it pyrethroid-only (pyr-only), pyrethroid-PBO (pyr-PBO) or pyrethroid-pyrrole (pyr-pyrrole). The rate in days that mosquitoes seek a blood meal, the time spent foraging for that blood meal, the proportion of blood meals taken on humans (human blood index, HBI), the proportion of bites taken when people are in bed or indoors were all measured in the absence of mosquito control. The relative abundance of different mosquito species and their level of pyrethroid resistance were measured in sentinel clusters within the different trial arms. Unless otherwise stated other parameter estimates were taken from [8,24–26]. Country and species-specific estimates of the proportion of bites in bed and the proportion of bites indoors were obtained from Sherrard-Smith et al. [27] For the pyrethroid-pyrrole RCT in Benin the discriminating dose bioassay mortality estimates were calculated as the mean over two years.

**Parameter**	***A. gambiae sensu stricto*-like**					***A. coluzzi*-like**					***A. funestus*-like**				
Country	**Tanzania**			**Benin**		**Tanzania**			**Benin**		**Tanzania**			**Benin**	
Trial Arm	Pyr-only	Pyr-PBO	Pyr-pyrrole	Pyr-only	Pyr-pyrrole	Pyr-only	Pyr-PBO	Pyr-pyrrole	Pyr-only	Pyr-pyrrole	Pyr-only	Pyr-PBO	Pyr-pyrrole	Pyr-only	Pyr-pyrrole
Proportion of the mosquito population	0.06	0.07	0.05	0.26	0.26	0	0	0	0.74	0.74	0.94	0.93	0.95	0	0
Bioassay mortality %	33	42	33	4.8	4.6	33	42	33	4.8	4.6	33	42	33		
Blood feeding rate per day	1/3														
Time (days) spent foraging	0.69														
Human blood index (HBI) ǂ	0.92										0.94				
Proportion bites taken when people in bed	0.76			0.86					0.86		0.68			0.70	
Proportion bites taken when people indoors	0.85			0.91					0.91		0.75			0.81	
Background death rate per day	0.132										0.112				

The impact of different ITNs on the probability that a mosquito is repelled, killed or successfully blood feeds have been estimated from EHT data given differences in pyrethroid resistance (as measured using discriminating dose bioassays) for pyrethroid-only and pyrethroid-PBO ITNs[1]. These parameter estimates have been validated against published RCTs[27]. In this study, we applied the same process to these data for pyrethroid-pyrrole nets. We assumed the estimates for pyrethroid resistance measured in each trial arm at the start of the trial – as approximated by survival in the WHO discriminating dose bioassay – were constant throughout the simulations, meaning we do not account for temporal changes in pyrethroid resistance during the trial. In Benin, estimates of pyrethroid-resistance for each trial arm were provided for two years and we calculated the discriminating dose bioassay mortality as the mean of these values weighted according to the number of mosquitoes tested each year (mean values are provided in Table S4). Underlying mosquito abundance was assumed to remain consistent. The estimated half-life of insecticide active on nets was assumed to be equivalent between the net types in the absence of durability data for newer nets. The model was simulated with the median, 95% upper and lower ITN parameter (probability of ITN repellence, probability of ITN mortality, retention and ITN half-life parameters) credible interval values were used respectively. The model is stochastic and was run three times for each unique combination of trial arm and ITN efficacy uncertainty (median and 95% credible intervals).

Assumptions on clinical treatment can impact on the estimated cases as simulated in the transmission model. In the model, artemisinin-combination therapies (ACT), like Artemether-lumefantrine, are assumed to eliminate gametocytes as well as providing prophylactic protection for about a month. Non-ACT treatments provide slightly lower protection and do not eliminate gametocytes so these patients can potentially pass on infection to mosquitoes though at lower probability. The Tanzania trial team provided information on the access to care with antimalarial drugs treatment for patients presenting with clinical infection, but this information was not available for the Benin trial and so for simplicity we assumed the most recent coverages of ACT and non-ACT from the R foresite data package (https://mrc-ide.github.io/malariaverse/). All values were assumed to remain constant at the values given for rural areas at the most recent available time (2022). For the trial arms in Benin the coverage values from the Zou department administration unit were used: 16% ACT coverage and 27% non-ACT coverage. For the trial arms in Tanzania the values from the Mwanza administrative region were used: 50% ACT coverage and 6% non-ACT coverage.

The vector model used is compartmental and described in White et al [28]. Mosquitoes emerge from eggs and progress through larvae and pupal stages regulated by density-dependent mortality. A time-varying mosquito carrying-capacity is used to simulate the availability of suitable breeding sites. The baseline transmission intensity is regulated by this carrying capacity as it determines mosquito numbers in the absence of interventions. Only female mosquitoes are tracked as adults assuming the sex ratio is 1:1. Female mosquitoes emerge into the susceptible state and are assumed to become infected at a rate determined by the infectiousness of the human population given a time lag to simulate the extrinsic incubation period. The force of infection from humans to mosquitoes is assumed to be dependent on all human infected states.

1.5.5 *Anopheles* mosquito bionomics

The ecology of the mosquito vector determines the effectiveness of indoor vector control interventions. Table S4 shows the default parameters assumed in the modelling exercises given a series of systematic reviews of the literature [8,24–26].

1.5.6 Seasonality in transmission

Seasonality is incorporated in the model by allowing a time-varying carrying capacity that depends on the mean rainfall over the year as estimated from rainfall data spanning 2016-2019 following [29,30] and updated in 2021[12]. We do not specifically match rainfall patterns through the trial and estimates of all trial arms are identical, reflecting the rainfall measured at the administrative district 1 scale. Changes in temperature are not considered.

1.5.7 Model calibration

The model was calibrated singularly for each trial arm by varying the mosquito density per person, such that the modelled malaria prevalence at the baseline of the trial matched the equivalent sampled values of malaria prevalence (as determined by rapid diagnostic test) in the defined age group given the existing use of ITNs. Note that the age group sampled varied between trials. This process was conducted using the R Cali package[31], which implements an iterative root finding algorithm. The model was calibrated using the posterior median parameter values for the probability that a blood feeding mosquito will repeating (r_{pN0}), die (d_{pN0}) and the ITN half-life given the corresponding discriminating dose bioassay mortality estimate of each trial arm. Uncertainty in model predictions are provided by repeating simulations with different posterior samples from the three ITN efficacy parameters but keeping all other values the same. Ninety-five percent credible intervals are plotted, though because only a small subset of model uncertainty is included, these values should be treated with caution.

1.5.8 Reproducible code

The statistical steps outlined above and the code used in the fitting process for estimating the mosquito net efficacies are freely available at https://github.com/EllieSherrardSmith/Mosq-Net-Efficacy. Uncertainty draws for *malariasimulation* model parameters can be generated using “R code/Part 6 Functions to output parameters with uncertainty.R” in the *Mosq-Net-Efficacy* repository The validations for the CRTs are provided here: https://github.com/IsaacStopard/RCT_prediction. The transmission model is run through the *malariasimulation* framework[12] and freely available (https://github.com/mrc-ide/malariasimulation/tree/master/R)

1.5.9 MINT simulations

With an aim to represent a range of possible settings across malaria-endemic countries, MINT simulations were designed to offer a choice of some key variables that can impact the effectiveness of vector control interventions. Care should be taken when considering the geographic scale of the area under investigation and decisions should be made according to the question under investigation (for example, IRS is implemented at a

more local level), heterogeneity in key metrics (how much a site varies in entomological and epidemiological terms) and importantly the quality of data used to parameterise the model. Further details are provided in the User Guide (Appendix 5) or online (https://mint.dide.ic.ac.uk/public/resources/MINT%20v2.0%20User-Guide-English.pdf).

MINT is parameterised to explore malaria in two broad seasonality settings, one where transmission is perennial throughout the year, and one with a single peak in transmission per annum. The choice of either defines the mosquito density patterns throughout the simulation, in turn these drive transmission patterns that lag about 35 days relative to peaks in mosquito vector numbers. Previous work [13] has shown us that endemicity is a critical parameter to define to allow the simulations to represent the impact from interventions. Seven different microscopy-tested prevalence levels for children under 5 years of age are offered for selection. This estimate should align with year 0 in the presented figure once all information is entered. The simulation is calibrated to match this specified level of burden. Mosquitoes may show different propensities to bite people when indoors, so MINT offers either a relatively high probability (~97% bites taken when people are indoors) or low option (~78% bites taken when people are indoors). Similarly, anthropogenicity among mosquitoes can vary, MINT currently enables high human biting (~92% of mosquito bites take blood feeds on humans) or low human biting (~74% of all bites taken on humans). Given MINT is explicitly focused on vector control and pyrethroid resistance is proving both a critical challenge and decision maker for the use of different ITNs, MINT includes 6 different profiles for pyrethroid resistance in the local mosquitoes. The higher this estimate, the less effective the ITN becomes in all cases, as all include some pyrethroid as an active ingredient. Preliminary work also demonstrates that the level of historic interventions is important to estimate the potential of future efforts. For example, if high control is in place, it is tougher to reduce burden for any community in comparison to a location with little control ongoing. Therefore, MINT simulates different levels of ITN use and IRS presence historically.

These baseline simulations are calibrated to the defined prevalence in children under 5 years as stipulated by the user before the next vector control is implemented. Model simulations are run which assume the user defined population use of baseline interventions (ITNs and IRS) whose efficacy is determined by the user defined mosquito bionomics (preference for biting indoors, preference for biting people, and the level of pyrethroid resistance). Different calibrations are therefore needed for each of the combinations of the seven variables the user can define (excluding the population at risk, which doesn't change estimates of epidemiological efficacy). The mosquito-to-human ratio is varied so that malaria prevalence matches that defined by the user utilising the R Cali package [31], which implements an iterative root finding algorithm. Going forward from time 0, MINT includes 10 different use-levels for ITNs, and 6 different use-levels for IRS. These interventions can be simulated alone or in combination. All simulations are pre-run and stored outcomes are summarised allowing the tool to call the relevant set depending on the user choices.

IRS is a WHO recommended intervention that can be used in isolation or to complement ITNs. In the transmission model simulations for MINT, we use parameter estimates for a long-lasting product matching those identified for pirimiphos methyl IRS in Sherrard-Smith et al. (2018) [32]. This work performed a meta-analysis of

experimental hut trials on IRS products to identify the induced mortality, repellence and successful feeding probable in the presence of this intervention type (https://github.com/EllieSherrardSmith/Mosquito-Spray-Parameters). IRS is assumed to be deployed overnight to the proportion of the total population defined by the user in MINT. Delivery of IRS, in reality, may be spread out across recipient households over a few weeks or months. In MINT, IRS is delivered annually for each of the 3-years shown in the simulation. In the current version, we do not have any resistance in mosquito populations to organophosphate or neonicotinoid insecticides, the principle actives currently used in IRS. The mosquito bionomics specified (the proportion of mosquitoes feeding indoors or in bed, the proportion feeding on people and the feeding rates and life expectancy) interact with ITN and IRS efficacy so that there are subtle differences in the mosquito outcomes. For both ITNs and IRS, entomological impacts are assumed to be independent of the mosquito species and determined by the user-defined bionomics and the level of pyrethroid resistance. This is because the differences within species can be greater than that seen between species, and there is also insufficient data to differentiate the entomological impact of ITNs and IRS between species. Instead, users are requested to select values that represent the overall vector population in their site of interest. In the simulations shown in MINT we have 50% *Anopheles gambiae* s.s.-type vectors, and 25% of both *An. funestus* s.s.-type and *An. arabiensis*-type vectors. The differences in bionomics between these mosquitoes is shown in Table S4.

MINT asks the user to describe the general mosquito behaviour in the setting, giving options for either low or high indoor or human biting, and an estimated pyrethroid resistance profile for mosquitoes (the proportion surviving in a discriminating dose bioassay for pyrethroid ITN efficacy). This might limit the accuracy of simulations if there are substantial local differences in mosquito bionomics in the setting. MINT is currently unable to reflect any changes in mosquito behaviour over the simulation, so shifting levels of pyrethroid resistance or species abundance through time are not reflected in results. Results are restricted to 3 years.

The MINT tool can be used to stratify the use of intervention combinations across multiple settings. We advise careful interpretation for any economic analyses, as the most impactful interventions (one that reduces cases or prevalence most powerfully) might not be most cost-effective, and always, the goals of NMPs should be prioritised. The online tool is flexible enough to explore multiple options however, and different data resources as preferred by NMP teams could be used to parameterise the MINT tool inputs for the user. In this way we anticipate MINT being a useful explorative tool for thinking through potential options. We recognise that data collection can be challenging, and that different sources may be favoured by each NMP team. The flexibility of the MINT interface could allow different resources to be considered, and we suggest trialling ranges for parameters where uncertainty exists.

This approach has multiple limitations. Substantial uncertainty exists on the entomological effect of interventions; the discriminatory dose susceptibility bioassay data is notably variable [1,33–35], experimental hut data contains unexplained variability day-to-day [36] and cluster level data from RCTs, that are used in our process to validate parameterisations, can also contain uncertainty[27]. The discriminating dose assay is considered relatively poor at differentiating between moderate and highly resistant mosquito populations given the scenario where most mosquitoes survive but may be unfit to transmit. This may translate as an intervention impact that is

more or less impeded by mosquitoes that are generically classified as resistant. Another important limitation to acknowledge is that uncertainty exists about how the next generation ITNs might age under field conditions, making durability challenging to represent within the mechanistic model. We do not consider sub-lethal effects[37]. Additional bioassays may be able to reduce the uncertainty in model predictions and appreciate the full potential of novel ITNs in model simulations. IRS simulations are restricted to long-lasting products as variability of effects from different products are within a similar range.

This work does not consider mosquitoes developing resistance to chlorfenapyr (nor to pirimiphos methyl or chlothianidin that are used for longer-lasting IRS) which is likely to occur in the absence of good resistance management practices. Those using MINT in the future should verify there has been no loss of susceptibility in the pyrrole insecticide for results to be considered. To robustly manage resistance, we would need to rotate products that have potentially different costs. We do not account for this within the MINT tool currently. The transmission model that we use does not predict selection rates for resistance to any class of insecticide, or synergist, nor the evolutionary lifespan of a chemical class. Consequently, we also advise MINT users considering integrated vector management when making decisions on vector control deployment strategies. Another limitation is that we assume ITN usage to be equivalent between the different ITN classes; however, the effective usable life-length of an ITN depends on physical integrity and durability of the active ingredient. Differences between brands likely exist and these will impact on adherence to use. We have not yet considered alternative distribution channels to mass ITN campaigns. Such implementation pathways may offer alternative cost-effectiveness options [38–40]. A simple economic analysis is included within MINT, this needs to be extended to provide greater detail of uncertainty and the influential factors for vector control procurement and delivery.

1.5.10 Comparison of MINT outputs and bespoke simulations

The MINT interface restricts options for parameter estimates. There are two steps required for users to have confidence in outputs presented in MINT. The first step is to determine whether the underlying transmission model can reasonably recreate the changes observed in empirical epidemiological outcomes from gold-standard CRTs (as we have demonstrated for all the interventions that are included in MINT in [27] and this manuscript). The second step must explore how well MINT simulations recreates this best effort to simulate empirical data, given restrictions on the range of parameter settings available. Here, we demonstrate the use of MINT for the trials reported above. Table S5 shows the parameters chosen for the MINT model simulations, and Figure S10 compares the mean model simulation for the bespoke trial validations to those that are presented using MINT. MINT presents outcomes for children under 5 years of age because this cohort are generally represented within Health Facility data, and Demographic Health Survey Data that may be used by decision makers to determine vector control strategies. The transmission model is able to output any age cohort, so the information present in Figure S10 is distinct to the validation plots because it shows the matched age cohort for MINT. Given that the pyrethroid resistance profile of the Benin trial lies between two choices within MINT, for this trial we show outcomes for both.

In this case, MINT outputs are reasonably similar to the bespoke modelling outputs. The specific differences are that the bespoke model was able to simulate more closely the retention of nets by community members, and the

top-up or replacement of ITNs when trial nets were no longer used. Second, the simulated timing of the ITN distributions is slightly shifted in the MINT simulations; in Figure S10 we show that MINT simulations distribute new nets in January, while the bespoke modelling exercise could match the respective timing of the trials. Another major difference observed is the seasonal profile assumed; whereas the bespoke effort could simulate the respective trial sites, MINT is restricted to showing perennial or single peak per annum seasonality. Nevertheless, MINT simulations still give us an appropriate inference on potential comparable effectiveness of the three ITN classes under investigation.

Table S5. Comparing model parameters used in bespoke trial simulations and closest MINT outputs. Table shows a selection of parameters that were used for the comparison between bespoke simulations and MINT outputs in section 1.2.10 of this appendix and Figure S10. For each parameter that can be varied in MINT, the closest MINT output was found for each trial arm. *For the Benin trial, two resistance levels were chosen for the MINT outputs as the bespoke simulation values lay between these. Both seasonal and perennial MINT outputs were extracted, as shown in Figure S10, and only MINT outputs with no historical or future indoor residual spraying coverage were extracted. ITN = insecticide treated net.

Country	Arm	Baseline prevalence (%)		Baseline ITN use (%)		Initial ITN use post-distribution (%)		Pyrethroid resistance (%)		Mosquito biting location			Anthropophagy	
		**Bespoke**	**MINT**	**Bespoke**	**MINT**	**Bespoke**	**MINT**	**Bespoke**	**MINT**	**Bespoke (indoor)**	**Bespoke (in bed)**	**MINT (indoor in bed)**	**Bespoke**	**MINT**
Tanzania	Pyrethroid-only ITN	45.9	50	59.6	60	82.5	80	67.0	60	0.76	0.69	Low	0.94	High
Tanzania	Pyrethroid-PBO ITN	42.0	40	61.7	60	79.4	80	58.0	60	0.76	0.69	Low	0.94	High
Tanzania	Pyrethroid-pyrrole ITN	42.7	40	59.3	60	79.1	80	67.0	60	0.76	0.68	Low	0.94	High
Benin	Pyrethroid-only ITN	46.5	50	96.5	80	88.6	90	90.0	80/100*	0.91	0.86	High	0.92	High
Benin	Pyrethroid-pyrrole ITN	40.7	40	94.9	80	89.4	90	94.2	80/100*	0.91	0.86	High	0.92	High

1.5.11 Cost effectiveness

Within MINT Version 2, delivery and product costs are entered by the user. The number of cases averted is calculated by taking the case estimates for an intervention 'I' across 3 years (E_I) relative to a scenario where no intervention is implemented (Output 1, denoted E_0) and multiplying by the population size (N) determined by the user,

$$\text{Number of cases averted per population per campaign } (A) = (E_0 - E_I)N \qquad \text{(Equation S31)}$$

This is converted to incidence rates per person, or per 1000 people.

Mass campaigns are assumed to be implemented every 3 years for ITNs regardless of net type. Within MINT the user can enter a procurement buffer (P_B) that is applied by countries estimating the number of nets required to cover their population. Countries also plan using assumptions on the number of people per net (procurement target, P_T) which is entered by the user with a default set to 1.8 people per net. Given the population size the total number of nets required (P) for universal coverage is calculated.

$$\text{Number of nets purchased } (P) = P_B N/P_T \qquad \text{(Equation S32)}$$

It is assumed there is no cap on the number of nets delivered per household and that is equivalently costed to move from e.g. 2 people per net to 1.8, or 1.8 to 1.6. This assumption is generally non-linear [41] and local data can be used to refine these estimates. The routine distribution of nets through local health systems (such as through the extended programme of immunisation or antenatal clinics) are not currently considered.

To very simply evaluate the cost of the mass campaign ($Cost_p$), we use the purchase cost for each net type (subscript, i) given the inputted cost of net purchase price (per net, denoted ITN_{Di}) and the mass distribution campaign delivery cost per person (ITN_P).

$$Cost_P = (ITN_{Di} + ITN_P)P \qquad \text{(Equation S33)}$$

The cost per case averted is then simply, $Cost_p / A$.

1.5.12 Strategising across regions

Resources are restricted for vector control. While we recommend caution given the MINT Version 2 scenarios represent an assessment of mean impacts, we anticipate the strategic section of the tool to serve some value in supporting countries with challenging decisions on distributing interventions in a cost-effective manner.

2 Supplementary Figures

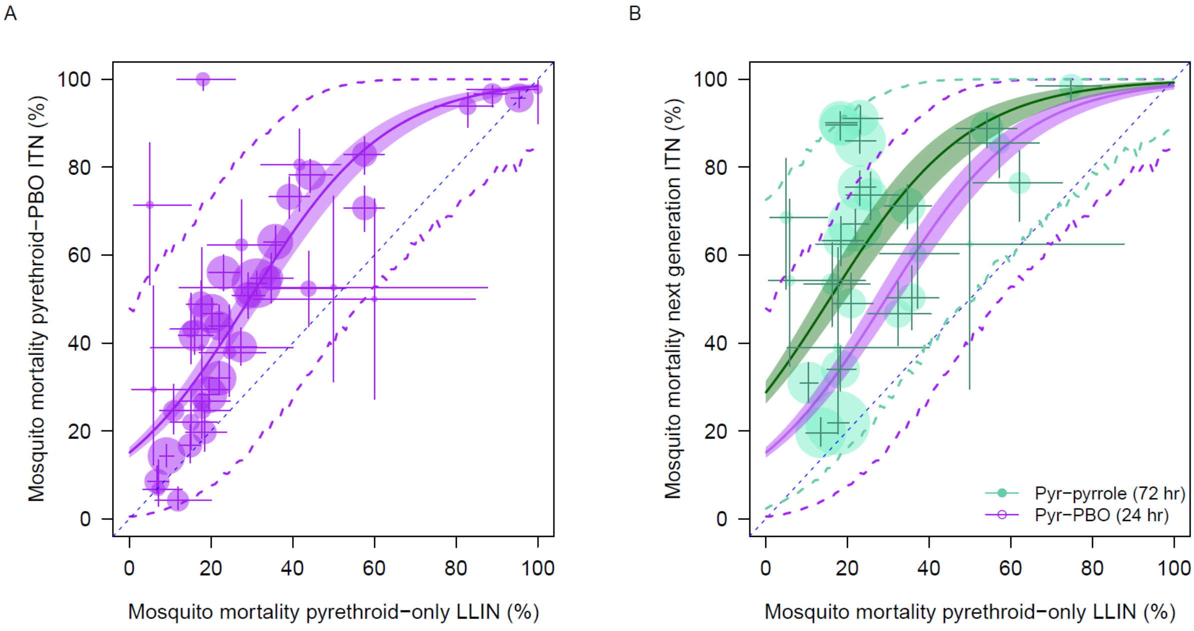

Figure S3. The association between mortality induced by different types of ITNs. Each point indicates the posterior mean estimates and 95% credible intervals (horizontal and vertical lines) of the probability of mosquito mortality when assessed independently for each experimental hut trial. Points are sized proportionally to the number of mosquitoes that entered novel ITN huts. The predictive posterior mean probability of comparator ITN induced mortality under both the logistic and beta-binomial models are shown by the solid curved lines. Central 95% credible intervals are shown for the individual (region bounded by dashed lines) and mean (shaded region) probability of mortality under the beta-binomial model. The credible intervals around the mean under the beta-binomial model are also equivalent to those for the probability of individual mosquito mortality under the logistic regression model. (A) The advantage of pyrethroid-PBO nets over pyrethroid-only nets is seen by comparing 24-hour mortality in experimental huts. (B) The comparison of the benefit from next generation nets either pyrethroid-PBO (purple) or pyrethroid-pyrrole (green) nets. Note that for the pyrethroid-pyrrole ITNs comparison 72-hour mortality is used for both pyrethroid-only and pyrethroid-pyrrole ITNs, so individual green and purple points are not directly comparible. The number of studies directly comparing pyrethroid-PBO and pyrethroid-pyrrole ITNs is relatively low (8 out of 96 - 12%), so direct comparisons in a network meta-analysis was not undertaken, though should be considered as more sites evaluate multiple ITNs.

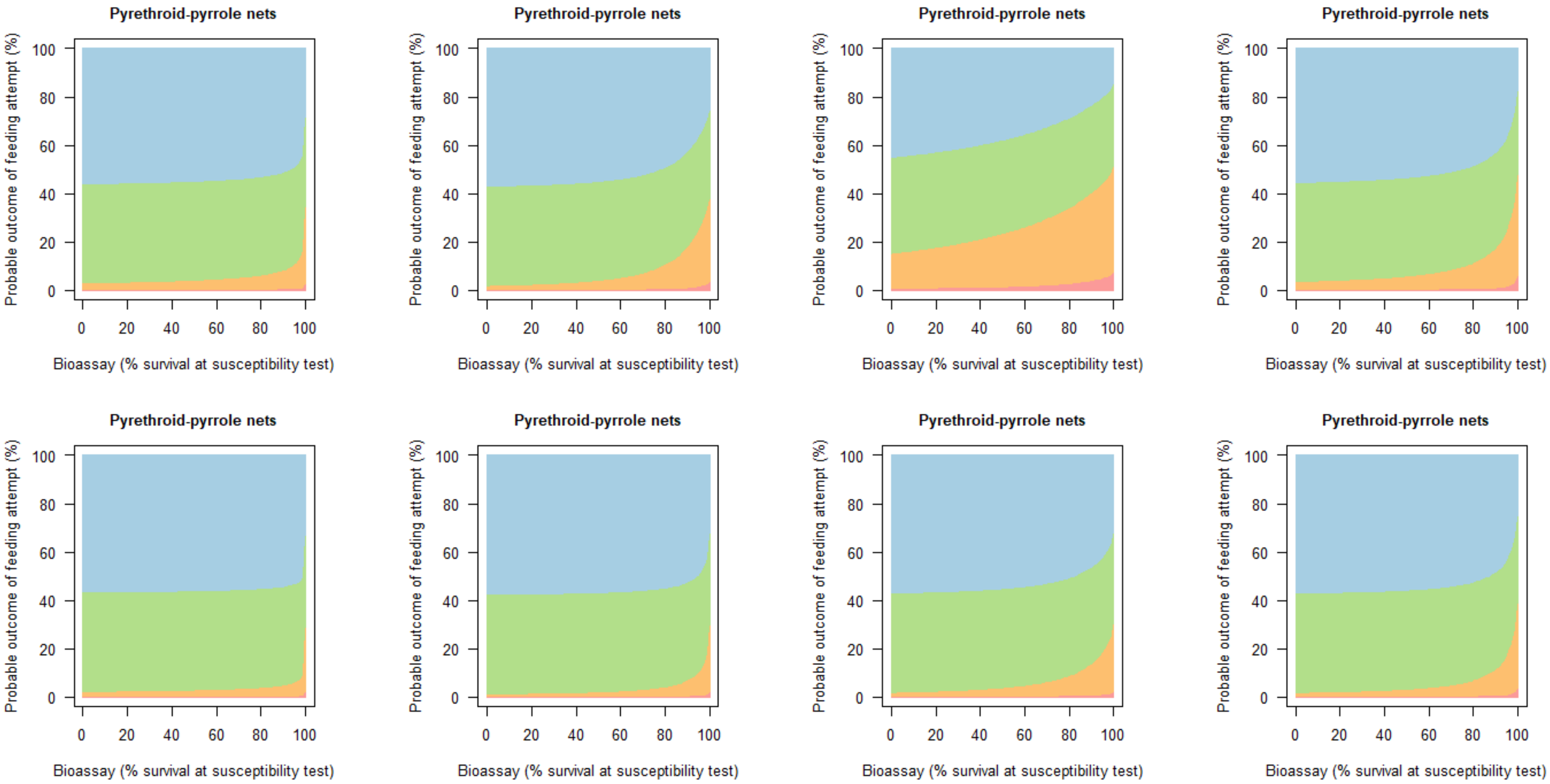

Figure S4. Uncertainty estimates for the probability plots for the outcome of a mosquito feeding attempt in the presence of pyrethroid-pyrrole ITNs. Each iteration of the figure is constructed from the posterior-distributions of the fitting process and illustrates the uncertainty in ITN parameters. Mean estimates are shown in Figure 1B of the main text. This uncertainty is used within the epidemiological predictions shown in Figure 2 of the main text.

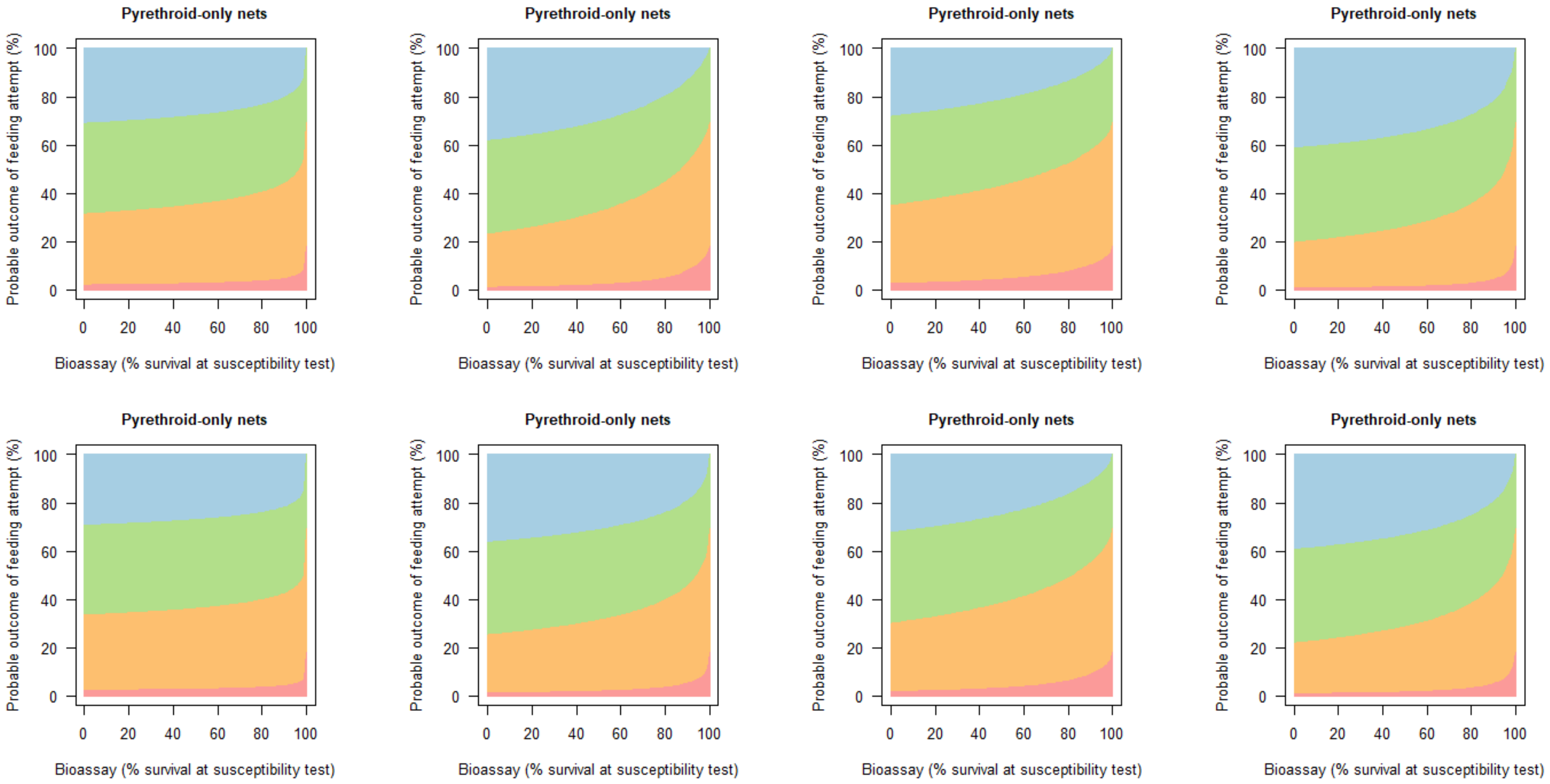

Figure S5. Uncertainty estimates for the probability plots for the outcome of a mosquito feeding attempt in the presence of pyrethroid-only ITNs. Each iteration of the figure is constructed from the posterior-distributions of the fitting process and illustrates the uncertainty in ITN parameters. This uncertainty is used within the epidemiological predictions shown in Figure 2 of the main text.

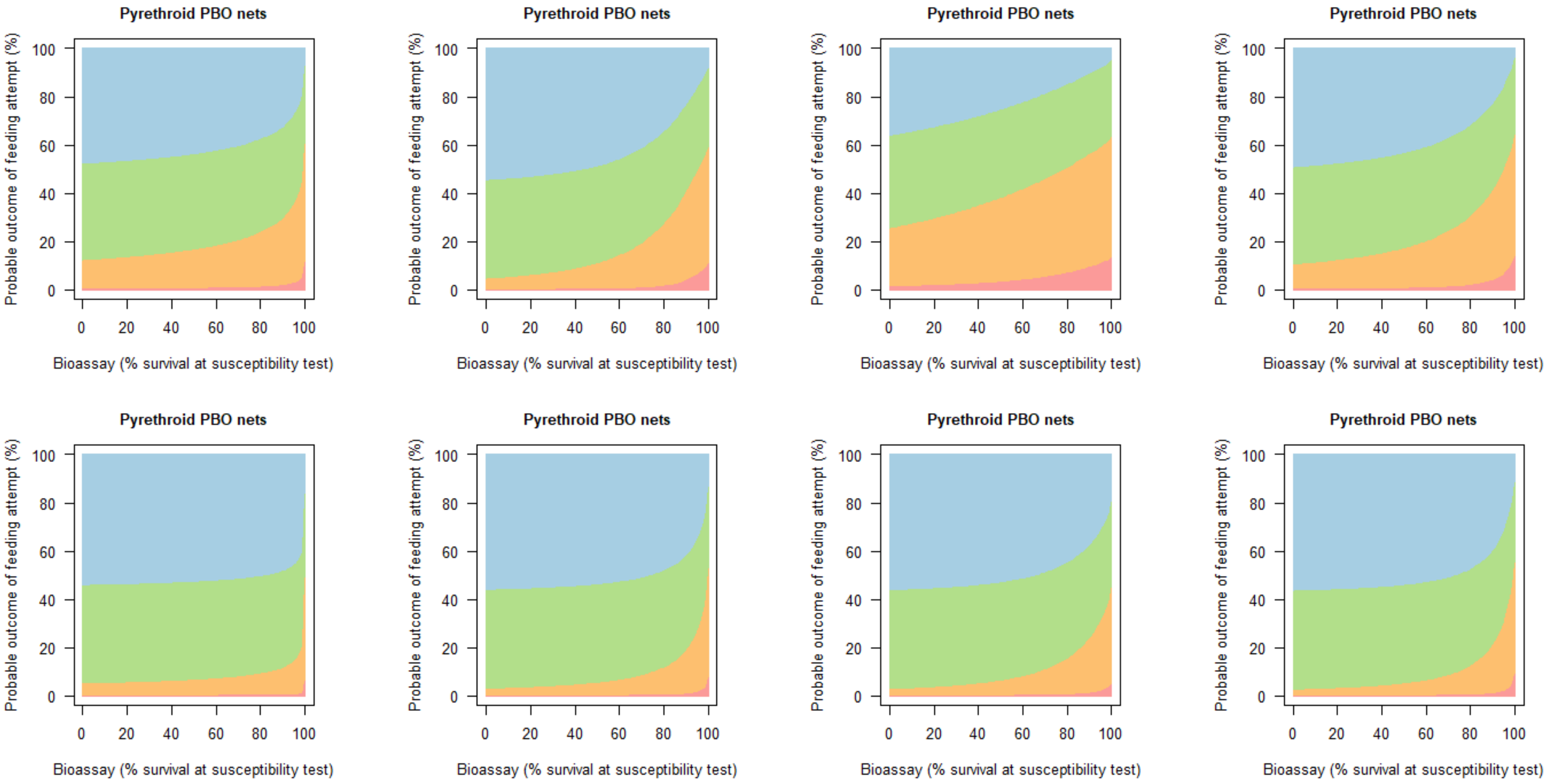

Figure S6. Uncertainty estimates for the probability plots for the outcome of a mosquito feeding attempt in the presence of pyrethroid-PBO ITNs. Each iteration of the figure is constructed from the posterior-distributions of the fitting process and illustrates the uncertainty in ITN parameters. This uncertainty is used within the epidemiological predictions shown in Figure 2 of the main text.

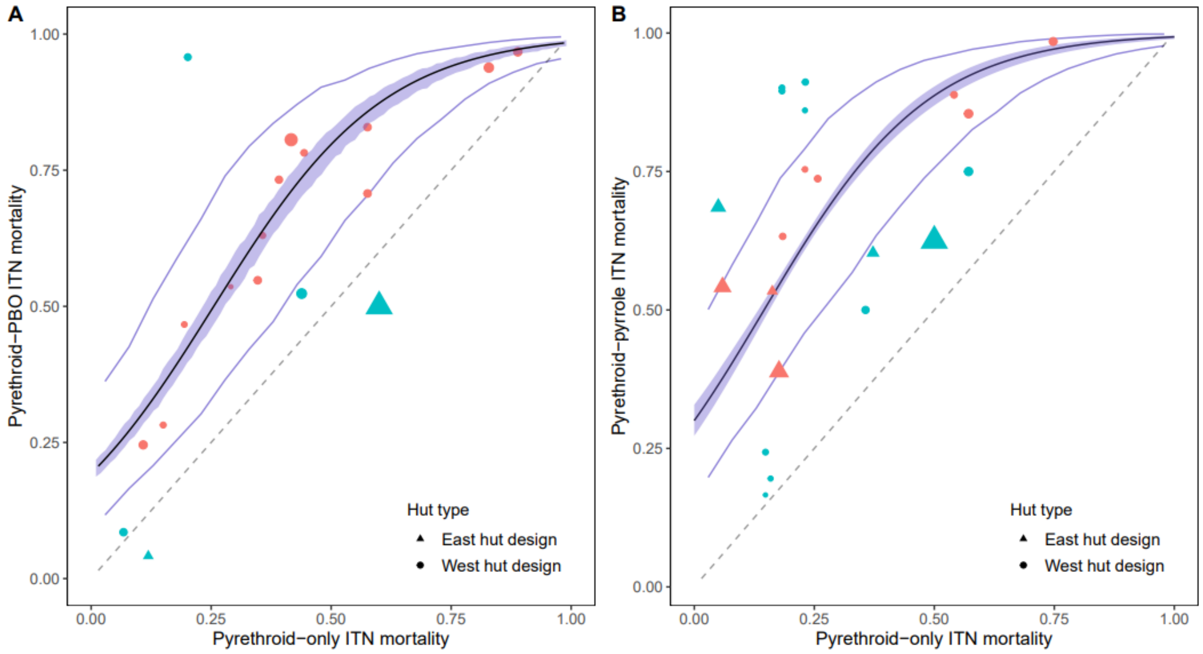

Figure S7. The association between mortality induced by different types of ITNs and whether each individual comparison is an outlier. Experimental hut trials are used to assess the increased mosquito mortality caused by (A) pyrethroid-PBO compared to pyrethroid-only ITNs measured over 24 hours, or (B) pyrethroid-pyrrole compared to pyrethroid-only ITNs measured over 72 hours. Points show results of a systematic review of trials comparing two nets, with the point shape indicating the design of experimental hut trial used and point size indicating number of mosquitoes caught. Solid black blue line indicates the best fit model whilst the blue shaded area indicates uncertainty (95% credible intervals) around this best fit line. The experimental hut trial has relatively high measurement error. Methods for estimating the uncertainty generated by this measurement error are outlined by Challenger *et al.*[36]. Wider uncertainty is expected if the mean number of mosquitoes collected per hut per night is low, or the between-observation variability is high. Here we use conservative estimates of these metrics, assuming a mean of 5 mosquitoes per hut per night and a between-observation variance of 1.5 (expressed on the log-odds scale). Results, generated by simulating 60000 trials, are shown by the two blue lines which indicate the 95% range for studies with the above characteristics. Points which fall outside this region are defined as outliers (and are shown in turquoise), whilst studies inside these lines (coloured red) would be classified as within the expected uncertainty of the assay.

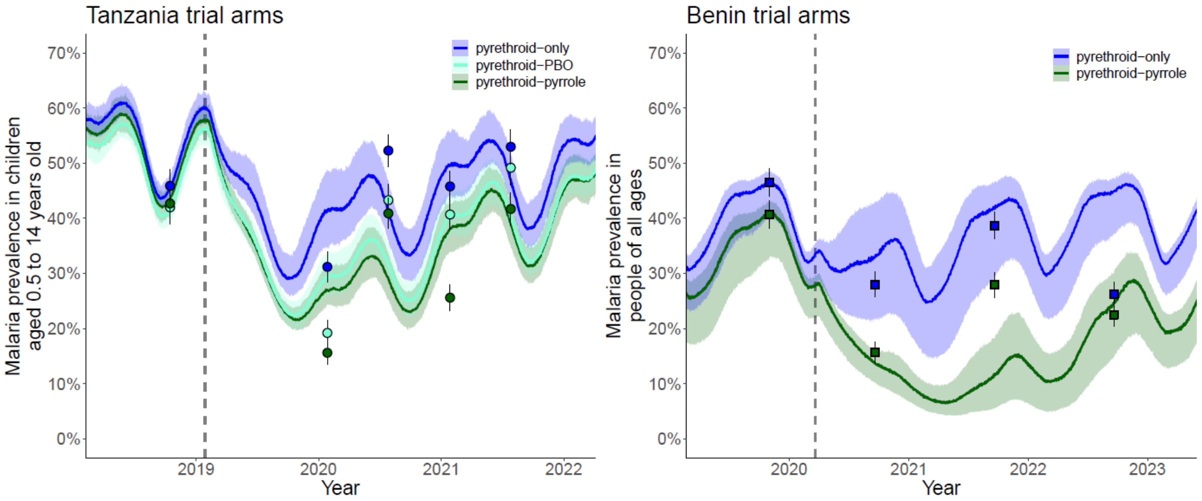

Figure S8. The ability of the model to predict epidemiological impact of novel insecticide treated nets in the two cluster randomised trials with lower levels of uncertainty in entomological efficacy. (A-B) Changes in malaria prevalence following ITN distribution in the Tanzania and Benin cluster randomised control trials[14–17]. Points indicate observed disease prevalence with 95% confidence intervals whilst solid line showing model projections (different age groups in each study), with dark blue, turquoise, and dark green denoting pyrethroid-only, pyrethroid-PBO and pyrethroid-pyrrole ITNs respectively. In all plots, shaded area around lines indicate uncertainty in projections caused by uncertainty in the best fit ITN efficacy parameters using the model with a binomial likelihood (the shaded area of Figure 1A) and should be compared to projections in Figure 2A-B which show the full uncertainty in the added benefit of pyrethroid-pyrrole ITNs (expressed as the dotted lines in Figure 1A).

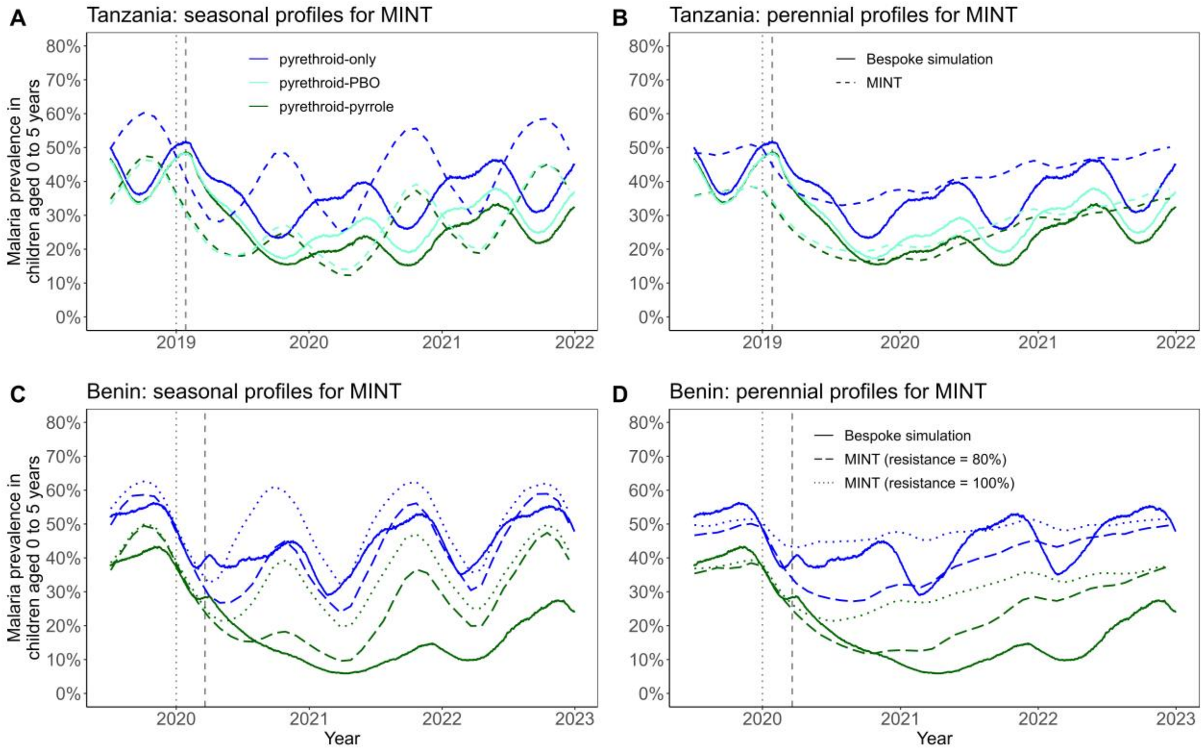

Figure S9. Comparison of MINT outputs and bespoke simulations. Bespoke simulation results of two trials in Tanzania (**A** & **B**) and Benin (**C** & **D**) are compared to the closest equivalent simulation outputs from MINT using the parameters in Table S5. Both seasonal (**A** & **C**) and perennial (**B** & **D**) MINT options are displayed for comparison, and for the Benin trial, two levels of resistance are displayed as the trial value lies between two MINT options (Table S5). Vertical dotted grey lines at January 1st show when the MINT simulations assumed insecticide treated nets were distributed while dashed grey lines show when the trial distribution was assumed to occur in bespoke simulations. Time series lines show modelled prevalence in children aged 0 to 5 years, coloured by net type and with line types distinguishing between bespoke simulations and MINT outputs. For these MINT outputs, the relative reduction in mean annual clinical cases of malaria in children aged 0 to 5 years across three years post-distribution was calculated for pyrethroid-PBO and pyrethroid-pyrrole ITN simulations in comparison to pyrethroid-only simulations. For Tanzania, relative reduction in cases was 38.2% and 47.3% for pyrethroid-PBO and pyrethroid-pyrrole ITNs, respectively assuming seasonal transmission and 40.8% and 45.7% respectively assuming perennial transmission. This is slightly lower than the bespoke model which predicted a 42% and a 56% reduction, though this is expected as ITN use remained high (Table 1 of main manuscript). For Benin assuming 80% pyrethroid resistance, relative reduction in cases was 43.7% for pyrethroid-pyrrole ITNs assuming seasonal transmission and 46.1% assuming perennial transmission. For Benin assuming 100% pyrethroid resistance, relative reduction in cases was 38.7% and 38.8% for pyrethroid-pyrrole ITNs assuming seasonal and perennial transmission, respectively. These estimates are lower than the 74% efficacy predicted by the bespoke model, again because ITN use remained higher than MINT assumptions.

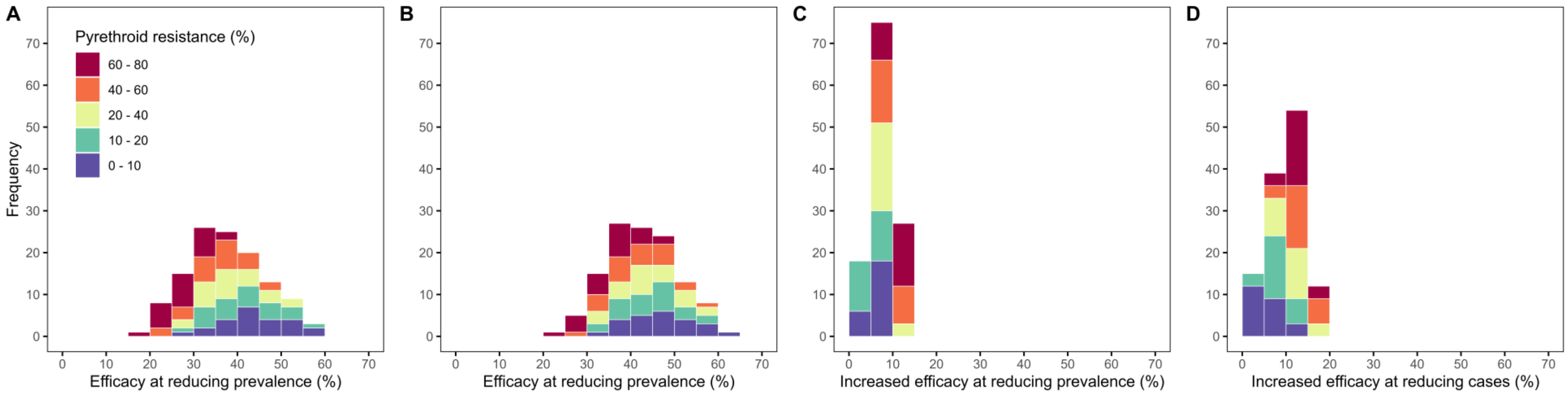

Figure S10. Projected epidemiological benefit of pyrethroid-pyrrole over pyrethroid-PBO ITNs in different settings in Africa. Model estimates of the efficacy of pyrethroid-PBO (A) and pyrethroid-pyrrole (B) ITNs to reduce mean malaria prevalence over three years following a mass distribution campaign (by microscopy, all ages). Chart shows the frequency distribution of efficacy estimates from 240 model simulations in a site with a 40% malaria prevalence, 40% historical pyrethroid-only ITN use, a mosquito population with <90% resistance, and no history of IRS. Efficacy is calculated as the reduction in malaria prevalence resulting from a new ITN campaign which initially achieves 80% usage compared to a scenario when no campaign takes place. Bars are coloured according to the level of pyrethroid resistance in the local mosquito population. The projected percentage relative increase in efficacy from switching from pyrethroid-only to pyrethroid-pyrrole ITNs on mean prevalence (C) and clinical cases averted (D).

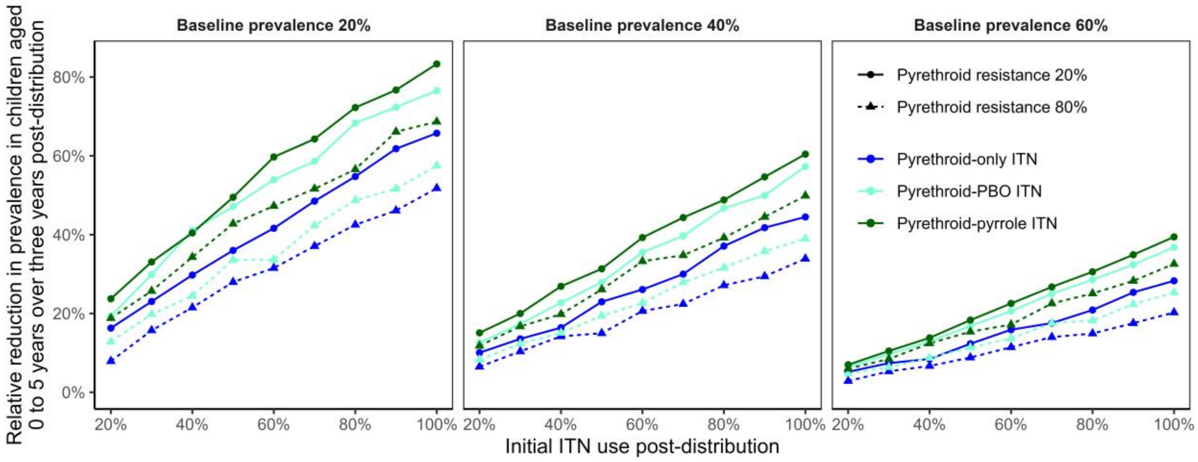

Figure S11. Sensitivity analysis of the impact of percentage use of insecticide treated nets on reduction in prevalence in MINT simulations. The impact of increasing initial use of insecticide treated nets on the relative reduction in prevalence in children aged 0 to 5 years compared to a scenario with no intervention, is shown for three net types, three baseline prevalence levels and two levels of pyrethroid resistance. Each point represents a single simulation and a total of 162 MINT outputs are analysed, with varying baseline prevalence levels (left panel = 20%, middle panel = 40% and right panel = 60%), net types (pyrethroid-only, pyrethroid-PBO and pyrethroid-pyrrole with differently coloured lines and points) and pyrethroid resistance levels (solid lines with circle points = 20%, dashed lines with triangle points = 80%). Reduction in prevalence is calculated as the relative percentage reduction in prevalence over three years post-distribution compared to a simulation with the same baseline parameters but no distribution event. For all simulations, it is assumed that transmission occurs seasonally, mosquitoes have a human blood index of 87%, 97% of mosquitoes feed when people are indoors, historic insecticide treated net use is 40% and no indoor residual spraying is used either historically or during the intervention years.

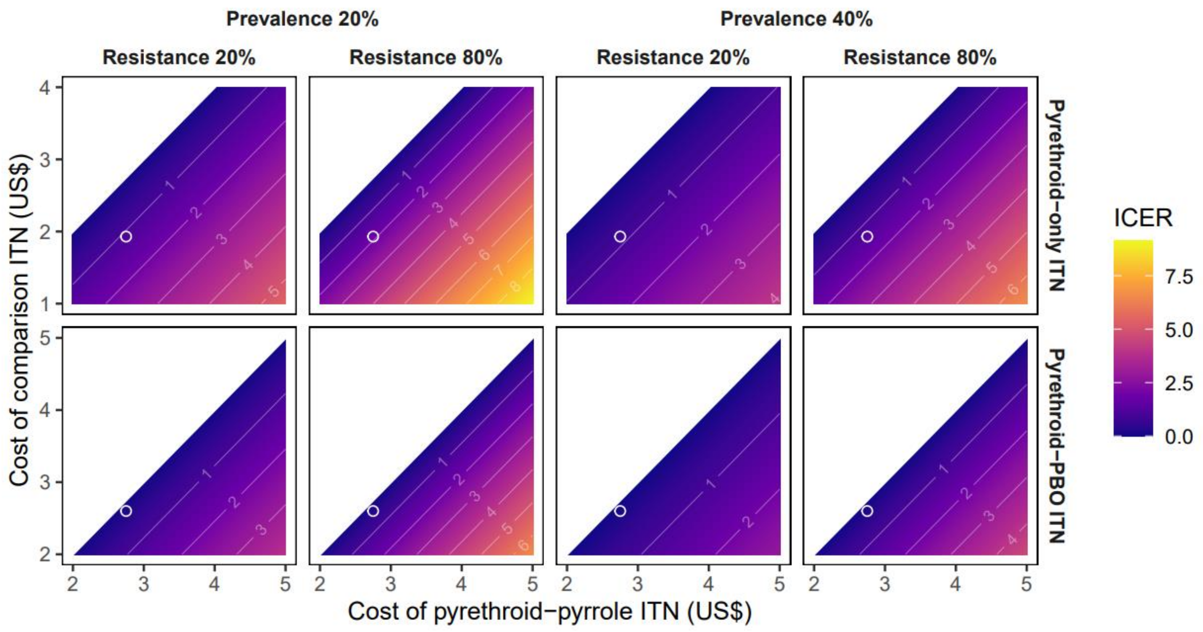

Figure S12. Incremental cost-effectiveness ratios for comparisons between different ITNs. Pyrethroid-pyrrole ITNs are compared with pyrethroid-only (top row) and pyrethroid-PBO (bottom row) ITNs using the incremental cost per additional clinical malaria case averted, for a range of product prices. Decision-making plots are shown for different levels of baseline prevalence (left two columns 20% and right two columns 40%) and pyrethroid resistance (first and third columns 20% and second and fourth columns 80%). Coloured shading shows incremental cost-effectiveness ratios for comparisons between ITNs at each cost combination. ICER values should be interpreted as the additional cost, in US$, required to avert an additional clinical case of malaria per year over the 3 years post ITN distribution using pyrethroid-pyrrole ITNs, compared to either pyrethroid-only or pyrethroid-PBO ITNs. Results show the ICER varies according to the areas malaria prevalence and the level of resistance in the local mosquito population, though estimates are generally very low indicating it is highly cost-effective to switch ITNs. Values where pyrethroid-pyrrole ITNs are cheaper than the comparison net are excluded (negative ICER's parameter space is white) as this is a very unlikely scenario at present. Contour lines and ICER values at regular intervals have been added to plots to ease interpretation. The range of costs presented are illustrative and do not necessarily represent current market price ranges. However, open white circles locate positions that correspond to a potential set of realistic current product prices ($1.93 for pyrethroid-only, $2.60 for pyrethroid-PBO and $2.75 for pyrethroid-pyrrole ITNs, see Global Fund 2023 pooled procurement price https://www.theglobalfund.org/en/sourcing-management/health-products/long-lasting-insecticidal-nets/). Note that the costs explored for pyrethroid-only ITNs are slightly lower than those of the other two ITN types, in line with current pricing. Simulations assume mosquitoes have a human blood index of 87%, 97% of mosquitoes feed when people are indoors, malaria transmission is seasonal, with a historical ITN usage of 40%, no indoor residual spraying and an expected ITN coverage of 80%.

3 Supplementary Tables

Table S6. Estimates of different levels of uncertainty in model parasite prevalence estimates. Table shows parasite prevalence estimates at different time points measured in the two trials, with 95% credible intervals in brackets. Uncertainty in entomological efficacy of the ITN is assessed using either the binomial distribution (showing uncertainty around the best-fit line, shaded area in Figure A1) or the beta-binomial (showing total uncertainty in hut trial outcomes, dashed lines in Figure A1).

Country	Net used in study arm	Time (month)	Model	Model derived – trial mixture of nets		
				Predicted prevalence (uncertainty)	Predicted efficacy (uncertainty)	Overall Efficacy (uncertainty)
Tanzania	Pyrethroid-pyrrole	12	beta-binomial	0.25 (0.2-0.36)	39% (11%-56%)	57% (38%-66%)
Tanzania	Pyrethroid-pyrrole	12	binomial	0.27 (0.24-0.31)	34% (28%-40%)	55% (49%-60%)
Tanzania	Pyrethroid-pyrrole	18	beta-binomial	0.27 (0.2-0.37)	36% (9%-53%)	50% (32%-62%)
Tanzania	Pyrethroid-pyrrole	18	binomial	0.29 (0.25-0.34)	29% (24%-34%)	46% (40%-52%)
Tanzania	Pyrethroid-pyrrole	24	beta-binomial	0.35 (0.28-0.46)	29% (7%-46%)	42% (25%-55%)
Tanzania	Pyrethroid-pyrrole	24	binomial	0.38 (0.34-0.42)	23% (18%-28%)	38% (32%-44%)
Tanzania	Pyrethroid-pyrrole	30	beta-binomial	0.37 (0.31-0.44)	22% (7%-36%)	33% (22%-43%)
Tanzania	Pyrethroid-pyrrole	30	binomial	0.39 (0.35-0.43)	16% (12%-21%)	29% (24%-37%)
Tanzania	Pyrethroid only	12	beta-binomial	0.42 (0.36-0.5)		32% (21%-41%)
Tanzania	Pyrethroid only	12	binomial	0.41 (0.35-0.49)		33% (23%-43%)
Tanzania	Pyrethroid only	18	beta-binomial	0.43 (0.37-0.48)		25% (16%-33%)
Tanzania	Pyrethroid only	18	binomial	0.41 (0.36-0.47)		27% (17%-35%)
Tanzania	Pyrethroid only	24	beta-binomial	0.51 (0.45-0.55)		20% (14%-27%)
Tanzania	Pyrethroid only	24	binomial	0.5 (0.44-0.56)		21% (12%-30%)
Tanzania	Pyrethroid only	30	beta-binomial	0.48 (0.43-0.51)		17% (11%-24%)
Tanzania	Pyrethroid only	30	binomial	0.47 (0.43-0.52)		18% (11%-25%)
Tanzania	Pyrethroid-PBO	12	beta-binomial	0.3 (0.25-0.4)	27% (5%-43%)	49% (33%-59%)
Tanzania	Pyrethroid-PBO	12	binomial	0.29 (0.26-0.34)	30% (16%-39%)	50% (42%-56%)
Tanzania	Pyrethroid-PBO	18	beta-binomial	0.33 (0.26-0.4)	23% (6%-35%)	40% (27%-51%)
Tanzania	Pyrethroid-PBO	18	binomial	0.31 (0.28-0.35)	25% (14%-32%)	42% (35%-47%)
Tanzania	Pyrethroid-PBO	24	beta-binomial	0.41 (0.35-0.47)	18% (5%-30%)	33% (21%-43%)
Tanzania	Pyrethroid-PBO	24	binomial	0.4 (0.37-0.44)	20% (12%-25%)	34% (27%-38%)
Tanzania	Pyrethroid-PBO	30	beta-binomial	0.41 (0.37-0.45)	13% (2%-22%)	26% (17%-34%)
Tanzania	Pyrethroid-PBO	30	binomial	0.4 (0.37-0.43)	14% (8%-20%)	27% (21%-32%)
Benin	Pyrethroid-pyrrole	6	beta-binomial	0.14 (0.1-0.27)	60% (27%-71%)	66% (32%-71%)
Benin	Pyrethroid-pyrrole	6	binomial	0.14 (0.1-0.16)	59% (52%-63%)	67% (61%-72%)
Benin	Pyrethroid-pyrrole	18	beta-binomial	0.12 (0.04-0.35)	72% (20%-91%)	69% (13%-90%)
Benin	Pyrethroid-pyrrole	18	binomial	0.12 (0.05-0.18)	72% (61%-83%)	72% (57%-86%)
Benin	Pyrethroid-pyrrole	30	beta-binomial	0.24 (0.09-0.38)	44% (16%-80%)	39% (7%-78%)
Benin	Pyrethroid-pyrrole	30	binomial	0.25 (0.15-0.3)	45% (35%-61%)	41% (28%-62%)
Benin	Pyrethroid only	6	beta-binomial	0.36 (0.21-0.42)		24% (11%-50%)
Benin	Pyrethroid only	6	binomial	0.33 (0.22-0.43)		28% (9%-49%)
Benin	Pyrethroid only	18	beta-binomial	0.43 (0.3-0.46)		8% (3%-34%)
Benin	Pyrethroid only	18	binomial	0.42 (0.3-0.47)		11% (1%-34%)
Benin	Pyrethroid only	30	beta-binomial	0.45 (0.38-0.47)		4% (0%-18%)
Benin	Pyrethroid only	30	binomial	0.45 (0.37-0.47)		5% (1%-19%)

4 List of Appendices

Appendix 2. Comparison of experimental hut trial mortality of pyrethroid-only, pyrethroid-PBO and pyrethroid-pyrrole ITNs. Unpublished studies have been excluded from the systematic review.

Appendix 3. Parameter estimates for pyrethroid-only ITNs for varying levels of pyrethroid resistance. This file provides updated estimates of d_{N0}, r_{N0} and γ and how this changes according to the level of pyrethroid resistance measured in the local mosquito population using a discriminatory dose bioassay.

Appendix 4. Parameter estimates for pyrethroid-PBO ITNs for varying levels of pyrethroid resistance. This file provides updated estimates of d_{N0}, r_{N0} and γ and how this changes according to the level of pyrethroid resistance measured in the local mosquito population using a discriminatory dose bioassay.

Appendix 5. Parameter estimates for pyrethroid-pyrrole ITNs for varying levels of pyrethroid resistance. This file provides estimates of d_{N0}, r_{N0} and γ and how this changes according to the level of pyrethroid resistance measured in the local mosquito population using a discriminatory dose bioassay.

Appendix 6. MINT Version 2 user guide. A description of how to use the MINT tool including methods for identifying the optimum allocation of resources across regions within a defined budget.

5 Full references (main paper and supplements)

1 Nash RK, Lambert B, N'Guessan R, *et al.* Systematic review of the entomological impact of insecticide-treated nets evaluated using experimental hut trials in Africa. *Current Research in Parasitology & Vector-Borne Diseases* 2021; **1**: 100047.

2 Okumu FO, Finda MF. 3. Creating long-term resilience against malaria vectors while addressing the immediate need to suppress pathogen transmission. In: Innovative strategies for Vector Control. 2021: 33–57.

3 Stan Development Team. Rstan: The R interface to stan. 2023. https://mc-stan.org/.

4 Stan Development Team. shinystan: Interactive visual and numerical diagnostics and posterior analysis for Bayesian models. 2017 http://mc-stan.org/.

5 Churcher TS, Lissenden N, Griffin JT, Worrall E, Ranson H. The impact of pyrethroid resistance on the efficacy and effectiveness of bednets for malaria control in Africa. *Elife* 2016; **5**. DOI:10.7554/eLife.16090.

6 World Health Organization. The evaluation process for vector control products. 2017.

7 Massue DJ, Kisinza WN, Malongo BB, *et al.* Comparative performance of three experimental hut designs for measuring malaria vector responses to insecticides in Tanzania. *Malar J* 2016; **15**: 165.

8 Griffin JT, Hollingsworth TD, Okell LC, *et al.* Reducing Plasmodium falciparum malaria transmission in Africa: a model-based evaluation of intervention strategies. *PLoS Med* 2010; **7**: e1000324.

9 Curtis CF, Myamba J, Wilkes TJ. Comparison of different insecticides and fabrics for anti-mosquito bednets and curtains. *Med Vet Entomol* 1996; **10**: 1–11.

10 Mathenge EM, Gimnig JE, Kolczak M, Ombok M, Irungu LW, Hawley WA. Effect of Permethrin-Impregnated Nets on Exiting Behavior, Blood Feeding Success, and Time of Feeding of Malaria Mosquitoes (Diptera: Culicidae) in Western Kenya. *J Med Entomol* 2001; **38**: 531–6.

11 Lines JD, Myamba J, Curtis CF. Experimental hut trials of permethrin-impregnated mosquito nets and eave curtains against malaria vectors in Tanzania. *Med Vet Entomol* 1987; **1**: 37–51.

12 Charles G, Winskill P, Topazian H, *et al.* malariasimulation: An individual based model for malaria. R package version 1.6.0. 2023.

13 Sherrard-Smith E, Winskill P, Hamlet A, *et al.* Optimising the deployment of vector control tools against malaria: a data-informed modelling study. *Lancet Planet Health* 2022; published online Jan 20. DOI:10.1016/S2542-5196(21)00296-5.

14 Accrombessi M, Cook J, Dangbenon E, *et al.* Effectiveness of pyriproxyfen-pyrethroid and chlorfenapyr-pyrethroid long-lasting insecticidal nets (LLINs) compared with pyrethroid-only LLINs for malaria control in the third year post-distribution: a secondary analysis of a cluster-randomised controlle. *Lancet Infect Dis* 2024; **3099**: 2–4.

15 Mosha JF, Matowo NS, Kulkarni MA, *et al.* Effectiveness of long-lasting insecticidal nets with pyriproxyfen–pyrethroid, chlorfenapyr–pyrethroid, or piperonyl butoxide–

pyrethroid versus pyrethroid only against malaria in Tanzania: final-year results of a four-arm, single-blind, cluster-randomised . *Lancet Infect Dis* 2024; **24**: 87–97.

16 Accrombessi M, Cook J, Dangbenon E, *et al.* Efficacy of pyriproxyfen-pyrethroid long-lasting insecticidal nets (LLINs) and chlorfenapyr-pyrethroid LLINs compared with pyrethroid-only LLINs for malaria control in Benin: a cluster-randomised, superiority trial. *The Lancet* 2023; **401**: 435–46.

17 Mosha JF, Kulkarni MA, Lukole E, *et al.* Effectiveness and cost-effectiveness against malaria of three types of dual-active-ingredient long-lasting insecticidal nets (LLINs) compared with pyrethroid-only LLINs in Tanzania: a four-arm, cluster-randomised trial. *The Lancet* 2022; **399**: 1227–41.

18 Atieli HE, Zhou G, Afrane Y, *et al.* Insecticide-treated net (ITN) ownership, usage, and malaria transmission in the highlands of western Kenya. *Parasit Vectors* 2011; **4**: 1–10.

19 Koenker H, Taylor C, Burgert-Brucker CR, Thwing J, Fish T, Kilian A. Quantifying seasonal variation in insecticide-treated net use among those with access. *American Journal of Tropical Medicine and Hygiene* 2019; **101**: 371–82.

20 Koenker H, Kilian A. Recalculating the Net Use Gap: A Multi-Country Comparison of ITN Use versus ITN Access. *PLoS One* 2014; **9**: e97496.

21 Carpenter B, Gelman A, Hoffman MD, *et al.* Stan: A probabilistic programming language. *J Stat Softw* 2017; **76**.

22 Bertozzi-Villa A, Bever CA, Koenker H, *et al.* Maps and metrics of insecticide-treated net access, use, and nets-per-capita in Africa from 2000-2020. *Nat Commun* 2021; **12**: 3589.

23 Charles G, Winskill P, Topazian H, *et al.* malariasimulation: An individual based model for malaria. 2022.

24 Killeen GF, Kiware SS, Okumu FO, *et al.* Going beyond personal protection against mosquito bites to eliminate malaria transmission: population suppression of malaria vectors that exploit both human and animal blood. *BMJ Glob Health* 2017; **2**: e000198.

25 Sherrard-Smith E, Skarp JE, Beale AD, *et al.* Mosquito feeding behavior and how it influences residual malaria transmission across Africa. *Proc Natl Acad Sci U S A* 2019; **116**: 15086–96.

26 Massey NC, Garrod G, Wiebe A, *et al.* A global bionomic database for the dominant vectors of human malaria. *Sci Data* 2016; **3**: 160014.

27 Sherrard-Smith E, Ngufor C, Sanou A, *et al.* Inferring the epidemiological benefit of indoor vector control interventions against malaria from mosquito data. *Nat Commun* 2022; **13**: 3862.

28 White MT, Griffin JT, Churcher TS, Ferguson NM, Basáñez M-G, Ghani AC. Modelling the impact of vector control interventions on Anopheles gambiae population dynamics. *Parasit Vectors* 2011; **4**: 153.

29 National Weather Service. Climate Prediction Center. .

30 Garske T, Ferguson NM, Ghani AC. Estimating Air Temperature and Its Influence on Malaria Transmission across Africa. *PLoS One* 2013; **8**: e56487.

31 Winskill P. _cali: Good vibes and model calibration_. 2023.

32 Sherrard-Smith E, Griffin JT, Winskill P, *et al.* Systematic review of indoor residual spray efficacy and effectiveness against Plasmodium falciparum in Africa. *Nat Commun* 2018; **9**: 4982.

33 Owusu HF, Müller P. How important is the angle of tilt in the WHO cone bioassay? *Malar J* 2016; **15**: 243.

34 Owusu HF, Jančáryová D, Malone D, Müller P. Comparability between insecticide resistance bioassays for mosquito vectors: time to review current methodology? *Parasit Vectors* 2015; **8**: 357.

35 Hughes A, Matope A, Emery M, *et al.* A closer look at the WHO cone bioassay: video analysis of the hidden effects of a human host on mosquito behaviour and insecticide contact. *Malar J* 2022; **21**: 1–11.

36 Challenger JD, Nash RK, Ngufor C, *et al.* Assessing the variability in experimental hut trials evaluating insecticide-treated nets against malaria vectors. *Current Research in Parasitology and Vector-Borne Diseases* 2023; **3**. DOI:10.1016/j.crpvbd.2023.100115.

37 Kweyamba PA, Hofer LM, Kibondo UA, *et al.* Sub-lethal exposure to chlorfenapyr reduces the probability of developing Plasmodium falciparum parasites in surviving Anopheles mosquitoes. *Parasit Vectors* 2023; **16**: 1–9.

38 Koenker H, Kilian A. Recalculating the Net Use Gap: A Multi-Country Comparison of ITN Use versus ITN Access. *PLoS One* 2014; **9**: e97496.

39 Kilian A, Boulay M, Koenker H, Lynch M. How many mosquito nets are needed to achieve universal coverage? Recommendations for the quantification and allocation of long-lasting insecticidal nets for mass campaigns. *Malar J* 2010; **9**: 1–9.

40 Koenker HM, Yukich JO, Mkindi A, *et al.* Analysing and recommending options for maintaining universal coverage with long-lasting insecticidal nets: the case of Tanzania in 2011. *Malar J* 2013; **12**: 150.

41 Bertozzi-Villa A, Bever CA, Koenker H, *et al.* Maps and metrics of insecticide-treated net access, use, and nets-per-capita in Africa from 2000-2020. *Nat Commun* 2021; **12**: 3589.
