# Supplementary material for: The epidemiological benefit of pyrethroid–pyrrole insecticide treated nets against malaria: an individual-based malaria transmission dynamics modelling study
Source: Lancet Glob Health. 2024 Nov 20;12(12):e1973–83. doi: 10.1016/S2214-109X(24)00329-2 (PMC11584316; doi:10.1016/S2214-109X(24)00329-2)
Supplement: Supplementary appendix 6 [file mmc6.pdf]

THE LANCET
Global Health

Supplementary appendix 6

This appendix formed part of the original submission and has been peer reviewed. We post it as supplied by the authors.

Supplement to: Churcher TS, Stopard IJ, Hamlet A, et al. The epidemiological benefit of pyrethroid–pyrrole insecticide treated nets against malaria: an individual-based malaria transmission dynamics modelling study. *Lancet Glob Health* 2024; **12:** e1973–83.

MINT – Malaria INtervention Tool

An online tool to support malaria control decision-making.

User Guide

Version 2.0

Contents

1 BACKGROUND 3
1.1 What is the problem? 3
1.2 What are the benefits of MINT? 4
1.3 What can the tool do? 4
1.4 What the model cannot do 4
1.5 Limitations and critical modelling assumptions 5
1.5.1 Major model assumptions 5
1.5.2 Intervention assumptions 6
1.5.3 Cost assumptions 7
1.6 Validating MINT: How well can the transmission model simulate interventions? 8
1.7 What size area should be considered? 8
1.8 Where can it be found? 8
2 STEP-BY-STEP USER GUIDE 9
2.1 Setup baseline 10
2.1.1 Size of population at risk 12
2.1.2 Seasonality of transmission 12
2.1.3 Current malaria prevalence 12
2.1.4 Preference for biting indoors 13
2.1.5 Preference for biting people 13
2.1.6 Level of pyrethroid resistance 13
2.1.7 ITN population usage in last survey (%) 14
2.1.8 IRS population coverage in last survey (%) 14
2.2 Future intervention 14
2.2.1 Expected ITN population use 14
2.2.2 Expected IRS coverage 15
2.3 Procurement and distribution 15
2.3.1 When planning procurement, what number of people per net is used? 15
2.3.2 What percentage is your procurement buffer, if used? (%) 15
2.4 Price of interventions 15
2.4.1 Price of pyrethroid-only ITN ($USD) 16
2.4.2 Price of pyrethroid-PBO ITN ($USD) 16
2.4.3 Price of pyrethroid-pyrrole ITN ($USD) 16
2.4.4 ITN mass distribution campaign delivery cost per person ($USD) 16
2.4.5 Annual cost of IRS per person ($USD) 16
3 SINGLE REGION INTERPRETATION 17
3.1 Impact graphs 17
3.2 Impact table 18
3.3 Cost effectiveness graphs 19
3.4 Cost effectiveness table 20
4 MULTIPLE REGION ANALYSIS 21
4.1 Strategy interpretation 22
4.2 Strategize charts 23
4.3 Strategize table 24
MINT methodology 26
References 28

1 BACKGROUND

Key definitions

cRCT – Cluster randomised control trial with epidemiological endpoints.

ITN – Insecticide treated mosquito net, also known as a long-lasting insecticidal net (LLIN).

IRS – Indoor residual spraying of insecticide. A typical long-lasting IRS product is assumed (for example Actellic 300CS or Sumishield, using parameters from (1)) and the insecticide used should be rotated as part of insecticide resistance management plan.

Pyrethroid-only ITN – An ITN containing pyrethroid insecticide only. Widely used since 2000, this intervention is now showing signs of diminished efficacy in areas with highly pyrethroid-resistant mosquitoes.

Pyrethroid-PBO ITN – An ITN which contains pyrethroid insecticide in addition to the synergist piperonyl butoxide (PBO). This newer intervention class kills insecticide-resistant mosquitoes by neutralising the enzymes responsible for pyrethroid resistance. The epidemiological benefit of these nets has been demonstrated compared to pyrethroid-only ITNs in cRCTs (2,3), though their impact on highly pyrethroid-resistant mosquitoes and long-term durability is presently unclear.

Pyrethroid-pyrrole ITN – A mosquito net containing two active ingredients, pyrethroid and an insecticide of the pyrrole class, such as chlorfenapyr. This newest type of ITN has shown epidemiological benefit over pyrethroid-only ITNs in two recent cRCTs (4,5) and is now included in the WHO Guidelines for malaria.

1.1 What is the problem?

Insecticide treated mosquito nets (ITNs, also called long-lasting insecticidal nets or LLINs) and indoor residual spraying of insecticides (IRS) have been the key vector control interventions for malaria in the past 20 years. However, mosquitoes that transmit malaria are becoming increasingly resistant to pyrethroid insecticides, the active ingredient of traditional ITNs distributed at scale across malaria-endemic countries (6). Resistance to other insecticides is also increasing (7,8). In response, the global community has been developing new vector control interventions to mitigate for the diminishing protection provided by pyrethroid-only ITNs.

These interventions have different prices, and different durations of activity at killing, deterring and inhibiting blood-feeding of local mosquitoes. Depending on patterns of use and local ecology, the impact of these interventions is spatially and temporally variable. Additionally, there are limits on national malaria control budgets, and products with better public health benefit tend to cost more, so recommending a new product, with slightly better potential, may reduce the quantity purchased and perversely increase disease. There is considerable challenge in determining which interventions to use in regions with different population sizes, historic use of interventions, mosquito ecology and local customs, jobs or habits that may lead to varied exposure to infectious mosquito bites for residents. How to strategize combinations of interventions with a limited budget is an important public health problem.

1.2 What are the benefits of MINT?

Interventions will have differing epidemiological impact given the underlying characteristics of a region. The user-friendly interface allows access to results from a malaria transmission model that explores the potential epidemiological impact of deploying multiple vector control tools. The tool incorporates a flexible cost-effectiveness analysis to enable the allocation across different regions to be explored.

1.3 What can the tool do?

The vector control decisions tool is designed to help National Malaria Control Programs (NMCP) explore the most cost-effective current World Health Organization (WHO) recommended ITN and/or IRS products for *Plasmodium falciparum* malaria control. The current tool allows inputs of mosquito bionomics that are typical of the dominant vector species within Africa, and care should be taken extrapolating results elsewhere where vector species might be more diverse. Local human, mosquito and cost data are used to characterise the setting of interest (referred to as a 'region' in the tool). Version 2.0 of the tool has all the functionality of version 1.0 but with the addition of new ITNs recently recommended by the WHO.

Version 2.0 also allows the impact of different vector control interventions to be assessed across up to 15 regions to explore the potential cost-effectiveness of each intervention package. A maximum budget can be set to help determine the most appropriate and affordable combination of interventions to deploy across regions to avert the most cases over three years.

1.4 What the model cannot do

The results of mathematical models are no substitute for high-quality local surveillance data and the understanding from local researchers. The purpose of MINT is to provide additional information to support local decision-making, and MINT recommendations should be considered alongside other local information. MINT projections are made using average entomological and epidemiological data from systematic reviews that gather data from across Africa and so may not be representative of specific settings. For example, IRS efficacy is thought to vary according to the type of household wall material, which varies between sites (9,10) and any post-spray modifications could impact the overall performance of a product (11). These differences and uncertainties should be considered in any decision-making process. Similarly, projections from the model are only as good as data used to parameterise it, so the simulations may be more useful where local data are well understood. Simplifications have been made (for example, in the range of endemicity settings that are explored) so individual estimates of impact and cost-effectiveness will be slightly different given these simplifications. When input parameters are uncertain or fall between the range of values available in MINT then simulations should be repeated, varying the input parameter of interest, to determine whether this changes the optimal policy decision. Though the model derived cost-effectiveness estimate may vary, the relative difference between intervention options is likely to be more consistent.

This tool does not project deaths averted due to the use of any vector control intervention. In the model simulations, treatment of clinical cases is assumed to be consistent at a level broadly consistent with Africa. Deaths due to malaria depends on the ability of the local health system to promptly treat clinical cases and so the relationship between cases and deaths might vary

substantially within and between regions. All cost-effectiveness analyses are based on cases averted, and results may vary for deaths if health systems and the availability of treatment vary.

The projections of impact have been shown to adequately reflect changes in malaria prevalence observed in the cRCTs (Sherrard-Smith et al. 2022) affording some degree of confidence in the epidemiological outputs of MINT.

1.5 Limitations and critical modelling assumptions

As with any model, there are limitations and assumptions that need to be communicated to help with the interpretation of results. MINT version 2.0 uses a smaller population size in epidemiological projections to expedite the update process. This has increased the amount of random variability present and means that results from MINT version 2.0 should be treated with caution when there are small differences between interventions. This will be corrected in future versions of the tool by running the simulations for large populations which generally smooths projected outcomes. This gives us more confidence in suggesting the intervention option that is likely to be consistently more effective.

The model has specific structural and parameter assumptions which are fully described in (12). We also provide a full description of the transmission model and assumptions on mechanisms tracking the transmission of falciparum malaria in the accompanying paper for MINT version 1.0 (Sherrard-Smith et al. 2022). Some of the major assumptions are listed below, though the above references should be consulted for further clarification.

1.5.1 Major model assumptions

- Outputs are restricted to falciparum malaria and parameterised using data from the African continent. Projections for outside this region should be treated with caution, or where *P. falciparum* is not the dominant malaria parasite, though trends in efficacy and cost-effectiveness are likely to be consistent.
- The model is not designed for capturing malaria dynamics in near-elimination settings, where transmission is highly influenced by local heterogeneity in mosquito abundance and disease importation. Care should be taken interpreting differences in strategies when disease prevalence is less than 1%.
- We assume the average age of the population is 21 years and that the demographic structure of the population is represented by an exponential with this average age (see WorldPop.org; (13)). This average age is based on the population age structure of Tanzania but well represented in many other settings.
- A Fourier function is used to describe the 'seasonal' setting in MINT which consists of a single peak in mosquito abundance. Impact of interventions will vary if seasonality is substantially different from this, though trends are likely to be consistent.
- The model behind the MINT simulations is individual-based and stochastic. Each run is currently a single iteration of this model which will contain some random variability, though this variability is not reflected in the MINT interface, and rerunning the model with the same inputs will always generate the same results. A large population size of 100,000 people is

simulated to reduce the random variability, though small differences in malaria prevalence and cases averted should not be overly interpreted.

- The model underlying MINT assumes the presence of three species of Anopheline mosquito, with distinct characteristics which cannot be changed in the interface. For example, the percentage of mosquito bites taken between people going inside a house and before they go to bed. The overall value for these characteristics is an average of the three species-specific values, weighted by the modelled relative abundance of each species: *Anopheles gambiae sensu strict.* (50%), *Anopheles arabiensis* (25%) and *Anopheles funestus* (25%).
- The level of pyrethroid resistance remains constant throughout the 3 year period under investigation.

1.5.2 Intervention assumptions

- ITNs are modelled to be distributed, overnight, to the percentage of people indicated. This value is assumed to be the proportion of the population using nets each night immediately after the mass campaign. We assume that people stop using nets over time so that about half as many people are still using nets three years after the initial campaign as compared to the initial usage level.
- We assume that ITNs are distributed at random to the population, and across age-groups. The same assumption is made for IRS. Where ITNs and IRS are both deployed, we assume that there is correlation between interventions, meaning the same individuals receive both interventions while any difference in coverage between interventions is distributed randomly amongst the remaining population. This is because some houses are assumed to be ‘hard to find’ within the population. Different use patterns to this may change the added benefit of combining interventions.
- All ITNs are assumed to be distributed through mass campaigns, with routine ITN distribution through schools or antenatal clinics not considered.
- We assume that, previously (past vector control), all nets in use were pyrethroid-only ITNs. It is assumed that the new campaign nets replace these (if the percentage of people using nets is greater than the historic net use estimate) and are immediately adopted by the recipient.
- We do not consider difference between products of the same type. For example, all pyrethroid-PBO ITNs are assumed to be identical irrespective of the brand.
- We assume ITNs and IRS have equivalent impact on mosquitoes of any species, with the killing effect of the insecticide and its propensity to deter or repel mosquitoes being constant among Anopheles species. This follows two systematic review of experimental hut data (14),(1) which were unable to distinguish species-specific effects, though species information were limited. The tool allows the user to describe the average mosquito behaviour in the site in terms of indoor or human biting and the level of pyrethroid resistance. If there is substantial difference between multiple mosquito species in the region then values selected should reflect the average malaria transmitting mosquito and reflect local species distribution. Note the tool will not capture changes in mosquito behaviour over the period of simulation, so shifting levels of pyrethroid resistance or species abundance resulting from the use of interventions will not influence results.
- It is assumed that IRS is deployed overnight whereas delivery of IRS may be spread out across recipient households over a few weeks or months. In seasonal settings this is unlikely to change the impact projected for long-lasting IRS products. For perennial settings, this could have some impact and will depend on where and how long it takes to deploy IRS across a region.

- The mosquito is assumed to remain fully susceptible to insecticide used for IRS (following parameterisation from (1)). It is assumed that the impact of the pyrrole insecticide in pyrethroid-pyrrole ITNs remains constant over time.

1.5.3 Cost assumptions

- The cost section of MINT is relatively simple and makes very few structural assumptions, so users need to explicitly consider the relationship between the parameters they enter. For example, the cost of ITN or IRS campaigns are independent of coverage achieved. In reality, there is likely to be some relationship between the number of ITNs distributed per person in a mass campaign (a parameter that is defined by the user) and the subsequent population usage, particularly if usage is to rise above 70% (15). Users should therefore be aware that the number of nets distributed per person might need to be increased if high population usage is achieved to consider this non-linear relationship.
- To estimate cost-effectiveness, MINT version 2.0 simply takes the median cases averted from the efficacy analysis, and combines this with the cost per person protected given the population size for each respective region. We consider the upper and lower bound for the efficacy by using these estimates of cases averted rather than the median but do not alter the cost estimates defined by the user. It would be possible to explore this manually within MINT by altering the price per net or net delivery in each region set up.
- The number of clinical cases averted are estimated by taking the symptomatic incidence rate (cases per person outputted from the model) simulated for an intervention '*I*' across three years (E_I) relative to a scenario where no intervention is implemented (E_0) and multiplying incidence rates by the population size (N) determined by the user:

$$\text{Number of cases averted per person per year} = (E_0 - E_I) \times N$$

The logistical price for net delivery (ITN_{Di}) per person i is considered to be standard for any ITN type (and can be defined by the user) and mass distributions are assumed to be done every three years for a population of size N.

The mosquito net product price per person (ITN_P) and logistical costs are inflated to acknowledge the procurement buffer (P_B) of countries buying nets. The default estimate is a 7% buffer (P_B = 1.07) although this can be adjusted.

The webtool assumes that sufficient ITNs are purchased to cover the population given the entered number of people in the region and the defined number of people per net - this is called the procurement target (P_T), the default is 1.8, but this can be defined by the user. The percentage of people using ITNs immediately following mass distribution is defined by the user and values should be selected using historical data from the region of interest. If a region achieves higher ITN coverage without changing the number of nets they procure per person or their buffer, then the cost effectiveness of the campaign increases, but the total cost for the campaign is the same as when fewer people use nets.

Cost-effectiveness differs between nets, such that the cost per mass distribution campaign (once every three years for the whole population) for net type P for the total population N is:

$$Cost_P = (ITN_{Di} + ITN_P) \times P_B \times (N / P_T)$$

- The cost of an IRS campaign includes the cost per person protected for the IRS (IRS_S) multiplied by the population size. However, this is an annual cost so must be multiplied by 3 to give a comparative estimate to the cost of a mass net distribution. Therefore, the cost per three-year campaign for IRS using product *S* is: $Cost_S$ = 3 x IRS_S x *N.* Users should be aware that increasing IRS coverage does not increase $Cost_S$ so the cost of IRS per person per year (IRS_S) should be adjusted if less than 100% of the population is targeted. Further, the cost for IRS does not inflate annually in the simulations. The user can explore different estimates if an inflated price is expected year on year.
- For simplicity, we assume no costs are saved where both ITNs and IRS are implemented together, so that for combined interventions the total cost is simply the sum of the costs.
- We are not yet considering continual net top-up campaigns such as those adopted with success in e.g. Tanzania through school-based top-up.

1.6 Validating MINT: How well can the transmission model simulate interventions?

We aimed to validate the mechanistic model simulations and assumptions relating to intervention impact using a systematic review of cluster-randomised controlled trials (cRCTs) – the gold standard method for understanding the potential epidemiological impact of interventions (Sherrard-Smith et al. 2022). This exercise adopts a framework where we estimate parameters using statistical analyses to explore entomological intervention data (1,14,16,17). We then calibrate the model to epidemiological trial data. The simulation is run and compared to the results of the cRCT. Further methods and results are shown in Sherrard-Smith et al. 2022. The framework can project malaria prevalence with different types of vector control with broadly equivalent consistency.

1.7 What size area should be considered?

A region is defined as a management unit which has similar characteristics at the population level. This could be an administration unit or province, a district or collective of villages receiving IRS. IRS is very focal and usually completed in a smaller region of a larger province or district. The model assumes that IRS is applied at random to the population so, it is more appropriate to separate regions into IRS regions or non-IRS regions for this assessment and adjust population size, and number of regions characterised, accordingly.

Similarly, regions with entirely matched entomological information, seasonality, baseline prevalence and historic interventions could be considered collectively as a single region – with the population size adjusted accordingly.

Multiple regions can be considered and can be parameterised distinctly. Once the information is entered for each individual region, the user can set the maximum budget across all regions (see **Section 4**). MINT can then determine the best combination of interventions to deploy across regions to avert the most cases over the next three years. Each region can be considered independently to explore cost-effectiveness and impact locally.

1.8 Where can it be found?

The online tool can be accessed free online:

https://mint.dide.ic.ac.uk/

2 STEP-BY-STEP USER GUIDE

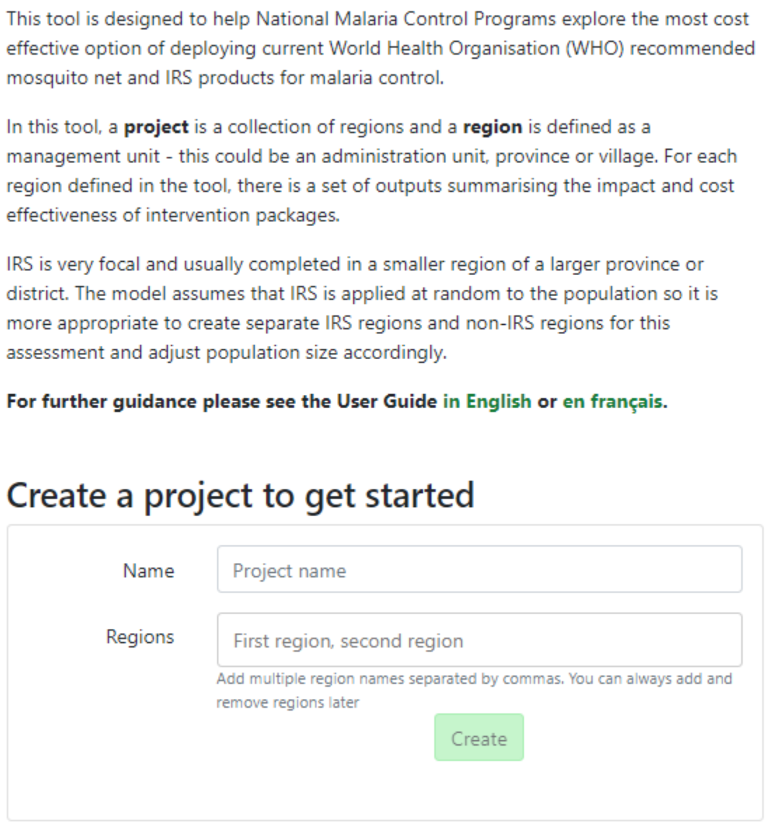

MINT allows multiple projects to be created and stored on your own personal computer. Each project is independent of all other projects previously run on the same browser (where the information on previous projects is stored). Choose a name for the project and provide a list of the different regions you want to consider. A region is defined as a management unit which will vary in geographical scale according to the question under investigation (**Section 1.7**). The analysis can consider all regions separately (as was the case in MINT Version 1.0). In addition, MINT Version 2.0 allows the impact and cost-effectiveness of different vector control intervention combinations across multiple regions to be explored. Each region requires a set of inputs describing the current situation and potential ITN and IRS usage that could be achieved in that region. You can name all regions that are to be included in the global optimisation (where interventions are **strategized across regions** to estimate the most cost-effective collection of interventions) of intervention cost-effectiveness at the initial page or by clicking on the "+Add region" tabs as you proceed. The current version of MINT the number of regions is limited to 15 for computational reasons. Please avoid just numerical names for each region.

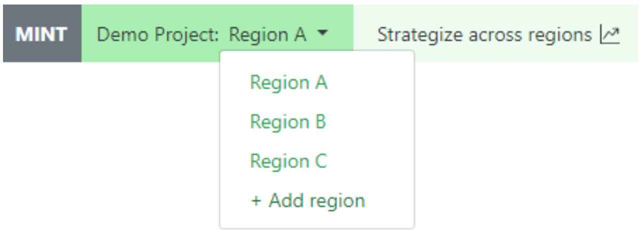

Demo region

Throughout the following sections, we will demonstrate a scenario for a region that has:

- ✓ a population of 35,400 people, and;
- ✓ seasonal transmission with approximately 30% prevalence in children under 5 years of age.

Mosquito behaviours are:

- ✓ highly endophilic,
- ✓ less anthropophilic, and;
- ✓ approximately 60% of mosquitoes are shown to be surviving exposure to a discriminatory dose bioassay (60% pyrethroid resistance).

Historically:

- ✓ about 40% of the community have been using ITNs, but;
- ✓ no IRS has been used in the region.

2.1 Setup baseline

Depending on the number of regions defined on the home page, the drop-down menu at the top-left of the next screen will contain a list of these regions by name.

Once at least 2 of these regions are selected, an additional tab will show:

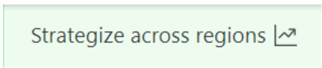

We will revisit this tab later (**Section 4**).

In **Setup baseline**, the baseline characteristics for each region can be defined.

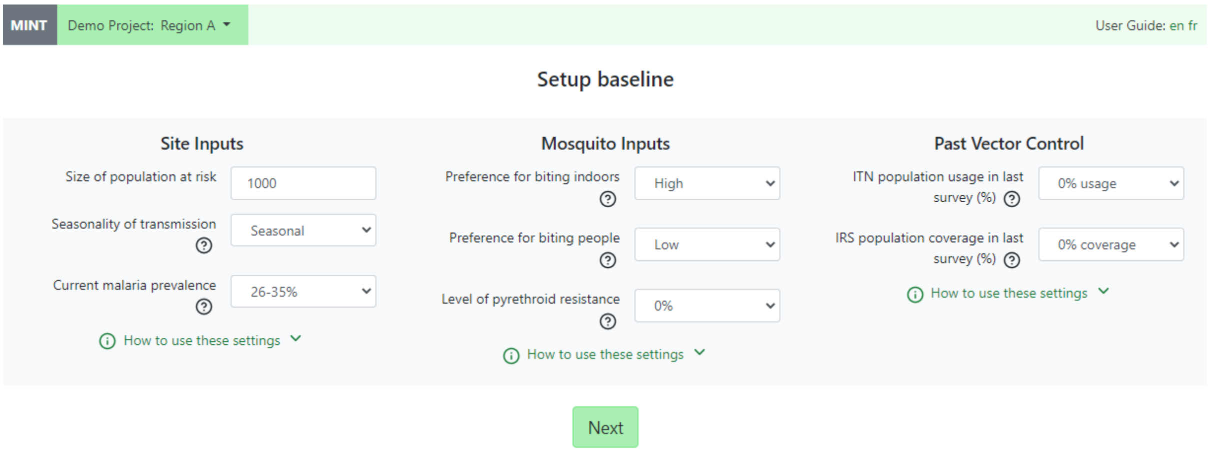

Enter the appropriate information to set up the baseline scenario that best represents each independent region. Each region is summarised using a limited number of key characteristics. Select characteristics that are most representative of the current situation based on recent local data. All inputs can be approximations though users are encouraged to experiment with multiple values to understand how different factors influence the optimal decision. Clicking on **How to use these settings** will provide additional help in filling in the form.

Site Inputs

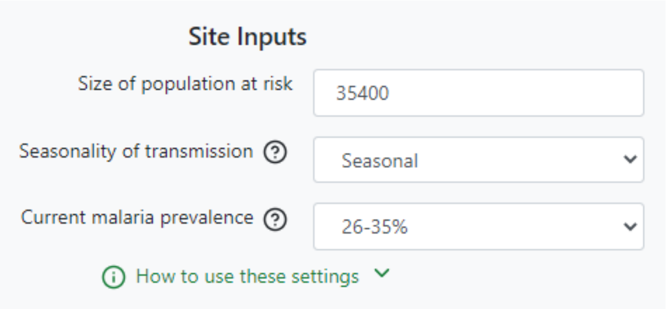

2.1.1 Size of population at risk

Enter the approximate population size of the region or sub-region to which vector control will be applied. This is only necessary if estimates of the overall cost and impact are needed. Otherwise, the default level (e.g. 1000 people) can be used.

2.1.2 Seasonality of transmission

Select **Seasonal** if the region of interest has a distinct transmission season, or **Perennial** if transmission is throughout the year.

2.1.3 Current malaria prevalence

Define the average current endemicity range for the region as measured by the percentage of children under 59 months of age (5 years) who are diagnosed with falciparum malaria by microscopy. Available options are as measured toward the end of the transmission season.

Mosquito Inputs

Values chosen here should represent the 'average' mosquito transmitting malaria throughout the year within the region. If multiple vectors are present, then the characteristics should be weighted towards the dominant vector species. For example, consider a location where two mosquito species A and B are present and are caught throughout the year at a ratio of 3:1 (i.e. Species A = 75%, Species B = 25%). If species A exhibited high levels of resistance with bioassay survival of 80% whilst species B was completely susceptible then the overall level of resistance explored could be 60% - 80% (0.75*0.8 + 0.25*0 = 60%). The tool is designed to be flexible so a sensitivity analysis could be done should the user wish to consider a range of values.

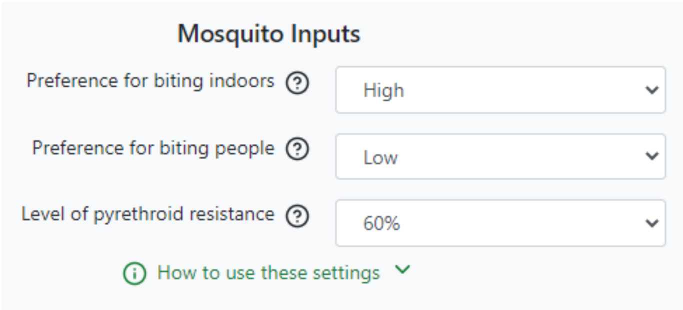

2.1.4 Preference for biting indoors

Mosquitoes may show differing propensity to bite people when they are indoors. This depends on both mosquito biting behaviour and when people go indoors. Details for calculating this quantity can be found in (16). A value of **High** indicates a model scenario that assumes 97% of bites are taken when people are indoors, whilst selecting **Low** represents 78% bites taken when people are indoors. Both scenarios can be explored by the user. These percentages represent the averaged values for the three modelled species of *Anopheles* mosquito, weighted by an assumed relative abundance: *An. gambiae s.s.* (50%), *An. arabiensis* (25%) *and An. funestus* (25%).

2.1.5 Preference for biting people

Mosquitoes show different preference for biting humans relative to other animals (often referred to as the human blood index) (17). A **High** value for the preference for biting people corresponds to a model scenario that assumes 94% of mosquito bites are taken on humans prior to introduction of interventions whilst a **Low** value assumes 61% of all bites are taken on humans. Both scenarios can be explored by the user. Again, these values are a weighted average for the three modelled *Anopheles* species, by composition: *An. gambiae s.s.* (50%), *An. arabiensis* (25%) *and An. funestus* (25%).

2.1.6 Level of pyrethroid resistance

Mosquito survival in 24-hour WHO discriminatory dose bioassays; 0% indicates all mosquitoes die and are susceptible to the pyrethroid insecticide in ITNs. 100% indicates all mosquitoes survive and are resistant to the pyrethroid insecticide in ITNs. Estimates should be adjusted taking into account mortality in the control (unexposed) mosquitoes (18,19).

Past Vector Control

The endemicity of a setting is also determined by the historic pressure from interventions that are controlling malaria transmission. This information needs to be provided for the region for both ITNs and IRS.

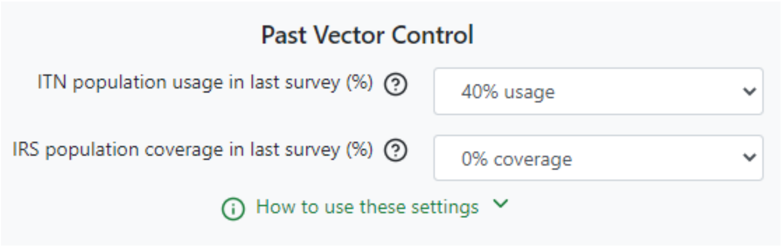

2.1.7 ITN population usage in last survey (%)

This can be estimated from Demographic Health Surveys or other surveys on net use completed in the region. The question in Malaria Indicator Surveys or Demographic Health Surveys that best reflects this parameter would be “Did you use a mosquito net last night to sleep under”, and the proportion of people answering yes to this question can be inputted. Currently MINT Version 2.0 assumes all ITNs in current use are pyrethroid-only ITNs, and it is anticipated that future updates will allow for a diversity of historical net type.

2.1.8 IRS population coverage in last survey (%)

Please choose the option that best represents the percentage of homes sprayed within the region during the last IRS campaign. If spraying has never been implemented, or has not been implemented in the most recent 4 years or more, select 0%.

2.2 Future intervention

After clicking **Next** at the bottom of the screen, enter the expected ITN use, IRS use or combination of these interventions moving forward.

(At any time, it is possible to return to the baseline parameters and explore alternative options by clicking on **Setup baseline** at the top of the screen.)

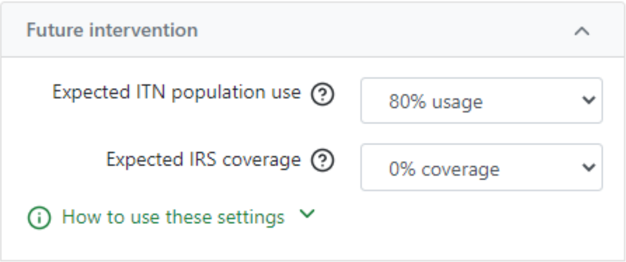

2.2.1 Expected ITN population use

Enter the expected ITN usage (of any net type: pyrethroid-only ITN, pyrethroid-PBO ITN or pyrethroid-pyrrole ITN) of people in the community immediately after the mass net distribution campaign. This will determine the intervention efficacy (and cost-effectiveness) over the next period and can be based on what usage was achieved after the last mass campaign (and can be assessed by local or Demographic Health Surveys). This value can be altered to explore how cost-effectiveness might change should more people use nets, for example. Only one net type is implemented across

the region and the model assumes there is a loss of ITN use over time since the mass campaign (see assumptions and limitations **Section 1.5.2**). This loss accounts for both the waning efficacy of the active ingredient and the waning adherence to ITN use (as nets are discarded).

2.2.2 Expected IRS coverage

Indoor residual spraying can be added to a region instead of, or in addition to, ITNs (of any type). Houses to receive IRS are assumed to correlate with those receiving nets. This is because some houses are assumed to be 'hard to find' within the population. IRS coverage estimates represent the percentage of the population living in houses with IRS. Care should be taken interpreting results as IRS is often highly clustered within small geographical areas. The model projects the estimated impact of a long-lasting IRS product (for example Actellic 300CS or Sumishield, using parameters from (1)) and where spraying is repeated annually prior to the peak of the transmission season (if seasonal setting was selected in **Section 2.1.2**).

2.3 Procurement and distribution

The decisions regarding ITN procurement and delivery might change between countries and regions. Answer the following questions so that the price estimates for impact can be augmented appropriately. Each region can take different procurement and distribution data.

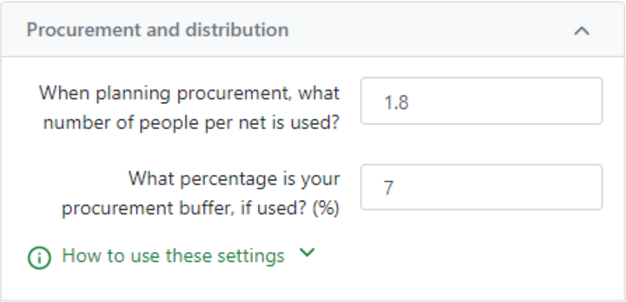

2.3.1 When planning procurement, what number of people per net is used?

The default estimate of 1.8 people per net is most commonly cited as the number used for planning mass distributions of nets. Please change as necessary. Note that the tool does not include a cap on the number of nets per household.

2.3.2 What percentage is your procurement buffer, if used? (%)

When ITNs are procured, there is a buffer to ensure there is not a short fall. Please indicate your estimate here. This is used to adjust cost estimates. The default is 7%.

2.4 Price of interventions

The price of different vector control interventions will vary over time, according to the size of orders and specifications. Here quoted prices can be defined in United States Dollars ($USD). For simplicity, we do not consider inflation during the three-year period. Costs of the product and of its delivery are separated. All ITN products within the same World Health Organisation product class are assumed to have the same epidemiological impact.

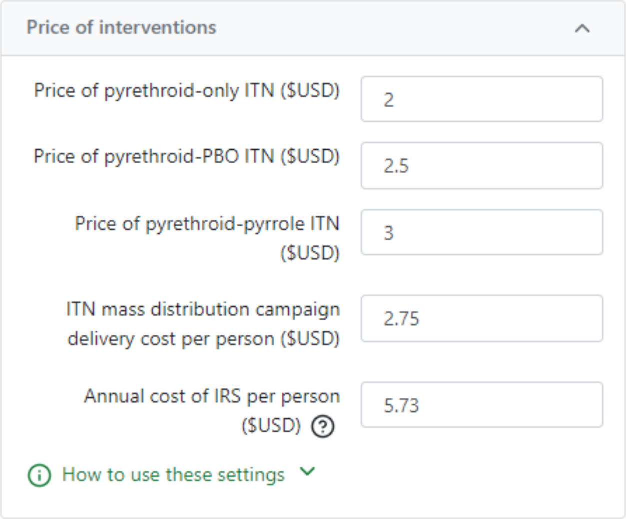

2.4.1 Price of pyrethroid-only ITN ($USD)

Price per pyrethroid-only ITN. The default is set at $2.0 USD.

2.4.2 Price of pyrethroid-PBO ITN ($USD)

Price per pyrethroid-PBO ITN. The default is set at $2.5 USD.

2.4.3 Price of pyrethroid-pyrrole ITN ($USD)

Price per pyrethroid-pyrrole ITN. The default is set at $3.0 USD.

2.4.4 ITN mass distribution campaign delivery cost per person ($USD)

Cost to deliver nets to each person (equivalent for each ITN type). Enough nets are provided to match the number of people per net (**Section 2.3.1**) and the procurement buffer (**Section 2.3.2**).

2.4.5 Annual cost of IRS per person ($USD)

The price per person of long-lasting IRS product averaged for each year. Include the average cost for both the IRS product and implementation of IRS. If different IRS products are used in different years, please average the product costs and provide an annual cost per person protected by IRS (in $USD).

3 SINGLE REGION INTERPRETATION

There are 4 output tabs in MINT for each region.

(1) Impact
(2) Cost-Effectiveness
(3) Graphs
(4) Table

Select any combination of impact/cost-effectiveness and graphs/table to display results. For impact values (table and graphs), absolute estimates are given along with values 10% lower and higher of the absolute estimate (displayed by hovering the cursor over the bar chart or table values). Real uncertainty estimates are not available in this version of MINT but will be incorporated into future iterations.

3.1 Impact graphs

The impact graphs show the estimated prevalence in the region over time, and the cases averted per 1,000 people across the three years since implementing an intervention package. The graphs present the different intervention scenarios for the specified region without considering cost. The graph display and zoom can be controlled using the buttons in the top right corner of each graph.

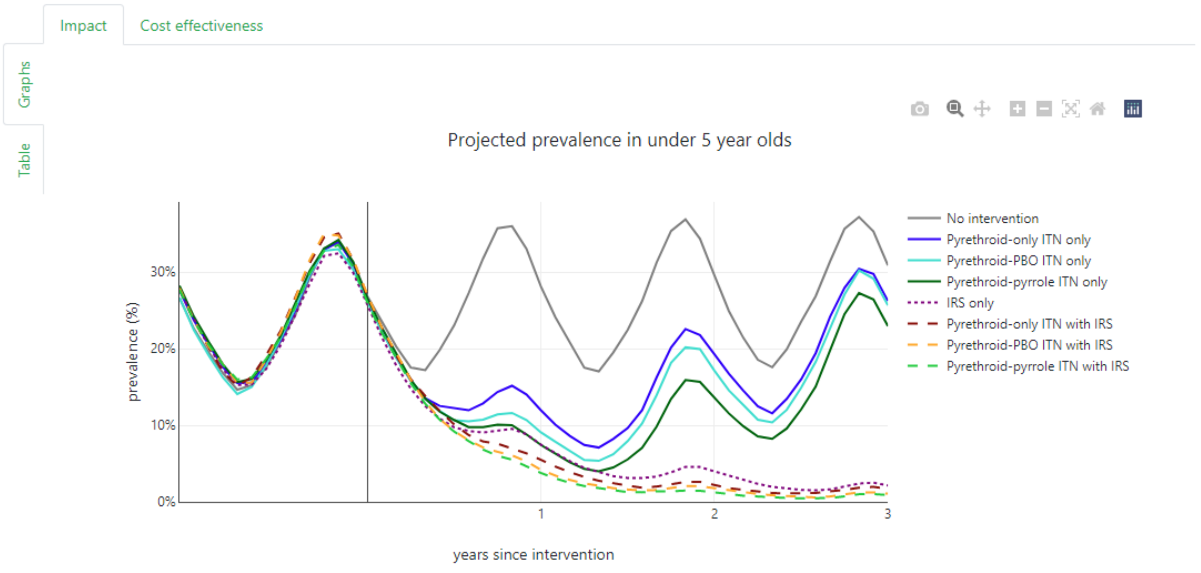

The top graph shows projected changes in prevalence of malaria in children <59 months of age (diagnosed by microscopy) for each future intervention package introduced at time 0 (vertical grey line). Graph lines for ITNs and IRS will show only if the intervention is selected to have a coverage >0% in **Future intervention**. Hovering over graph lines will display the projected prevalence for this intervention at the given time point.

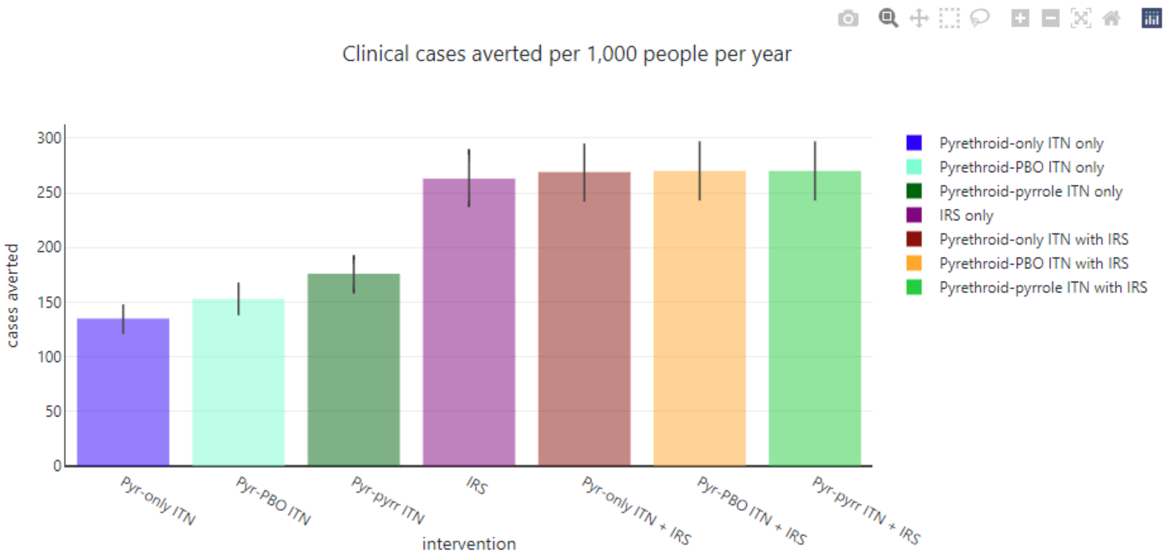

The bottom bar chart shows the number of clinical cases averted per 1,000 people averaged over three years for the different intervention packages outlined above (relative to the 'do-nothing' scenario). Hover the cursor over the bars to see absolute estimates and +/- 10% values (also represented by vertical error bars) for the impact of the strategies. Click on **How to interpret these figures** for more information.

3.2 Impact table

All data presented in the impact graphs are also summarised in the impact table tab, with the addition of relative measures of prevalence reduction and cases averted. This format allows different summary measures to be examined over the three-year time period. The table can be ordered according to the users metric of preference by clicking on the arrows on the different columns. Hovering the cursor over the values in the table will show absolute estimates and +/- 10% uncertainty for the impact of the strategies. Click on **How to interpret these figures** for more information.

Interventions	Net use (%)	IRS cover (%)	Prevalence under 5 years: Year 1 post intervention	Prevalence under 5 years: Year 2 post intervention	Prevalence under 5 years: Year 3 post intervention	Relative reduction in prevalence across 36 months post intervention	Mean cases averted per 1,000 people annually across 3 years since intervention	Relative reduction in clinical cases across 3 years since intervention	Mean cases per person per year averaged across 3 years
No intervention	n/a	n/a	26%	27%	27%	0%	0	0%	0.273
Pyrethroid-only ITN only	80%	n/a	12%	19%	16%	40%	135	49%	0.138
Pyrethroid-PBO ITN only	80%	n/a	10%	18%	16%	46%	153	56%	0.120
Pyrethroid-pyrrole ITN only	80%	n/a	8%	15%	13%	55%	176	65%	0.097
IRS only	n/a	80%	3%	1%	0%	95%	271	99%	0.002
Pyrethroid-only ITN with IRS	80%	80%	2%	1%	0%	96%	272	100%	0.001
Pyrethroid-PBO ITN with IRS	80%	80%	2%	1%	0%	96%	272	100%	0.001
Pyrethroid-pyrrole ITN with IRS	80%	80%	2%	0%	0%	97%	272	100%	0.001

How to interpret these figures

3.3 Cost effectiveness graphs

The cost effectiveness tab shows the estimated total cost in $USD of each intervention strategy over three years against the expected total number of cases averted per 1,000 people across three years (top graph) and costs per case averted for each intervention strategy (bottom graph). The graph display and zoom can be controlled using the buttons in the top right corner of each graph.

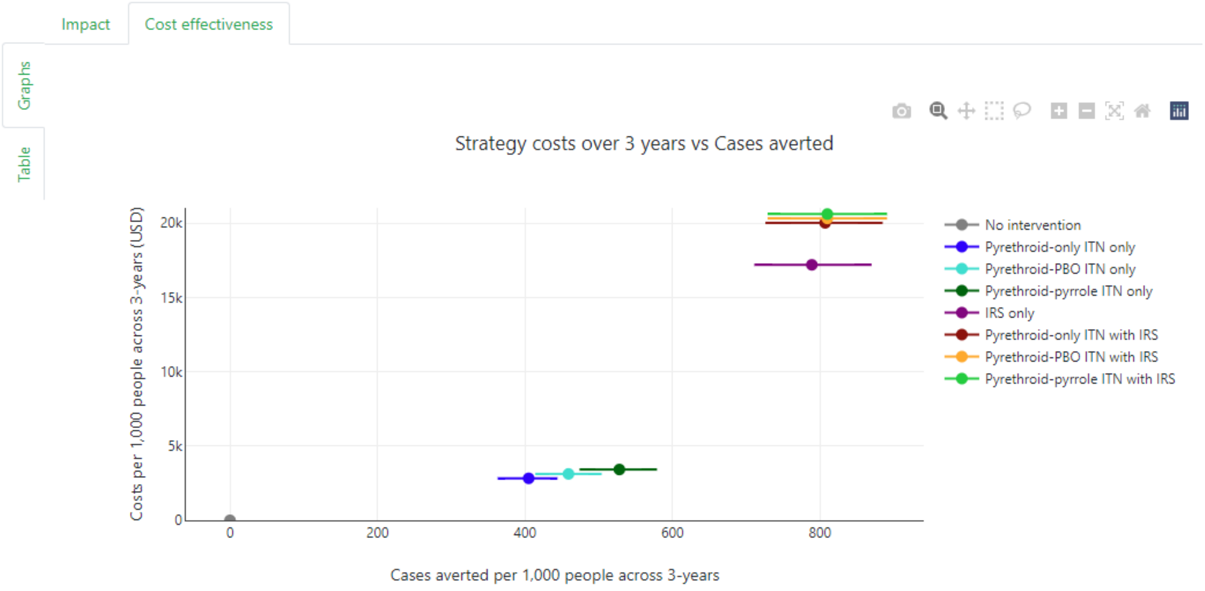

In the top graph, the intervention strategies furthest to the top-right of the graph are the most effective but also most expensive. Consider which strategies are most effective within your available budget. The most cost-effective strategy will depend on the ecologies and assumptions made about the region and the price of each ITN. It may be useful to explore different estimates and see how the simulated outcomes are altered, where there is uncertainty in these ecological assumptions.

Points show the best estimate for projected impact whilst the horizontal lines at these points indicate uncertainty in intervention impact driven by the statistical analysis of the interventions ((14) & Sherrard-Smith et al. 2022). Uncertainty in costs are not provided in this version of MINT, but the user can explore changes in these costs by altering the inputs in the costs section (**Section 2.4**).

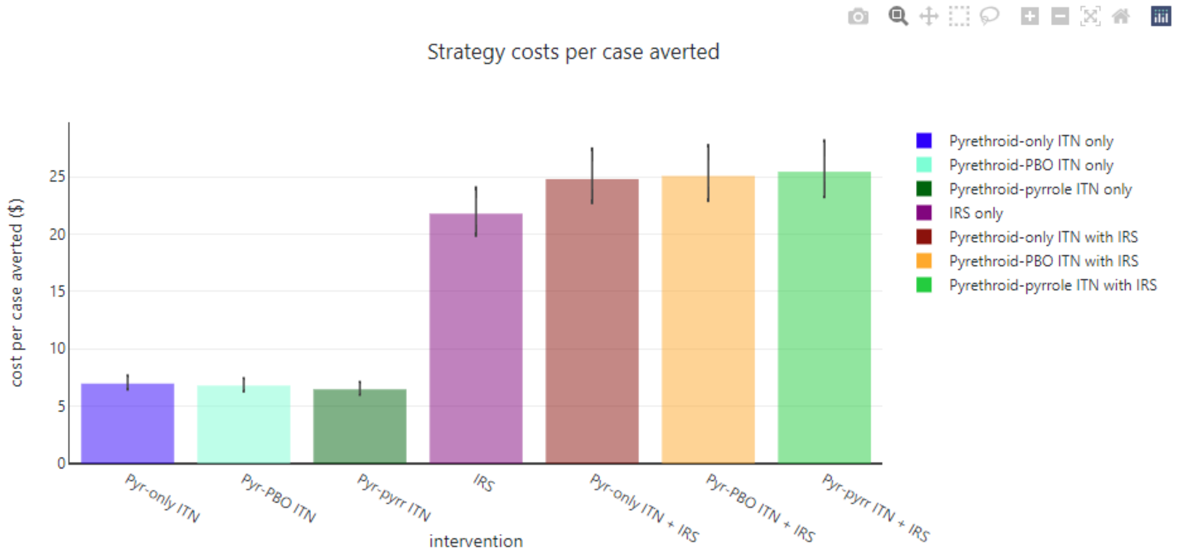

In the bottom graph, the cost per case averted is displayed for each intervention strategy. A taller bar suggests a more cost-effective intervention strategy. Hover the curse over each bar to display the absolute estimate and uncertainty values for each intervention strategy.

Summary information provided by the user indicates the expected ITN usage and IRS coverage to be achieved in the region. Altering these would alter the cost-effectiveness and the user is encouraged to explore this feature. Click on **How to interpret these figures** for more information.

3.4 Cost effectiveness table

The cost effectiveness table provides the numbers for the different measures projected by the model. As with the impact table the different intervention packages can be ordered according to the different metrics by clicking on the arrows in each column and uncertainty values can be displayed by hovering the cursor over each value. Click on **How to interpret these figures** for more information.

Interventions	Net use (%)	IRS cover (%)	Total cases averted	Total costs	Cost per case averted across 3 years
No intervention	n/a	n/a	0	$0	reference
Pyrethroid-only ITN only	80%	n/a	14292	$99.96k	$7.00
Pyrethroid-PBO ITN only	80%	n/a	16257	$110.5k	$6.80
Pyrethroid-pyrrole ITN only	80%	n/a	18678	$121k	$6.50
IRS only	n/a	80%	28761	$608.5k	$21.20
Pyrethroid-only ITN with IRS	80%	80%	28875	$708.5k	$24.50
Pyrethroid-PBO ITN with IRS	80%	80%	28863	$719k	$24.90
Pyrethroid-pyrrole ITN with IRS	80%	80%	28905	$729.5k	$25.20

How to interpret these figures

4 MULTIPLE REGION ANALYSIS

It is likely that it will be beyond a country's budget to do the most effective interventions everywhere. As the burden of malaria and the historical use of control interventions will differ between regions it is not clear how resources and interventions should be allocated to maximise impact. The complexity of the disease and the differential impact of interventions means that better interventions cannot simply be allocated to higher burden areas. For example, some regions might currently have a low malaria burden given high intervention use, and the disease will resurge if new interventions are not deployed.

Version 2.0 of MINT can suggest options for how resources and interventions can be allocated across a user-defined set of regions given a total budget. The optimiser that sits behind MINT's interface determines the maximum cases that can be averted across all regions whilst staying within the user-defined maximum budget. The optimal decision selected by MINT should be considered considering other local factors, such as equity, insecticide resistance management or logistics, which could influence the ultimate optimal decision. Currently, interventions are allocated according to cases averted, so in some scenarios, regions might be left without protection if there is high variability in disease endemicity (big differences in prevalence prior to introduction of control interventions) and limited budgets.

Once the regional data are entered into each option, click on **Strategize across regions** to begin a multiple region analysis:

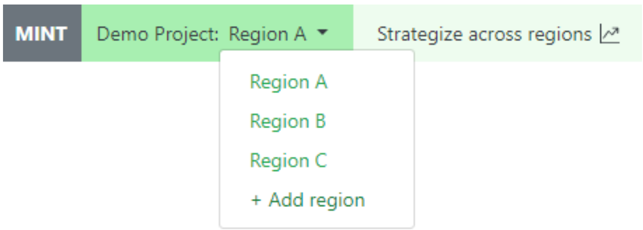

On the first screen, a total available budget for all regions can be set:

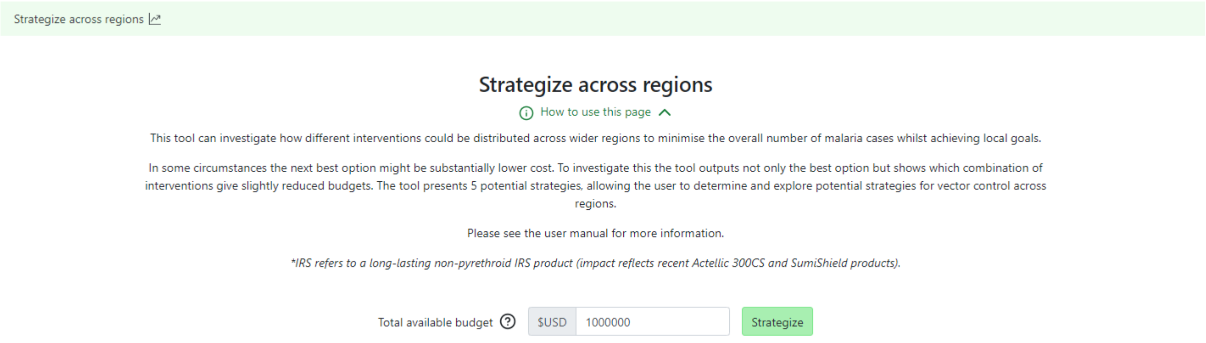

Clicking **Strategize** will show five potential strategies in a table;

1. The best solution within budget
2. The best solution whilst spending 95% of the budget
3. The best solution whilst spending 90% of the budget

4. The best solution whilst spending 85% of the budget
5. The best solution whilst spending 80% of the budget

This allows the user to determine and explore potential strategies for vector control across regions. (Sometimes, the same solution is within, for example, 90% of the budget, as it may not be possible to achieve a better outcome [more cases averted] simply by increasing spending in any region beyond a given budget.) The total budget can be adjusted again to see how this may change the available options. We would advise this to see how much might be required to increase protection for a given region with an incremental increase in spend.

4.1 Strategy interpretation

The five strategies are shown as rows in a table. For each strategy, the best intervention package to deploy is shown for each region, as consecutive columns (in our example, these are Region A, Region B and Region C). Total cases averted, across all regions, and the total cost across all regions, are calculated.

For example, the images below demonstrate the different set of interventions that might be achievable in three regions when increasing a budget from $USD 500,000 to $USD 1,300,000:

Total available budget ? $USD 500000 Strategize

All strategies

	Maximum Cost Vs Budget	Region A	Region B	Region C	Total Cases Averted	Total Cost
**Strategy 1**	100%	Pyrethroid-PBO ITN only	Pyrethroid-pyrrole ITN only	Pyrethroid-pyrrole ITN only	76,788	$489,882
**Strategy 2**	95%	Pyrethroid-PBO ITN only	Pyrethroid-pyrrole ITN only	Pyrethroid-PBO ITN only	74,121	$469,581
**Strategy 3**	90%	Pyrethroid-PBO ITN only	Pyrethroid-only ITN only	Pyrethroid-PBO ITN only	69,282	$444,199
**Strategy 4**	85%	Pyrethroid-PBO ITN only	Pyrethroid-only ITN only	Pyrethroid-only ITN only	62,397	$423,898
**Strategy 5**	80%	No intervention	Pyrethroid-pyrrole ITN only	Pyrethroid-pyrrole ITN only	60,531	$379,404

Select a row in the table above to see details of the selected strategy

Total available budget ? $USD 1300000 Strategize

All strategies

	Maximum Cost Vs Budget	Region A	Region B	Region C	Total Cases Averted	Total Cost
**Strategy 1**	100%	Pyrethroid-pyrrole ITN only	Pyrethroid-pyrrole ITN with IRS	Pyrethroid-pyrrole ITN only	96,666	$1,234,416
**Strategy 2**	95%	Pyrethroid-pyrrole ITN only	Pyrethroid-pyrrole ITN with IRS	Pyrethroid-pyrrole ITN only	96,666	$1,234,416
**Strategy 3**	90%	Pyrethroid-pyrrole ITN only	IRS only	Pyrethroid-pyrrole ITN only	95,421	$1,088,465
**Strategy 4**	85%	Pyrethroid-pyrrole ITN only	IRS only	Pyrethroid-pyrrole ITN only	95,421	$1,088,465
**Strategy 5**	80%	IRS only	Pyrethroid-pyrrole ITN only	Pyrethroid-pyrrole ITN only	88,482	$987,930

Select a row in the table above to see details of the selected strategy

In the mechanistic framework that underpins MINT, the best current intervention package – the one that could avert most cases – is the combined use of pyrethroid-pyrrole ITNs and IRS. This is because the efficacy of the pyrethroid-pyrrole ITNs was found to be best in cRCTs and our model parameters reflect this. Combining the nets with indoor residual spraying corresponds to fewer infectious bites in the transmission model regardless of the net deployed, so the modelling result is fewer cases of malaria. In our example, using the default price of $3.00 for the pyrethroid-pyrrole ITN, this combination is the most expensive. In the example with the higher budget, we see that Region B can receive this optional combination whereas it is only possible to distribute pyrethroid-pyrrole ITNs alone in the example with the lower budget for this region.

We see that the strategy for maximum spend within budget (top row of the table), can avert an estimated 96,666 cases with this set of intervention combinations across regions. However, we could still avert 88,482 cases and spend approximately USD $250,000 less by changing interventions in Region A and Region B (Strategy 5). We strongly encourage users to explore multiple examples and stress that we have not yet included uncertainty here.

Click on any of the strategy rows (1-5) to explore the individual regional data and breakdown of what impact the interventions are projected to have at the regional level. There are 2 outputs tabs in the tool for each strategy:

(1) Charts
(2) Table

4.2 Strategize charts

Two summary graphs are shown to contrast the impact and cost-effectiveness in different regions.

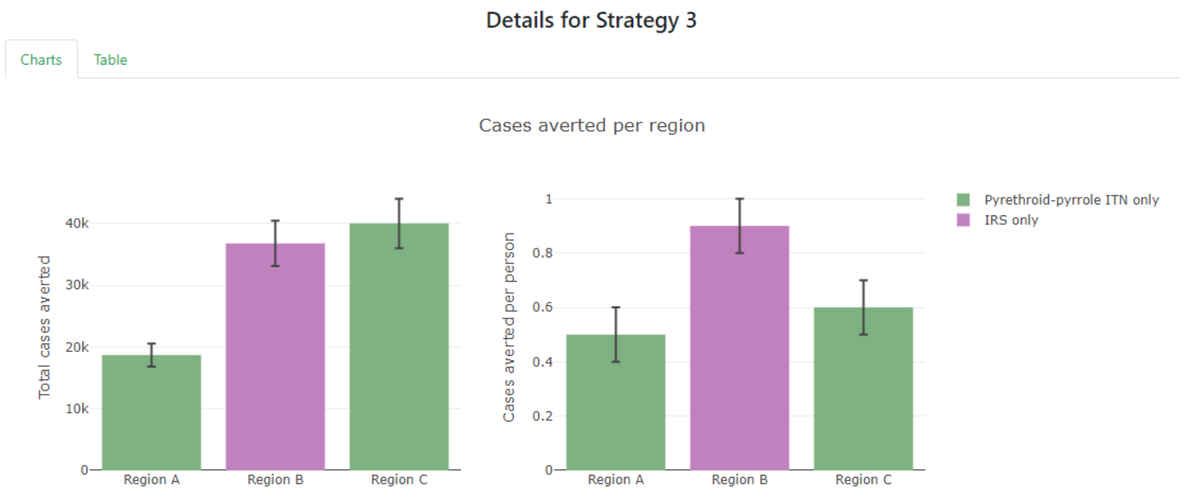

In the example here, we have clicked on Strategy 3 (the third row in the table), which has suggested distribution of pyrethroid-pyrrole ITNs for Region A and Region C and IRS only for Region B.

In the first panel, the total cases averted in each region across the three-year campaign period are coloured by the intervention package ascribed to each region. These are dependent on the population size indicated for each region and the optimal solution for deploying the collective interventions across regions.

The second panel shows the cases averted per person across the three-year campaign.

4.3 Strategize table

The table shows the region-level data for the strategy selected:

Population: The user-selected population size of the region or sub-region to which vector control will be applied.

Total cases averted: The cases averted (all-age) across three years since the start of intervention that is projected to be achieved in each region; the absolute number of clinical cases averted given the population size and relative to the 'do-nothing' scenario. The column total will sum to the total cases averted across the three years across all regions.

Percentage of total cases averted: The percentage of cases averted across each region that are projected to be achievable. The column total will sum to 100%.

Total costs: The total cost in $USD expected for the product procurement and implementation for the intervention package to cover a three-year period of protection for each region. The column total will show the total cost required for this strategy.

Percentage of total costs: The percentage of costs required for each region out of the total budget. The column total will sum to 100%.

Cost per case averted: The cost in $USD per case averted across the three years relative to the 'do-nothing' scenario. This allows comparison between regions of cost-effectiveness as measured by cost per case averted per population.

Cost per person: The cost in $USD per person for the respective intervention.

Cases averted per person: The cases averted per person across the three-year campaign for each region.

Details for Strategy 3

Charts | Table

Region	Intervention	Population	Total Cases Averted	Percentage Of Total Cases Averted	Total Costs	Percentage Of Total Costs	Cost Per Case Averted	Cost Per Person	Cases Averted Per Person
Region A	Pyrethroid-pyrrole ITN only	35400	18,678	19.6%	$120,999	11.1%	$6	$3.42	0.5
Region B	IRS only	42700	36,753	38.5%	$734,013	67.4%	$20	$17.19	0.9
Region C	Pyrethroid-pyrrole ITN only	68300	39,990	41.9%	$233,453	21.4%	$6	$3.42	0.6
**Total**		**146400**	**95,421**	**100%**	**$1,088,465**	**100%**			

The user can switch to explore the projections from a different strategy to understand what can be gained from slight reductions in the maximum budget.

Return to the regional tabs if input parameters need to be adjusted. Then click again on **Strategize across regions** to recalculate the best deployment strategy across multiple regions.

We hope the tool is of use and welcome and encourage any feedback from those using MINT v2.0

e.sherrard-smith@imperial.ac.uk, thomas.churcher@imperial.ac.uk

MINT methodology

A number of publications underlie the methodology that supports MINT. These include articles that have analysed data with which to parameterise the transmission model and validation exercises (this page) as well as articles describing the underlying model itself (following page).

Data analysis papers

- *Nash et al. 2021 Systematic review of the entomological impact of insecticide-treated nets evaluated using experimental hut trials in Africa. Current Research in Parasitology and Vector-Borne Diseases 1: e100047*

This first article presents the systematic review of entomological data including World Health Organization discriminatory dose susceptibility bioassays, the Centre for Disease Control (USA) discriminatory dose susceptibility (tube and bottle) bioassays, and experimental hut trial assays. The statistical analyses are used to determine intervention parameters for the transmission model used in MINT.

- *Killeen et al. 2017 Going beyond personal protection against mosquito bites to eliminate malaria transmission: population suppression of malaria vectors that exploit both human and animal blood. BMJ Global Health 2: e000198. Doi: 10.1136/bmjgh-2016-000198*
- *Sherrard-Smith et al. 2019 Mosquito feeding behaviour and how it influences residual malaria transmission across Africa. PNAS 116: 15086-15096. Doi: 10.1073/pnas.1820646116*

We also use systematic review data analyses from two previous papers to help parameterise the model underpinning MINT. The second article summarises our understanding of the distribution in human blood feeding among mosquito species. We use these analyses to inform the 'high' and 'low' options offered in the MINT interface.

The third article summarises our understanding of the proportion of mosquito feeding attempts that continue even when indoor mosquito interventions are optimally deployed and used. We use these analyses to inform the 'high' and 'low' options offers in the MINT interface.

- *Sherrard-Smith et al. 2022 Inferring the epidemiological benefit of indoor vector control interventions against malaria from mosquito data. Nature Communications 13: 3862. Doi: 10.1038/s41467-022-30700-1*

This fourth paper is a validation exercise where we demonstrate the ability of the transmission model, specifically parameterised to various ecological settings, to project the observed prevalence in cohorts of children that have received indoor vector control interventions as tested in cluster-randomised controlled trials. We are reassured that the model simulations are appropriately able to recreate public health impact of pyrethroid-only and pyrethroid-PBO insecticide treated nets, and indoor residual spraying of insecticides accurately (adjusted $R^2 > 0.9$ for the collective data from 14 trials representing 37 trial arms and 73 cross-sectional surveys).

Transmission dynamic modelling papers

- *Sherrard-Smith et al. 2022 Optimising the deployment of vector control tools against malaria: a data-informed modelling study. The Lancet Planetary Health 6: e100-109*

This article builds on the parameter estimates from Nash et al (2021) and explores the added benefit of using pyrethroid-PBO mosquito nets. This paper specifically accompanies MINT version 1.0 and presents the full analyses and data combinations that underpin the results of the webtool.

- *Churcher et al. 2021 in prep. Projecting epidemiological benefit of pyrethroid-pyrrole insecticide treated nets against malaria:*

This paper introduces the parameterisation of pyrethroid-pyrrole ITNs and determines the algorithm to explore the combination of various interventions across locations.

We also use a succession of papers that have iteratively improved the transmission model since it was first published in 2010 (Griffin et al 2010). The critical papers for consideration, with full mathematical descriptions of the model, include:

- *Griffin et al. 2010 Reducing Plasmodium falciparum malaria transmission in Africa: A model-based evaluation of intervention strategies. PLoS Medicine 7: e1000324*
- *White et al. 2011 Modelling the impact of vector control interventions on Anopheles gambiae population dynamics. Parasites & Vectors 4: 153*
- *Griffin et al. 2014. Estimates of the changing age-burden of Plasmodium falciparum malaria disease in sub-Saharan Africa. Nature Communications 5: 3136*
- *Griffin et al. 2016 Potential for reduction of burden and local elimination of malaria by reducing Plasmodium falciparum malaria transmission: a mathematical modelling study. The Lancet Infectious Diseases 16: 465-472*
- *Churcher et al. 2016 The impact of pyrethroid resistance on the efficacy and effectiveness of bednets for malaria control in Africa. eLife 5: e16090*
- *Winskill et al. 2017 The US President's Malaria Initiative, Plasmodium falciparum transmission and mortality: A modelling study PLoS Med 14: Article e1002448*
- *Sherrard-Smith et al. 2018 Systematic review of indoor residual spray efficacy and effectiveness against Plasmodium falciparum in Africa. Nature Communications 9: 4982*
- *Charles G, et al. 2022. Malariasimulation: An individual based model for malaria. R package version 1.4.1.*

Code

The model used to simulate the examples underpinning the MINT interface can be found here:

https://mrc-ide.github.io/malariasimulation/

References

1. Sherrard-Smith E, Griffin JT, Winskill P, Corbel V, Pennetier C, Djénontin A, et al. Systematic review of indoor residual spray efficacy and effectiveness against Plasmodium falciparum in Africa. Nat Commun. 2018 Dec 26;9(1):4982.

2. Protopopoff N, Mosha JF, Lukole E, Charlwood JD, Wright A, Mwalimu CD, et al. Effectiveness of a long-lasting piperonyl butoxide-treated insecticidal net and indoor residual spray interventions, separately and together, against malaria transmitted by pyrethroid-resistant mosquitoes: a cluster, randomised controlled, two-by-two fact. The Lancet. 2018 Apr 21;391(10130):1577–88.

3. Staedke SG, Gonahasa S, Dorsey G, Kamya MR, Maiteki-Sebuguzi C, Lynd A, et al. Effect of long-lasting insecticidal nets with and without piperonyl butoxide on malaria indicators in Uganda (LLINEUP): a pragmatic, cluster-randomised trial embedded in a national LLIN distribution campaign. The Lancet. 2020;395(10232):1292–303.

4. Accrombessi M, Cook J, Dangbenon E, Yovogan B, Akpovi H, Sovi A, et al. Efficacy of pyriproxyfen-pyrethroid long-lasting insecticidal nets (LLINs) and chlorfenapyr-pyrethroid LLINs compared with pyrethroid-only LLINs for malaria control in Benin: a cluster-randomised, superiority trial. The Lancet. 2023 Feb 11;401(10375):435–46.

5. Mosha JF, Kulkarni MA, Lukole E, Matowo NS, Pitt C, Messenger LA, et al. Effectiveness and cost-effectiveness against malaria of three types of dual-active-ingredient long-lasting insecticidal nets (LLINs) compared with pyrethroid-only LLINs in Tanzania: a four-arm, cluster-randomised trial. The Lancet. 2022 Mar 26;399(10331):1227–41.

6. Hancock PA, Hendriks CJM, Tangena JA, Gibson H, Hemingway J, Coleman M, et al. Mapping trends in insecticide resistance phenotypes in African malaria vectors. PLoS Biol. 2020;18(6):1–23.

7. Matowo NS, Munhenga G, Tanner M, Coetzee M, Feringa WF, Ngowo HS, et al. Fine-scale spatial and temporal heterogeneities in insecticide resistance profiles of the malaria vector, Anopheles arabiensis in rural south-eastern Tanzania. Wellcome Open Res. 2017;2:96.

8. Mzilahowa T, Chiumia M, Mbewe RB, Uzalili VT, Luka-Banda M, Kutengule A, et al. Increasing insecticide resistance in Anopheles funestus and Anopheles arabiensis in Malawi, 2011–2015. Malar J. 2016 Dec 22;15(1):563.

9. Uragayala S, Kamaraju R, Tiwari S, Ghosh SK, Valecha N. Small-scale evaluation of the efficacy and residual activity of alpha-cypermethrin WG (250 g AI/kg) for indoor spraying in comparison with alpha-cypermethrin WP (50 g AI/kg) in India. Malar J. 2015;14(1):223.

10. Etang J, Nwane P, Mbida J, Piameu M, Manga B, Souop D, et al. Variations of insecticide residual bio-efficacy on different types of walls: results from a community-based trial in south Cameroon. Malar J. 2011;10(1):333.

11. Opiyo MA, Paaijmans KP. 'We spray and walk away': Wall modifications decrease the impact of indoor residual spray campaigns through reductions in post-spray coverage. Malar J. 2020;19(1):1–6.

12. Walker PGT, Griffin JT, Ferguson NM, Ghani AC. Estimating the most efficient allocation of interventions to achieve reductions in Plasmodium falciparum malaria burden and transmission in Africa: a modelling study. Lancet Glob Health. 2016 Jul 1;4(7):e474-84.

13. Pezzulo C, Hornby GM, Sorichetta A, Gaughan AE, Linard C, Bird TJ, et al. Sub-national mapping of population pyramids and dependency ratios in Africa and Asia. Sci Data 2017 41. 2017 Jul 19;4(1):1–15.

14. Nash RK, Lambert B, N'Guessan R, Ngufor C, Rowland M, Oxborough R, et al. Systematic review of the entomological impact of insecticide-treated nets evaluated using experimental hut trials in Africa. medRxiv. 2021;

15. Bertozzi-Villa A, Bever C, Koenker H, Weiss DJ, Vargas-Ruiz C, Nandi AK, et al. Maps and Metrics of Insecticide-Treated Net Coverage in Africa: Access, Use, and Nets-Per-Capita, 2000-2020. 2021 Feb.

16. Sherrard-Smith E, Skarp JE, Beale AD, Fornadel C, Norris LC, Moore SJ, et al. Mosquito feeding behavior and how it influences residual malaria transmission across Africa. Proc Natl Acad Sci U S A. 2019;116(30):15086–96.

17. Killeen GF, Kiware SS, Okumu FO, Sinka ME, Moyes CL, Massey NC, et al. Going beyond personal protection against mosquito bites to eliminate malaria transmission: population suppression of malaria vectors that exploit both human and animal blood. BMJ Glob Health. 2017 Apr 26;2(2):e000198.

18. Churcher TS, Lissenden N, Griffin JT, Worrall E, Ranson H. The impact of pyrethroid resistance on the efficacy and effectiveness of bednets for malaria control in Africa. eLife. 2016 Aug 22;5.

19. World Health Organization. Malaria vector control policy recommendations and their applicability to product evaluation. 2017.
